# Instantaneous Amplitude and Frequency Modulations Detect the Footprint of Rotational Activity and Reveal Stable Driver Regions as Targets for Persistent Atrial Fibrillation Ablation

**DOI:** 10.1161/CIRCRESAHA.119.314930

**Published:** 2019-08-29

**Authors:** Jorge G. Quintanilla, José Manuel Alfonso-Almazán, Nicasio Pérez-Castellano, Sandeep V. Pandit, José Jalife, Julián Pérez-Villacastín, David Filgueiras-Rama

**Affiliations:** 1 From the Myocardial Pathophysiology Area, Centro Nacional de Investigaciones Cardiovasculares (CNIC), Madrid, Spain (J.G.Q., J.M.A.-A., J.J., D.F.-R.); 2 Arrhythmia Unit, Cardiology Department, Cardiovascular Institute, Instituto de Investigación Sanitaria del Hospital Clínico San Carlos (IdISSC), Madrid, Spain (J.G.Q., N.P.-C., J.P.-V., D.F.-R.); 3 Centro de Investigación Biomédica en Red de Enfermedades Cardiovasculares (CIBERCV), Madrid, Spain (J.G.Q., N.P.-C., J.J., J.P.-V., D.F.-R.); 4 Center for Arrhythmia Research, Department of Internal Medicine, University of Michigan, Ann Arbor (S.V.P., J.J.).

**Keywords:** ablation, algorithms, atrial fibrillation, driver, mapping, rotor

## Abstract

Costly proprietary panoramic multielectrode (64–256) acquisition systems are being increasingly used together with conventional electroanatomical mapping systems for persistent atrial fibrillation (PersAF) ablation. However, such approaches target alleged drivers (rotational/focal) regardless of their activation frequency dynamics.

**Objectives:**

To test the hypothesis that stable regions of higher than surrounding instantaneous frequency modulation (iFM) drive PersAF and determine whether rotational activity is specific for such regions.

**Methods and Results:**

First, novel single-signal algorithms based on instantaneous amplitude modulation (iAM) and iFM to detect rotational-footprints without panoramic multielectrode acquisition systems were tested in 125 optical movies from 5 ex vivo Langendorff-perfused PersAF sheep hearts (sensitivity/specificity, 92.6/97.5%; accuracy, 2.5-mm) and in computer simulations. Then, 16 pigs underwent high-rate atrial pacing to develop PersAF. After a median (interquartile range [IQR]) of 4.4 (IQR, 2.5–9.9) months of high-rate atrial pacing followed by 4.1 (IQR, 2.7–5.4) months of self-sustained PersAF, pigs underwent in vivo high-density electroanatomical atrial mapping (4920 [IQR, 4435–5855] 8-second unipolar signals per map). The first 4 out of 16 pigs were used to adapt ex vivo optical proccessing of iFM/iAM to in vivo electrical signals. In the remaining 12 out of 16 pigs, regions of higher than surrounding average iFM were considered leading-drivers. Two leading-driver + rotational-footprint maps were generated 2.6 (IQR, 2.4–2.9) hours apart to test leading-driver spatiotemporal stability and guide ablation. Leading-driver regions (2.5 [IQR, 2.0–4.0] regions/map) exactly colocalized (95.7%) in the 2 maps, and their ablation terminated PersAF in 92.3% of procedures (radiofrequency until termination, 16.9 [IQR, 9.2–35.8] minutes; until nonsustainability, 20.4 [IQR, 12.8–44.0] minutes). Rotational-footprints were found at every leading-driver region, albeit most (76.8% [IQR, 70.5%–83.6%]) were located outside. Finally, the translational ability of this approach was tested in 3 PersAF redo patients.

**Conclusions:**

Both rotational-footprints and spatiotemporally stable leading-driver regions can be located using iFM/iAM algorithms without panoramic multielectrode acquisition systems. In pigs, ablation of leading-driver regions usually terminates PersAF and prevents its sustainability. Rotational activations are sensitive but not specific to such regions. Single-signal iFM/iAM algorithms could be integrated into conventional electroanatomical mapping systems to improve driver detection accuracy and reduce the cost of patient-tailored/mechanistic approaches.

**Visual Overview:**

An online visual overview is available for this article.

## Abstract 2

Supplemental Digital Content is available in the text.

**In This Issue, see p 571**

**Meet the First Author, see p 572**

The burden of atrial fibrillation (AF) has progressively increased over the past decades worldwide with an estimated average lifetime risk of 37%.^1^ Importantly, AF increases morbidity and mortality and has important economic and social implications.^2^ Pulmonary vein (PV) isolation (PVI) is considered the cornerstone for AF ablation. However, it is associated with suboptimal clinical outcomes in persistent AF (PersAF) as other atrial regions may play a role in long-term maintenance.^3–5^ Linear lesions on the left atrium (LA) and ablation targeting complex fractionated atrial electrograms have also been attempted without incremental benefit to conventional PVI.^6^

New approaches using proprietary algorithms and panoramic multielectrode (64–256) acquisition systems (PMEAS) to identify alleged drivers (rotational or focal) have improved ablation outcomes in some series.^7,8^ More recently, another strategy targeting areas with spatiotemporal dispersion has also shown promising results.^9^ Such alleged drivers or spatiotemporal dispersion regions are ablated regardless of their activation frequency dynamics. However, such ablation approaches may be potentially unspecific because they do not consider the frequency hierarchy demonstrated in high-resolution optical mapping studies during cardiac fibrillation.^10–12^ Moreover, the sensitivity and specificity of PMEAS for detecting rotors\/foci are limited by multiple technical aspects,^13–15^ and clinical outcomes remain controversial.^16^ Furthermore, all these patient-tailored mechanistic ablation approaches assume that the detected drivers are spatiotemporally stable in the medium/long-term, although this has never been conclusively demonstrated.^17–19^ In addition, proprietary PMEAS and their ancillary expendable materials need to be used in combination with conventional electroanatomical mapping systems for ablation. Conceivably, incorporating single-signal algorithms capable of detecting rotors or/and AF drivers into standard electroanatomical mapping systems devoid of PMEAS would significantly decrease the cost associated with current patient-tailored procedures.

To overcome these constraints, we worked on the assumption that the amplitude and frequency modulations (AM/FM) used for radio broadcasting are naturally present in signals during cardiac fibrillation, although in a completely different frequency range (Figure 1A and 1B). Indeed, Doppler Effect (which is a form of FM) was observed in the ECG during ex vivo ventricular fibrillation (VF) due to drifting rotors.^20^ Clear AM around rotor cores has also been reported in optical signals during ex vivo VF.^12^ The AM concept alone was later used to develop a method for detecting rotors during VF.^21^ However, that strategy alone is not applicable to in vivo AF procedures because changes in the electrode-tissue contact due to catheter movement (ventricular contraction, catheter instability, etc) may yield overlying and unspecific AMs.

**Figure 1.**
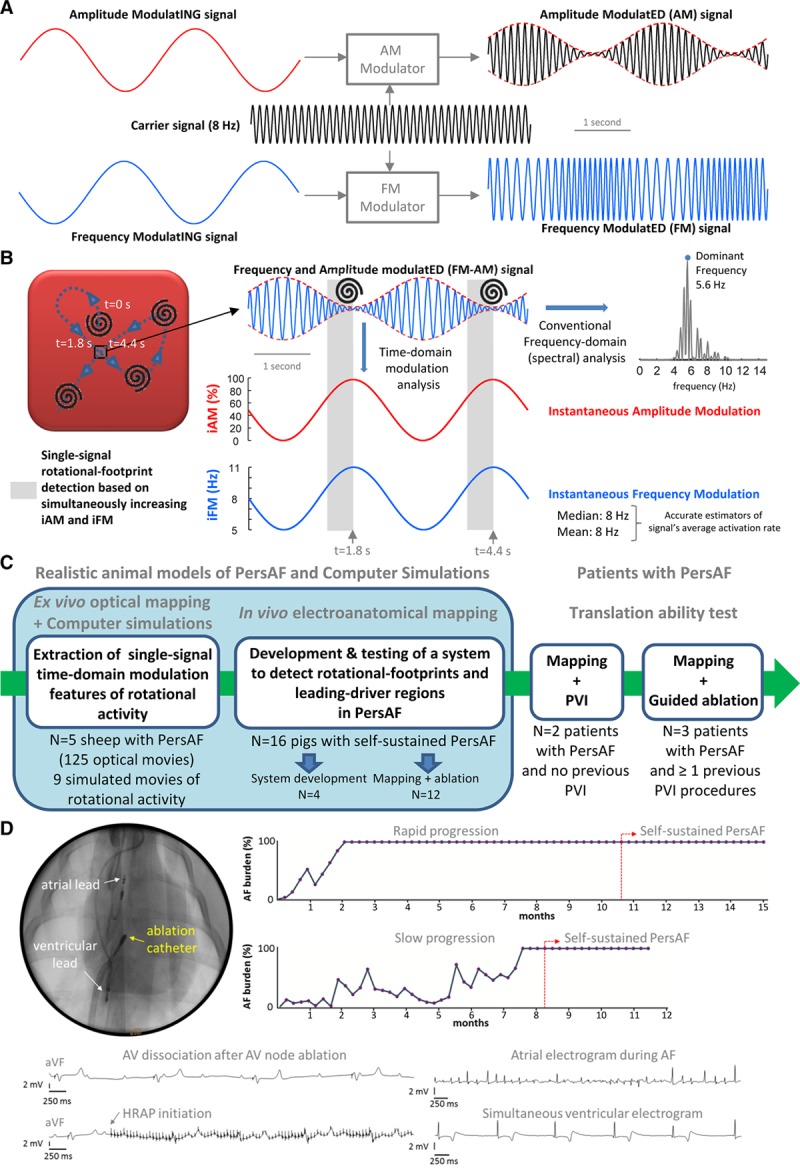
**Amplitude modulation (AM)/frequency modulation (FM) concept and study design.** **A**, AM and FM in radio broadcasting. **B**, **Left**, during cardiac fibrillation, scroll wave/rotor drifting gives rise to AM and FM. When a drifting scroll wave filament/rotor core approaches the square, the amplitude of the action potential decreases increasing instantaneous AM (iAM; in red). Simultaneously, as the wave-emitting source (scroll wave filament/rotor core) approaches, the perceived instantaneous FM (iFM; (*Continued* )**Figure 1 Continued.** in blue) at the spot increases (Doppler Effect). Therefore, simultaneous iAM/iFM increase indicates drifting scroll waves/rotors in the surroundings. Additionally, the areas with the highest average iFM are those potentially driving fibrillation. **Right**, average iFM is estimated from its median and mean values (8 Hz both) and with the conventional dominant frequency (DF) spectral approach (5.6 Hz). Interestingly, the time intervals with the highest iFM usually show the lowest amplitudes and vice versa, which conditions the height of their corresponding power spectral peaks and limits the value of DF-based hierarchical approaches. **C**, Translational approach for the study. **D**, Porcine experimental model of persistent atrial fibrillation (PersAF). AF indicates atrial fibrillation; HRAP, high-rate atrial pacing; and PVI, pulmonary vein isolation.

We hypothesized that (1) the combined analysis of the instantaneous FM (iFM) and instantaneous AM (iAM) of single signals during PersAF can detect the footprint of rotational activity without the need of costly PMEAS; (2) spatiotemporally stable regions with higher average iFM than their surroundings often drive PersAF and their ablation terminates AF and renders it nonsustainable; and (3) rotational activations are sensitive but not specific to such driver regions. To test these hypotheses, we used a completely translational approach (Figure 1C), including computer simulations, optical mapping of isolated Langendorff-perfused PersAF sheep hearts, in vivo electroanatomical mapping of a clinically relevant porcine model of long-term self-sustained PersAF, and complementary studies in patients with PersAF.

## Methods

Full details of the novel analytical methods and algorithms used in this article are fully disclosed within the article and the Online Data Supplement so that they can be implemented in any programming language. The code that implements single-signal calculation of iFM/iAM and single-signal detection of rotational-footprints, as well as the data that support the findings of this study, are available to other researchers from the corresponding author on reasonable request.

All animal procedures were approved by the local Committees on Use and Care of Animals and complied with institutional (University of Michigan, Centro Nacional de Investigaciones Cardiovasculares), National Institutes of Health, and European guidelines. All animal procedures were performed under general anesthesia (see Expanded Methods in the Online Data Supplement). The Hospital Clínico San Carlos ethics committee approved the protocol in patients, and all subjects signed an informed consent.

### Ovine and Porcine Experimental Models of PersAF

The novel iFM/iAM algorithms presented in this study were tested using optical mapping data from 5 PersAF sheep from a previous study^11^ and electroanatomical mapping data from 19 pigs obtained prospectively for this study. PersAF in the sheep was developed using a high-rate atrial pacing (HRAP) protocol^11^ (see Online Data Supplement for details). In pigs, we used slow-growing cross-breeding animals (Yucatan-Large white) to minimize problems arising from handling too large animals after long periods in AF. Unlike sheep, HRAP in pigs would lead to heart failure due to fast atrioventricular conduction. Therefore, we implanted a dual-chamber pacemaker (Accent DR-RF, Abbott) with atrial and ventricular leads that were inserted into the right atrial (RA) appendage and right ventricular apex, respectively. Pigs were 5.2 (interquartile range [IQR], 4.5–6.1)-month-old and weighted 44.0 (IQR, 40.0–45.5) kg at implantation. After 10 (IQR, 10–11) days of recovery, all pigs underwent atrioventricular node ablation before attempting HRAP. More specifically, in 16 out of 19 pigs, the pacemakers were programmed to induce AF by 30-s bursts of HRAP (20 Hz, twice diastolic threshold) followed by a 6-s sensing period. The ventricles were continuously paced using a rate modulation mode between 60 and 110 bpm. On sinus rhythm detection, HRAP automatically resumed until AF became self-sustained and HRAP was no longer necessary. Atrial electrograms were stored using the automatic mode switch algorithm of the pacemakers to generate AF burden curves and follow AF evolution from initial self-terminating episodes to PersAF (episodes lasting >7 days without HRAP, Figure 1D). In 3 out of 19 pigs, the HRAP protocol was never activated and constituted the sham-operated group. We generated the minimum number of sham-operated animals to confirm that persistent AF in pigs also resulted in larger atrial dilation and higher fibrosis content compared with control animals, as previously reported in the sheep model.^22^ All pigs underwent sequential follow-up every 3 weeks, including pacemaker interrogation and transthoracic echocardiography.

### Ex Vivo Optical Mapping of Sheep Hearts With PersAF

Explanted and Langendorff-perfused sheep hearts (animal weight at the time of euthanasia: ≈66 kg) with PersAF were used for epicardial optical mapping of the LA free wall using a voltage-sensitive dye (Di-4-ANEPPS) and an electromechanical uncoupler (Blebbistatin).^11^ Spontaneous AF was allowed to continue uninterruptedly for 50 minutes. Optical movies (5-s duration) were acquired at 2-minute intervals (25 movies per sheep).^11^ See Online Data Supplement for further details.

### Ex Vivo Optical Signal Processing

#### Phase Mapping and Phase Singularity Detection

Phase movies were obtained by means of Hilbert transformation of the optical action potentials. A phase singularity (PS) was defined as the pivoting point where all phases converged during rotational activation.^23^ PS detection was implemented in Matlab using the method previously described by Iyer and Gray.^24^ Briefly, the line integral or change in phase around a pixel is computed using a square path length. If the latter phase difference is equal to ±2π, the path encloses a PS. More details are provided in the Online Data Supplement.

#### Time-Domain iFM/iAM Calculation and Rotational-Footprint Detection

**Figure 2.**
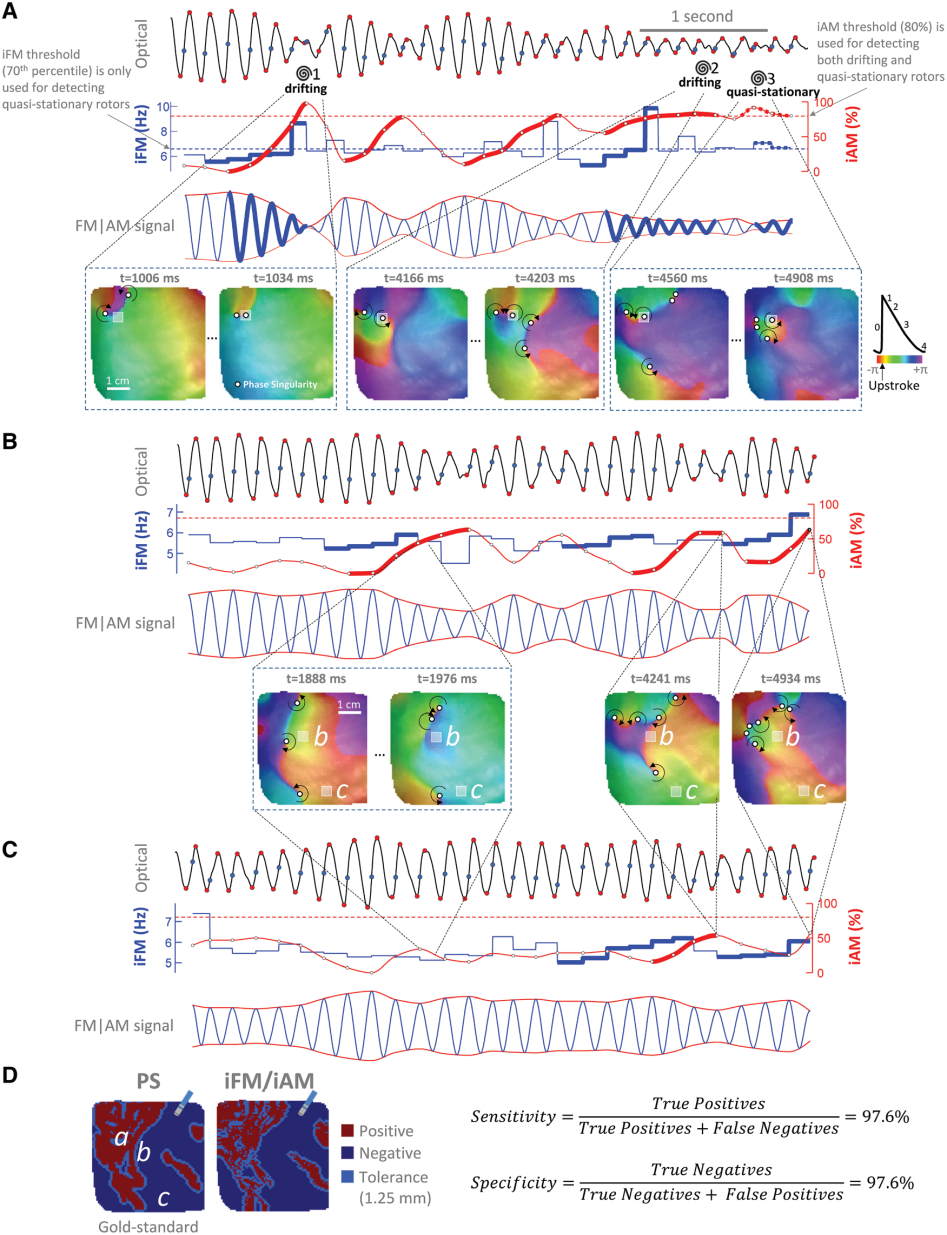
**Examples of instantaneous frequency modulation (iFM)/instantaneous amplitude modulation (iAM) from an optical movie in a persistent atrial fibrillation (PersAF) sheep heart (Online Movie I).** **A**, **Top**, signal from a pixel (gray square in **bottom** maps, enlarged to ease visualization) crossed by a phase singularity (PS) in a figure-of-eight reentry. Spirals mark the times at which a PS (white circles) passes by the pixel. Blue points, activation times. Red points, start and end of phase-0. Activation times and phase-0 amplitude excursions are used to generate the iFM (second row, in blue) and the iAM (second row, in red) signals, respectively. Time intervals with sustained simultaneously increasing iFM (second row, thick blue tracings) and iAM (thick red tracings) reaching a prespecified iAM threshold (horizontal red dashed line) are detected. Afterward, the rotor is still considered to be around while iAM keeps over the threshold regardless of iFM (see rotor no. 2 and Online Figure/Movie II). Time intervals with simultaneously high, but not necessarily increasing, iFM and iAM above prespecified thresholds (horizontal blue and red dashed lines, respectively) are detected as quasistationary rotors (see rotor no. 3). The third row displays a synthetic FM | AM signal in which the rotational-footprint positive intervals are highlighted. (*Continued* )**Figure 2 Continued. B**, Signal from a pixel close to areas swept by drifting rotors but not actually crossed by their associated PS. Note that there are still intervals with simultaneously increasing iFM and iAM. However, iAM does not reach the prespecified threshold. Therefore, the algorithm properly classifies the pixel as rotational-footprint negative. **C**, Signal from a pixel far from areas swept by drifting rotors. Increasing iAM, although still present, is not as noticeable as in pixels very close to or actually crossed by drifting rotors. Therefore, the algorithm also classifies this pixel as rotational-footprint negative. **D**, Comparison between the pixels actually crossed by a PS (PS map, “gold standard”) and the pixels detected by the single-signal algorithm as rotational-footprint positive (iFM/iAM map). The size of a conventional ablation catheter is shown for reference. The signals shown in **A**–**C** were taken from pixels a–c. Additional examples are shown in Online Figure IV.

The following steps were performed for every signal in the optical movie (80×80=6400 signals): (1) detection of activations at the times when phase-0 slopes are maximal; (2) measurement of phase-0 amplitudes; (3) calculation of iFM from the sequence of activation times (the shorter the interval between consecutive activations, the higher the iFM); (4) calculation of iAM from the sequence of phase-0 amplitudes (the lower the amplitude, the higher the iAM). Figure 2A through 2C shows examples obtained from a movie with highly complex AF dynamics (Online Movie I), in which the closer the signal is to a rotor core, the higher the iAM. Moreover, intervals of increasing iFM (Doppler effect) are observed when drifting rotors approach an optical spot.

Therefore, single-signal detection of a rotational-footprint was based on (1) simultaneous increase in iFM and iAM (reaching a certain iAM threshold), which indicated drifting rotors approaching a spot (Figure 2A, rotors 1–2), or (2) simultaneously high, but not necessarily increasing, iAM and iFM values (above certain iAM and iFM thresholds), which indicated quasistationary rotors or rotors meandering around a location (Figure 2A, rotor 3). See Online Data Supplement and Online Figures I through IV for further details.

#### Sensitivity and Specificity of Rotational-Footprint Detection

Locations detected as rotational-footprint positive were compared with the locations with optical PS (pivoting points of rotational activity in optical phase movies). Algorithm sensitivity/specificity was calculated for 3 spatial tolerances: 0, 1.25 (radius of a conventional ablation catheter) and 2.5 mm (diameter of a conventional ablation catheter). An example is shown in Figure 2D. Unlike phase mapping, which requires multiple signals to detect rotations, the iFM/iAM algorithm detects rotational-footprints for each signal independently.

### Computer Simulations

The robustness of iFM calculation and single-signal rotational-footprint detection was also tested using previously published computer simulations of 2-dimensional rotational activity, which incorporated ionic models of sheep and human atrial cells during PersAF. Further testing was performed after including different levels of additive white gaussian noise in the simulated action potentials and unipolar signals (signal-to-noise ratios=∞/30/20/10/0 dB). In addition to baseline conditions for PersAF, the effect of modifying or blocking specific currents was also included to test the algorithm with a wider range of rotational tip meandering behaviors (see Online Data Supplement and Online Figure V for further details).

### In Vivo Electroanatomical Mapping of Pigs With PersAF

Mapping and ablation procedures were guided using Ensite Precision (Abbott). A long steerable introducer (Agilis NxT Steerable, 82 cm; Abbott) was used to properly perform the transeptal puncture and electroanatomical mapping of the LA. A PentaRay catheter (20 poles, Biosense Webster) was connected to the pin box of the electrophysiology recording system using a custom-made adaptor. Then, the catheter was positioned sequentially at multiple locations of the endocardium to reconstruct the RA, coronary sinus (CS), and LA anatomy. Eight-second unipolar signals were acquired at each location. Data from the first 4 pigs with PersAF were used to develop a computational tool for in vivo procedures (development group). Twelve more PersAF pigs (ablation group) also underwent a subsequent second biatrial map to assess spatiotemporal stability of drivers. Finally, data were exported for intraprocedural signal processing.

### In Vivo Electrical Signal Processing

#### Ventricular Far-Field Minimization/Rejection

First, ventricular far-field was subtracted from the unipolar signals using Principal Component Analysis.^11^ Then, a novel second algorithm using surface ECG, unipolar, and bipolar signals, was used to discern whether the resulting negative slope of unipolar signals during ventricular activation were ventricular far-field residues or true atrial deflections (see Online Data Supplement and Online Figures VI and VII).

#### Computation of Time-Domain iFM/iAM: Generation of Leading-Driver Maps and Rotational-Footprint Detection

**Figure 3.**
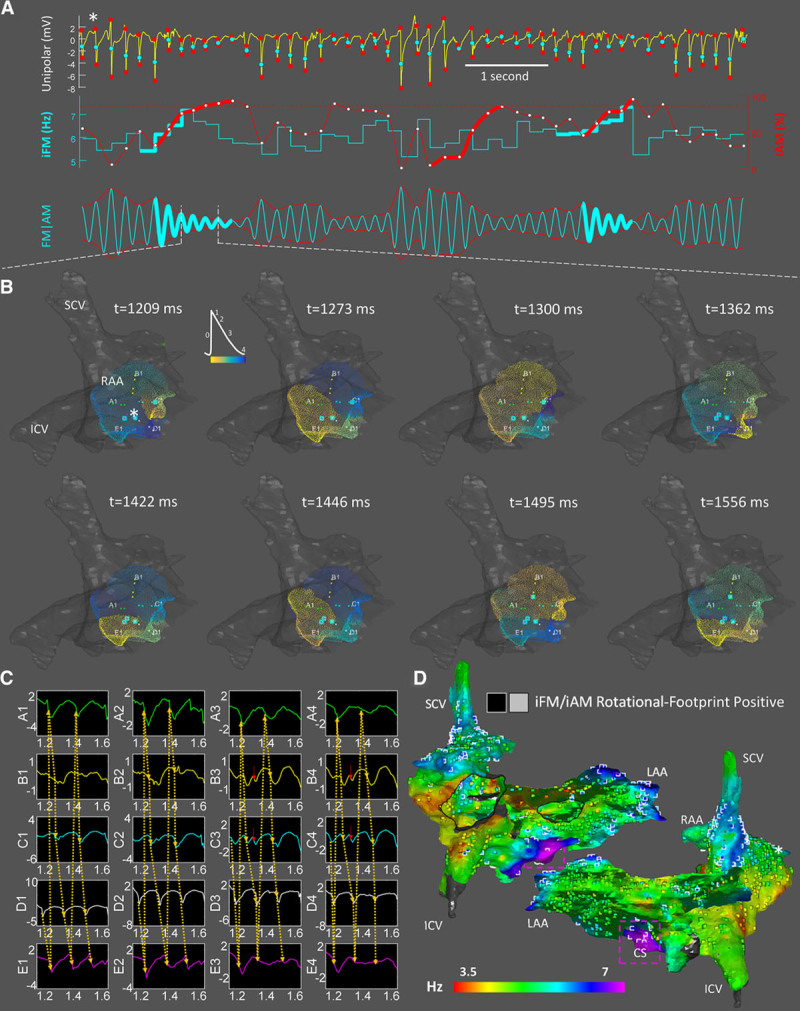
**In vivo** **instantaneous frequency modulation (iFM)/ instantaneous amplitude modulation (iAM) calculation and single-signal rotational-footprint detection from unipolar electrical signals.** **A**, Activation times (first row, cyan points) and unipolar amplitude excursions between the starts and ends of negative deflections (first row, red points) were used to generate the iFM (second row, cyan tracing) and the iAM (second row, red tracing) signals, respectively. Amplitude excursions simultaneous with ventricular activations were interpolated from the surrounding excursions because ventricular far-field residues may affect them, even after applying QRS minimization strategies. Time intervals with sustained simultaneously increasing iFM (thick cyan) and iAM (thick red) reaching a prespecified iAM threshold (dotted horizontal red line) are detected. Afterward, the rotor is still considered to be around while iAM keeps over the threshold and regardless of iFM. The third row displays a synthetic FM | AM signal (morphologically similar to an optical signal) that incorporated both iFM and iAM dynamic changes. Rotational-footprint positive intervals are highlighted. **B**, Snapshots from the phase movie obtained by interpolating data from the 20 electrodes of a PentaRay catheter fully deployed in the right atrial appendage (RAA; Online Movie III). Electrode locations with rotational-footprint positive signals are highlighted with cyan squares. Note the high correlation between highlighted electrodes and the center of rotation in the phase movie. See also Online Movie IV from left atrial appendage (LAA). **C**, Unipolar electrograms confirming the reentrant activation displayed in **B**. Red arrows mark depolarizations that may be explained by precession of the rotational core (Online Figure/Movie II). **D**, Combined leading-driver (iFM_median_) + rotational-footprint map. Rotational-footprint positive locations are marked with black/white squares within light/dark areas, respectively. Interestingly, many regions displayed repetitive rotational activations, including the RAA and LAA. However, most of them did not seem hierarchically relevant to drive AF since it acutely terminated and was rendered nonsustainable after ablating only the purple area located in the coronary sinus. Importantly, rotational-footprints were also found in that region. *The location from which the signal in **A** was retrieved. ICV indicates inferior cava vein; and SCV, superior cava vein.

Activations were detected at the times when the slopes of the unipolar negative deflections were maximal (Figure 3A and Online Figure VIIIA and VIIIB). From these activation times, the iFM signal was generated in the same way as optical signals. Then, iFM_median_ values were calculated for each signal in the electroanatomical map and displayed over the atrial anatomy (Figure 3 and Online Figure VIIIC). Atrial regions with iFM_median_ values clearly higher than their surroundings were considered as potential leading-drivers of PersAF (usually locations with iFM_median_ values within the top 30% in the color scale). They were displayed in iFM_median_ maps as dark colors (navy blue, purple, and magenta). Acquisition points in driver regions were reviewed to ensure that automatic measurements were reliable. Importantly, when individual points presented outlier but reliable values of high iFM_median_, they were specifically tagged and excluded from 3-dimensional interpolation (see Online Data Supplement).

Amplitudes of the unipolar negative deflections (surrogates of the optical phase-0 amplitudes) were calculated around each activation time to compute the iAM signal (the lower the amplitude deflection, the higher the iAM). Single-signal detection of a rotational-footprint was based on the same 2 conditions described for optical signals. A representative sample case of a rotational-footprint positive location based on iFM and iAM is shown in Figure 3A through 3C. Finally, iFM_median_ maps (leading-driver maps) and rotational-footprint positive locations were presented together to easily visualize their spatial correlation (Figure 3D). See Online Data Supplement and Online Figures VIII through XIV for further details.

### Ablation Protocol of Potential Leading-Driver Regions

An open-irrigated tip catheter (FlexAbility, Abbott) was used to deliver radiofrequency energy (30 W at the posterior LA, 35 W at other atrial regions, temperature control mode at 41°C, saline irrigation: 17 mL/min) on potential leading-driver locations until the following end points were reached: (1) conversion to sinus rhythm or ablation of all leading-driver locations; and (2) nonsustainability of AF upon reinduction. Radiofrequency was delivered until local potentials were completely abated through the creation of coin-like sets of lesions.^25^ When targeting the CS, radiofrequency was first delivered from the corresponding adjacent area of the LA. If AF persisted, radiofrequency was also delivered from the CS while decreasing the radiofrequency energy to 25 W to prevent excessive heating of the adjacent ventricular tissue, which can potentially induce VF in pigs. In case of sinus rhythm conversion during the ablation protocol, we performed ≥3 AF reinductions via 30-s HRAP. AF was considered nonsustainable when persisting <10 minutes in all reinduced episodes. Otherwise, the ablation protocol was resumed until targeting all leading-driver locations or AF was rendered nonsustainable. Radiofrequency times to AF termination and for nonsustainability were automatically annotated on the LabSystem-Pro recording system (Boston Scientific). If ablation did not terminate AF, the atrial frequency content of the 12-lead surface ECG (see Online Figures XV and XVI) was compared before and after ablation to discern whether ablation effectively modified the AF substrate.

### Histopathology Analysis

Pigs were euthanized with pentobarbital and the heart was removed via median sternotomy. Myocardial tissue samples from nonablated regions in the RA and LA free walls and in the posterior LA were fixed in 4% formalin for at least 14 days. Afterward, cross-sections of the myocardium were dehydrated, embedded in paraffin, and cut into 5-µm thick sections. Tissue slices were stained with PricoSirius Red and digitized using a NanoZoomer S360 Digital slide scanner (Hamamatsu, Japan) for analysis. A total of 10 randomly selected 20X insets per slide were analyzed (excluding endocardial, epicardial, and peri-vascular regions). Interstitial fibrosis was quantified using ImageJ with a color deconvolution-based plugging. Interstitial fibrosis proportion (red stained areas/total area) was measured in every inset, and a mean value was assigned to each sample.

### Electroanatomical Mapping and Ablation in Patients

Electroanatomical mapping was performed in a similar manner to the pig model (see Online Data Supplement). We only acquired 1 biatrial map to avoid unnecessary prolongation of the procedure. The mapping approach was first tested in 2 patients undergoing PVI, without any previous ablation (observational group). The next 3 patients were in PersAF despite ≥1 previous PVI procedures, and the ablation targets were identified using the iFM/iAM maps (interventional group). The absence of electrical activity inside the PVs was used to confirm PVI. In the interventional group, the physician in charge could stop ablation if leading-driver regions were considered too large to be ablated using a catheter-based approach. Any atrial tachycardia/flutter occurring after AF termination/reinduction were also mapped and ablated. If AF persisted after stopping ablation, electrical cardioversion was performed, and the procedure terminated after further confirmation of PVI. All patients underwent clinical follow-up to assess recurrences.

### Statistical Analyses

Data are presented as median (interquartile range), except where noted. The Wilcoxon signed-rank test was used for paired measurements. The Mann-Whitney test was used for comparisons between 2 groups of nonpaired data. All analyses were performed using GraphPad Prism 6. Mean (SD) is provided for data with a high n value and normal distribution according to the Shapiro-Wilk test.

## Results

### iFM/iAM Algorithm Successfully Detected Rotational-Footprints Using Single Signals

**Figure 4.**
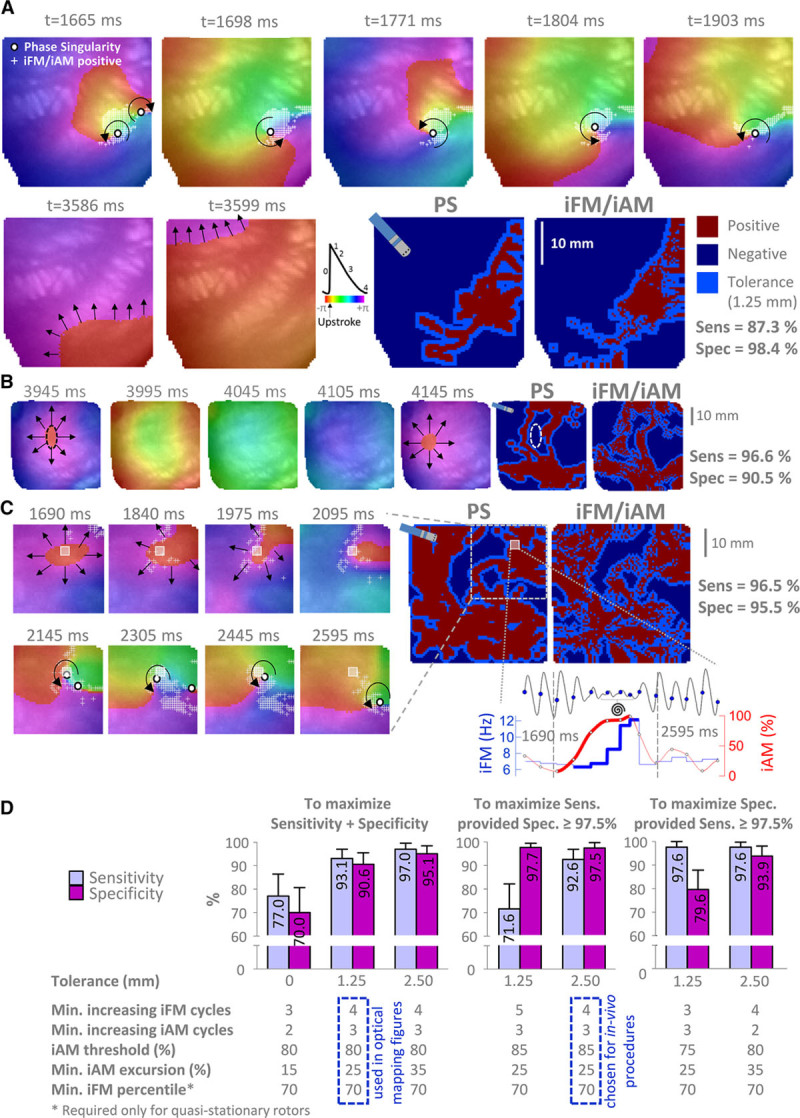
**Performance of the single-signal instantaneous frequency modulation (iFM)/ instantaneous amplitude modulation (iAM) algorithm to detect rotational-footprints in optical movies from sheep with persistent atrial fibrillation.** **A**, Snapshots from sheep no. 1 (Online Movie V) with a drifting rotor (**top** row) that eventually leaves the field of view. Then, planar wavefronts are observed. The single-signal algorithm yielded positive results (white +) in the pixels near the pivoting point of the drifting rotor (phase singularity [PS]), but no pixel was tagged positive during planar wavefront intervals. Considering a 1.25 mm tolerance (light blue areas, width equal to the radius of an ablation electrode), both PS and iFM/iAM maps were extremely similar. **B**, Snapshots from sheep no. 2 (Online Movie VI) displaying an interval with centrifugal activation. **C**, Snapshots from sheep no. 3 (Online Movie VII) displaying an interval (1690–2595 ms) with breakthrough activations. The signal from the pixel marked with a gray square is shown during the same time interval, when a simultaneous increase in iFM and iAM is present. Therefore, the initial breakthroughs might be the result of a scroll wave with a changing filament approaching the mapped surface. Indeed, this breakthrough activation eventually turned into a drifting figure-of-eight reentry. **D**, Summary of sensitivity (sens) and specificity (spec) using the optimal parameter combinations for the iFM/iAM algorithm (n=117 optical mapping movies). Data displayed normal distribution (Shapiro-Wilk test) and are shown as mean and SD.

Almost 700 000 optical signals from 117 high-quality optical movies from 5 sheep with PersAF were analyzed (8 movies were excluded because of suboptimal quality). The algorithm automatically classified as rotational-footprint positive the locations near the cores of drifting rotors and figure-of-eight reentries, as well as breakthrough activations that eventually turned into figure-of-eight reentries. Conversely, the algorithm detected rotational-footprint negative pixels when true focal or planar wavefront activations were present. Examples of rotational-footprint positive and PS positive maps (used as “gold standard”) from the 5 sheep are shown in Figure 4A through 4C, Online Figure XVIIA and XVIIB and Online Movies V through IX. Sensitivity and specificity of the iFM/iAM algorithm were tested for 8820 different combinations of 5 parameters: minimum number of increasing iFM cycles, minimum number of increasing iAM cycles, iAM threshold, minimum iAM excursion, and minimum iFM percentile. This was repeated applying 2 different minimum refractory periods (RP_min_) to detect activations (either constant or signal-specific, see Online Data Supplement for details), for 3 levels of spatial tolerance (0/1.25/2.5 mm) and requiring that all criteria were simultaneously met for several consecutive cycles between 2 and 5. That resulted in a total of 30 958 200 data registers (Online Table I). Importantly, the use of a signal-specific RP_min_ provided higher specificity values. Optimal parameter combinations for any number of parameters (from 1 to 5) are presented in Online Table II. A summary is presented in Figure 4D and Online Table III. In the aforementioned Figures/Movies, we used the parameter combination that maximized the sum of sensitivity and specificity for a tolerance of 1.25 mm (minimum number of increasing iFM cycles=4, minimum number of increasing iAM cycles=3, iAM threshold=80%, minimum iAM excursion=25%, and minimum iFM percentile=70th, [sensitivity: 93.1 (3.9)%; specificity: 90.6 (4.9)%]).

A similar analysis was repeated using synthetic transmembrane action potentials and unipolar signals from 9 simulations of quasistationary rotors in computer models of sheep and human PersAF. Such an analysis suggested that slightly different parameter settings than those obtained from optical mapping data may be more sensitive for detecting the rotational footprint of quasistationary rotors. Thus, using a fixed RP_min_ of 50 ms (instead of a signal-specific one, Online Table IV) and 3 (instead of 4) as the minimum number of increasing iFM cycles (Online Table V) improved the algorithm sensitivity to quasistationary rotors in computer simulations devoid of noise. However, the latter changes in the RP_min_ or parameter settings would be at the cost of importantly decreasing the algorithm’s specificity when used with experimental data (Online Tables VI and III and Online Figure XVIII), where noise is present and drifting rotors were much more prevalent than quasistationary ones. Online Table VII shows the performance of the iFM/iAM algorithm for single-signal detection of the rotational-footprint of quasistationary rotors in computer simulations in the presence of varying levels of additive white gaussian noise. When using unipolar signals, the algorithm performed well in low noise environments (signal-to-noise ratio, 30/20 dB) with both constant and signal-specific RP_min_. However, specificity collapsed in a noisy scenario (signal-to-noise ratio, 10 dB) if a fixed 50 ms RP_min_ was established, but remained stable using a signal-specific RP_min_. As expected, the single-signal rotational-footprint detection algorithm was rendered useless when noise was as powerful as unipolar signals (signal-to-noise ratio, 0 dB). But even then, a signal-specific RP_min_ achieved much better specificity values than a fixed one. In addition, a minimum number of increasing iFM cycles=4 usually performed better than 3 in the presence of non-negligible noise. Importantly, the use of a signal-specific RP_min_ enabled the accurate measurement of iFM_median_ with unipolar signals (key for appropriately locating leading-driver regions), even in highly noisy simulated scenarios (Online Table VIII and Online Figure XIX).

Therefore, for the in vivo experiments, we chose to stick to a signal-specific RP_min_ that displayed great robustness in the presence of noise, and to experimentally derived parameters that offered high enough sensitivities for detecting quasistationary rotors with a 2.5 mm tolerance using unipolar signals in computer simulations (Online Table IV) and at the same time very high specificity and sensitivity when used with experimental data (Online Table III, Figure 4D and Online Figure XVIII). Finally, to further increase specificity for the in vivo experiments, we chose the combination that maximized sensitivity as long as specificity was ≥97.5% with a 2.5 mm tolerance (smaller than the diameter of conventional ablation catheters and lesions). This was achieved by increasing the iAM threshold from 80% to 85%, which provided 92.6 (4.3)% sensitivity and 97.5 (2.3)% specificity (Figure 4D).

### Pigs Developed Self-Sustained PersAF for Several Months Before In Vivo Procedures

**Figure 5.**
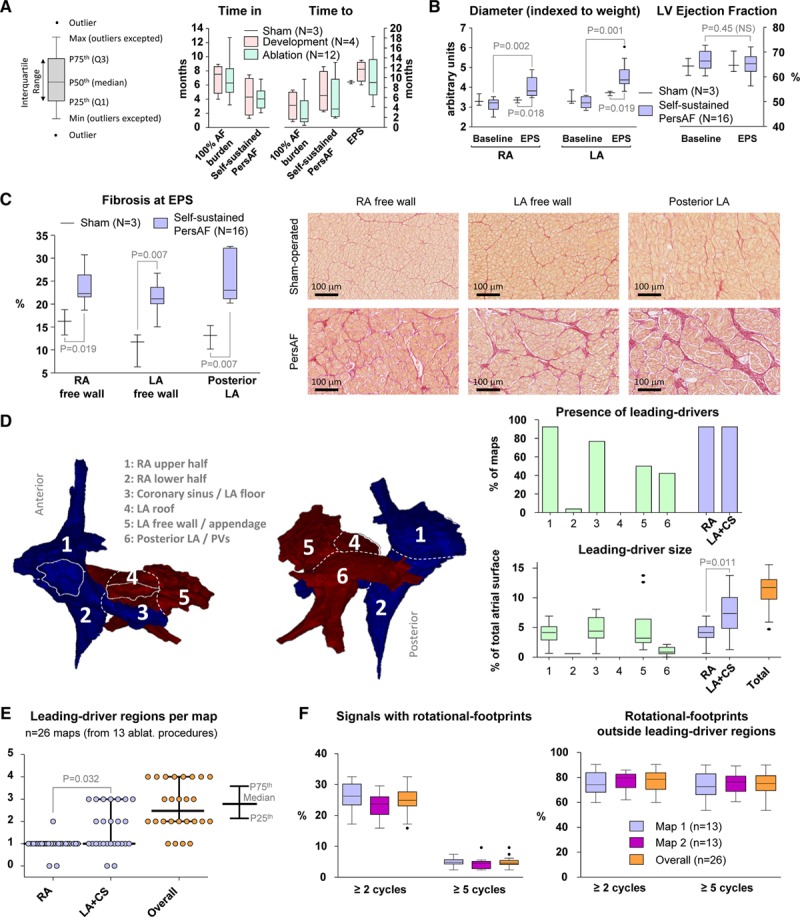
**In vivo** **quantitative results of the porcine model of self-sustained persistent atrial fibrillation (PersAF).** **A**, Protocol times since high-rate atrial pacing initiation. **B**, Atrial diameters and left ventricular (LV) ejection fractions measured with echocardiography. The Wilcoxon signed-rank test was used to compare paired measurements from the same pigs at baseline and at the time of the electrophysiological study (EPS). The Mann-Whitney test was used for comparisons between sham-operated and self-sustained PersAF animals. **C**, Increased atrial fibrosis was found in self-sustained PersAF hearts (Mann-Whitney test). **D**, Anatomic distribution and size of leading-driver regions. The cumulative size (%) of the leading-driver regions found in the left atrium (LA) + coronary sinus (CS) was higher than the cumulative size of those in the right atrium (RA; Wilcoxon signed-rank test). **E**, The number of leading-driver regions was significantly higher in the LA+CS than in the RA (Wilcoxon signed-rank test). **F**, Rotational-footprint quantification and spatial correlation with leading-driver regions. The percentage of signals with rotational-footprints and the percentage of rotational-footprints outside leading-driver regions did not show significant differences between the first and second maps (Wilcoxon signed-rank test). Importantly, around 3-quarters of all rotational-footprints were found outside leading-driver regions. PV indicates pulmonary vein.

All pigs in both the development and the ablation groups (n=16) developed self-sustained PersAF after 4.4 (IQR, 2.5–9.9) months of HRAP. In vivo mapping procedures were conducted after 4.1 (IQR, 2.7–5.4) months of self-sustained PersAF and after a 7.2 (IQR, 5.0–8.3)-month period with 100% AF burden (weight at the electroanatomical mapping procedure: 96 [IQR, 83–108] kg, Online Table IX). Figure 5A shows protocol times for the pigs in both development and ablation groups separately. As AF developed, echocardiography data showed overt atrial dilation. Conversely, left ventricular ejection fraction remained normal (Figure 5B). PersAF pigs showed considerably higher levels of fibrosis in RA and LA free walls and posterior LA than sham-operated pigs (Figure 5C). The electroanatomical mapping data from the first 4 out of 16 pigs with self-sustained PersAF (development group) was used to adapt and test the ex vivo iFM/iAM optical signal processing for its use with in vivo unipolar electrical signals.

### iFM_median_ Maps Revealed Stable PersAF Leading-Driver Regions

The remaining 12 self-sustained PersAF pigs (ablation group) underwent 14 in vivo high-density mapping procedures (2 pigs were mapped several months apart) with 2 biatrial maps per procedure. A total of 13 ablation procedures were performed (one pig underwent 2 ablation procedures).

A total of 4920 (IQR, 4435–5855) signals were acquired per biatrial map, which required 92 (IQR, 82–98) minutes (Online Figure XX). First and second maps were finished 2.6 (IQR, 2.4–2.9) hours apart. Before the 13 ablation procedures, a total of 34 and 35 leading-driver regions were prospectively identified in the initial maps and remaps, respectively. Importantly, 95.7% of such leading-driver regions exactly colocalized in the 2 maps. Anatomically, leading-driver regions were located at the upper half of the RA (92.3% of maps), CS/LA floor (76.9% of maps), LA free wall/appendage (50.0% of maps) and posterior LA/PVs (42.3% of maps). All leading-driver regions together covered 11.7% (IQR, 9.8%–13.1%) of the total atrial surface (Figure 5D and Online Figure XXI). The total number of leading-driver regions per biatrial map was 2.5 (IQR, 2.0–4.0; Figure 5E). Maximum and mean values of iFM_median_ inside leading-driver regions were 7.9 (IQR, 7.0–9.3) Hz and 7.3 (IQR, 6.5–8.1) Hz, respectively. The corresponding gradients with their surroundings were 2.7 (IQR, 2.2–3.3) Hz and 1.8 (IQR, 1.5–2.1) Hz, respectively. Neither maximum/mean values nor iFM_median_ gradients displayed significant differences among regions (Online Figure XXII).

**Figure 6.**
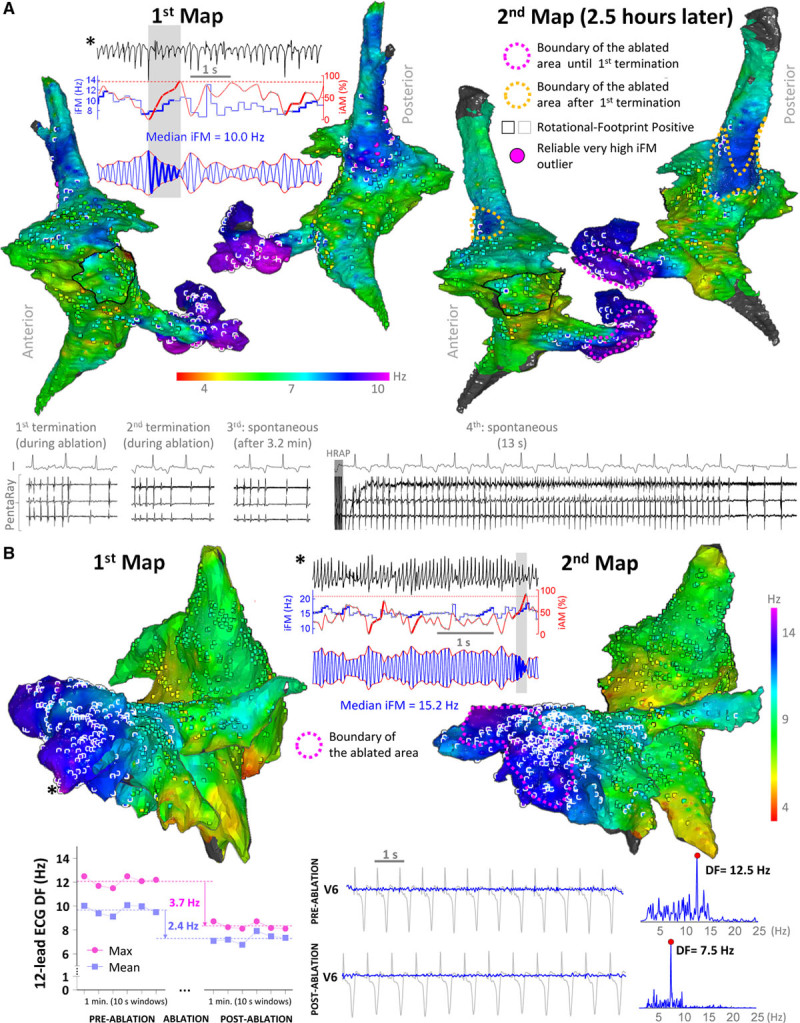
**Leading-driver regions are highly stable intraprocedure**. **A**, Example of persistent atrial fibrillation (AF) termination after ablating the coronary sinus region delineated with a fuchsia dashed line for 11.4 min. Then, AF was reinduced and lasted >10 min. After 11 additional minutes of radiofrequency delivery (orange dashed line), AF terminated again and was no longer sustainable. **B**, Case with extensive atrial remodeling due to severe tricuspid regurgitation. Much higher median values of instantaneous frequency modulation (iFM_median_) than in the rest of animals were documented over large areas of the left atrium. AF did not terminate despite 97 min of radiofrequency delivery. However, ablation resulted in an important reduction in the overall atrial activation rate measured by dominant frequency (DF) from the 12-lead surface ECG (**bottom** panel), which indicated that ablation effectively modified the AF substrate. *The locations where the displayed signals were retrieved from. iAM indicates instantaneous amplitude modulation; and iFM, instantaneous frequency modulation.

Only regions that were prospectively identified as leading-drivers were targeted. AF terminated during ablation of leading-driver regions in 12 out of the 13 (92.3%) ablation procedures after 16.9 (IQR, 9.2–35.8) minutes of radiofrequency delivery. Once terminated, sustained AF (≥10 minutes) could not be induced in one-third of the procedures. In the remaining two-thirds, the ablation protocol was resumed after an induced episode exceeded 10 minutes. Finally, after 20.4 (IQR, 12.8–44.0) minutes of total radiofrequency delivery on 2 (IQR, 1.25–2.75) leading-driver regions, AF was no longer sustainable. Specifically, 26 out of the 33 leading-driver regions identified on the initial maps had to be targeted to achieve nonsustainability of AF in the 12 successful ablation procedures (11.8 [IQR, 6.3–18.1] min/region). Ablation of leading-driver regions did not induce VF. Figure 6A displays an example of intraprocedure stability of iFM maps and PersAF termination after the ablation of leading-driver regions. Conversely, Figure 6B shows data from the only procedure in which ablation did not terminate PersAF.

**Figure 7.**
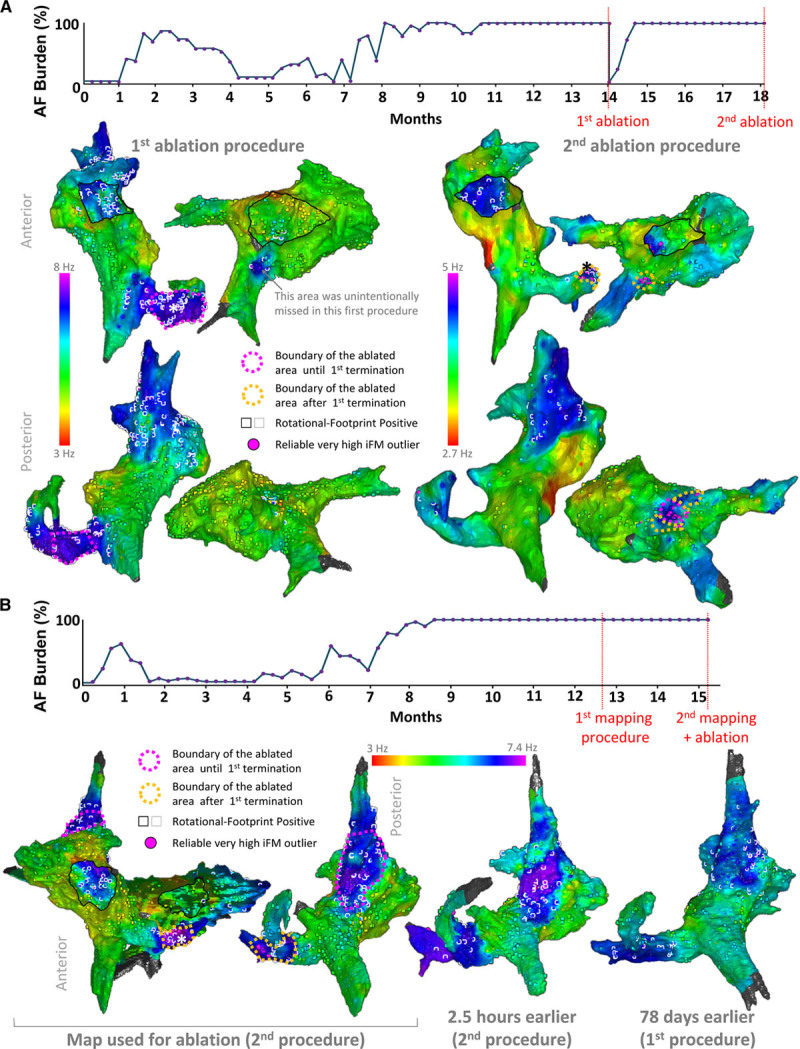
**Leading-driver regions seem stable in the long-term.** **A**, One pig underwent a successful first ablation procedure after which atrial fibrillation (AF) burden was temporally reduced to 0%. Driver areas not ablated in the first procedure remained as key regions in a second procedure >4 mo after resuming high-rate atrial pacing, and >3 mo after reaching self-sustained persistent AF again. Also, a new region with consistent high median values of instantaneous frequency modulation (iFM_median_) outliers was found in the posterior left atrium. After a total radiofrequency delivery of 19.8 min, AF was rendered nonsustainable. **B**, One pig underwent 2 mapping procedures 78 d apart. Note that leading-driver regions, although less electrically remodeled in the first mapping procedure (lower iFM_median_ values), were roughly similar. Online Figures XXIII and XXIV show signals from the marked locations (*).

Figure 7A and Online Figure XXIII show results from a pig that underwent 2 ablation procedures. Importantly, after ablating leading-driver regions in the first procedure, AF burden dramatically dropped from 100% to 0%. Then, it took 3 and 4 weeks of HRAP to reach 100% AF burden and PersAF, respectively. The latter confirmed that ablation of leading-driver regions successfully modified the substrate underlying PersAF maintenance. Another pig also underwent 2 mapping procedures 78 days apart to test the long-term stability of leading-driver regions. Figure 7B and Online Figure XXIV show that similar regions were potentially driving PersAF in both procedures. The driving role of these areas was confirmed in the second procedure that included their ablation whereupon PersAF terminated and was no longer sustained on reinduction (longest reinduced episode: 17 s).

### iFM/iAM Rotational-Footprints Were Present in Every Leading-Driver Region but Mostly Outside Them

Rotational-footprints were detected by the single-signal iFM/iAM algorithm in 24.9% (IQR, 23.1%–27.6%) of all atrial signals. Importantly, despite the inside or the border of 69 out of 69 leading-driver regions displayed rotational-footprints, this only constituted around a quarter of total rotational-footprint positive locations. Thus, the majority of rotational-footprints (76.8% [IQR, 70.5%–83.6%]) were found outside leading-driver regions. When restricting the criteria for rotational-footprint detection at ≥5 consecutive cycles, only 4.6% (IQR, 4.2%–5.4%) of all atrial signals were rotational-footprint positive. Similarly, the inside or the border of 67 out of 69 (97.1%) leading-driver regions showed rotational-footprints. However, the majority of ≥5 consecutive cycle rotational-footprints were found again outside leading-driver regions (Figure 5F). This suggests that regardless of the temporal stability required to identify rotational activations, only a minority of them (those with the highest average iFM values) potentially contributed to PersAF maintenance.

### iFM_median_ Maps Located Leading-Driver Regions in Patients With PersAF

Online Table X shows baseline characteristics and mapping/ablation results of all patients. In the 2 patients from the observational group, iFM_median_ maps were acquired before PVI (Online Figure XXV). The first patient showed a prominent leading-driver region overlapping the ostium of the left superior PV (therefore, potentially targeted using a PVI strategy). The second patient displayed several leading-driver regions outside the PVs. Interestingly, after 16 months of follow-up, the first patient remained free of AF without antiarrhythmic drugs. Conversely, AF recurrences were documented in the second patient despite antiarrhythmic drug therapy during the follow-up.

**Figure 8.**
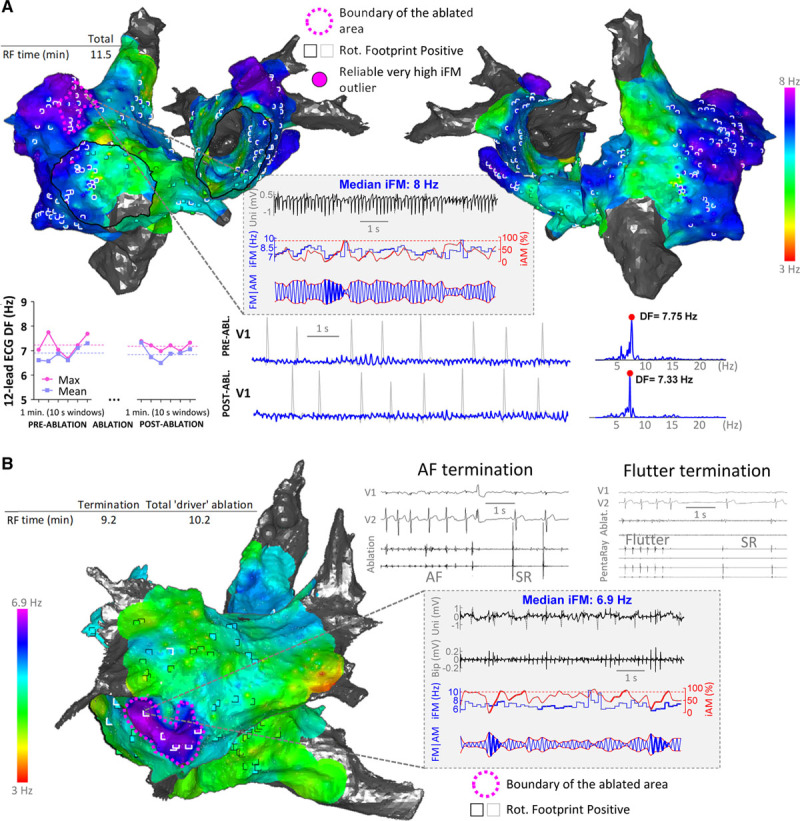
**Translation ability of the instantaneous frequency modulation (iFM)/instantaneous amplitude modulation (iAM) approach to patients with persistent atrial fibrillation (PersAF) despite ≥1 previous pulmonary vein isolation procedures.** **A**, Map from a patient undergoing a third AF ablation procedure. Fast and large leading-driver regions covered a considerable portion of the atria and precluded a limited ablation strategy (fuchsia dashed line) from acutely terminating PersAF. Moreover, radiofrequency delivery did not modify atrial activation rates on the 12-lead ECG. **B**, Map from a patient with a well-delimited leading-driver region. Targeting that region with radiofrequency delivery for ≈10 min successfully terminated PersAF. Upon reinduction, common atrial flutter was the only inducible arrhythmia, which was eventually terminated by creating a linear lesion at the cavotricuspid isthmus. Abl indicates ablation; DF, dominant frequency; RF, radiofrequency; Rot, rotational; and SR, sinus rhythm.

In the interventional group, the only sites targeted with ablation (other than a reconnected PV in patient number 3, see Online Table X) were the leading-driver regions prospectively identified by the algorithm. Indeed, all identified leading-driver regions were targeted in patients number 2 and number 3. However, in patient number 1 (36-year-old, 17-month PersAF history and an on-going 12-month PersAF episode), the algorithm identified large and fast leading-driver regions in both atria, with the fastest locations in both appendages (Figures 8A and Online Figure XXVIA). The physician in charge considered these areas too large for safe and successful ablation with a catheter-based approach. Therefore, the ablation protocol was stopped after 11.5 minutes of RF delivery, and the patient was cardioverted. However, AF recurred after a few days. Interestingly, this kind of maps may anticipate failure of a limited catheter-based ablation strategy. The patient also declined a surgical ablation approach, which could have potentially isolated such regions. Conversely, the map in Figures 8B and Online Figure XXVIB show a well-defined leading-driver region in the LA (outside the PVs) of a patient with a 5-month PersAF episode (similar to the median episode duration in pigs). In this patient, limited ablation (≈10 minutes) successfully terminated PersAF (patient number 2, Online Table X). After 16 months of follow-up, 2 out of the 3 patients in the interventional group remained in sinus rhythm without antiarrhythmic drugs.

## Discussion

The main findings of this study are (1) the combined analysis of the iFM and iAM present in single signals during PersAF can detect the footprint of rotational activity with high sensitivity and specificity and without the need of costly PMEAS; (2) atrial regions with reproducibly higher than surrounding average iFM are often responsible for maintenance of PersAF, are spatiotemporally stable, and their ablation successfully terminates PersAF and renders it nonsustainable upon acute reinduction; and (3) rotational activations are sensitive but not specific to these leading-driver regions.

### Value of Animal Models of PersAF

Our findings have been obtained in realistic and clinically relevant animal models of PersAF. Importantly, the majority of animals showed fibrillatory frequencies very similar to those observed in patients. In pigs and sheep, PersAF was self-sustained over long time periods in the absence of other comorbidities that may be present in patients. The latter has enabled us to test our hypotheses without any confounding factors. However, other clinically relevant models are warranted for future studies.

Results from clinical studies usually include patients that, although classified as PersAF, can be in sinus rhythm at the beginning of the procedure.^7,9^ Therefore, AF needs to be initiated by pacing maneuvers, and radiofrequency ablation may lead to serendipitous AF termination. Conversely, all pigs with PersAF underwent a comprehensive follow-up, which guaranteed that AF was self-sustained for several months before mapping and ablation. This makes serendipitous termination of PersAF much less likely.

Intrinsic limitations of translational animal models of PersAF are mainly related to experimental costs and long follow-up periods before generating suitable specimens for mapping. The latter justifies smaller sample sizes than in most clinical studies. The studies also require a high level of experimental expertise by the investigators involved. However, the procedures are not restricted by the intrinsic temporal limitations of clinical procedures, which have precluded human studies to conclusively test the medium-term (hours) spatiotemporal stability of PersAF drivers. Therefore, we surmise that testing new ablation approaches in realistic animal models of PersAF should be encouraged, especially when so many conflicting clinical outcomes are being reported.^16^

### Mapping Approaches to Detect Rotational Activation and Drivers

Conflicting clinical outcomes may be due in part to technical issues inherent to the various mapping techniques. Thus, activation/phase mapping of electrical data requires multiple electrodes, is completely dependent on their location, contact, separation, and filtering, and has low specificity for rotor detection.^13–15^ Our iFM/iAM algorithm enables in vivo single-signal detection of rotational-footprint locations without the need of multiple electrograms simultaneously acquired by costly PMEAS^7,8^ or fully deployed multispline catheters.^26^ Indeed, electrical phase movies using multispline catheters may be unreliable when catheters are not well deployed or good tissue contact is not ensured.^14^ Previous attempts to search rotational activations by sequential positioning of a fully deployed PentaRay catheter reported coverage of ≈65% of the endocardial atrial surface,^26^ and even there some issues can arise due to interpolation of sparse data. Therefore, activation/phase mapping of electrical data cannot be considered a “gold standard.” Thus, we only used electrical phase movies to qualitatively assess the correlation between the rotational cores in such movies and the rotational-footprint positive locations detected by the iFM/iAM algorithm within areas where the PentaRay catheter was fully deployed (see Figure 3B and Online Movies III and IV). Proper validation of the iFM/iAM algorithm to detect rotational-footprints was performed using high-quality optical mapping movies, which are considered the “gold standard,” and computer simulations. Note that the aforementioned technical limitations of phase mapping do not apply to our optical movies because of their high spatiotemporal resolution (6400 signals separated ≈0.43–0.62 mm).

Importantly, the number of rotations as the only criterion for driver stability may be too simple because drivers can often be only intermittently seen in the mapped surface and yet be critical.^27^ Conversely, the iFM/iAM algorithm detected the time intervals in which increasing iAM and iFM were simultaneously present. It usually anticipated the onset of rotations on the mapped surface (see Online Movies V through IX), which might be due to the presence of underlying intramural scroll waves with changing filament morphology. According to our results, it is also important to establish an activation frequency criterion to discern which reentrant/focal potential drivers are truly relevant for AF maintenance. For this purpose, bipolar dominant frequency (DF) maps have been used to guide ablation in previous approaches that reported suboptimal results in PersAF.^25^ However, the approach used in the present study (iFM/iAM maps) significantly differ from DF maps. Unlike DF, iFM tracks dynamic changes in the local activation rate during acquisition. Therefore, iFM enables the detection of intervals with rotational-footprint or high-frequency short bursts that can be easily missed by DF analysis. Moreover, morphology and iAM/iFM content of electrical signals often result in multiple spectral peaks with similar heights, which makes DF analysis challenging.^28^ The latter might have precluded higher success rates using DF maps to guide PersAF ablation.^25^ Furthermore, we relied on unipolar signals from 1-mm electrodes after careful ventricular far-field minimization/rejection, instead of bipolar signals whose amplitudes depend on wavefront orientation, are not directly proportional to those in the underlying action potentials, and are more prone to fractionation (see Online Data Supplement and Online Figures XXVII and XXVIII for further technical details).

### Spatiotemporal Stability and Potential Underlying Mechanisms of Leading-Driver Regions

We found an extraordinary intracase reproducibility (95.7%) in the location of stable leading-driver regions between maps acquired several hours or even months apart, which supports previous clinical mapping results.^29^ PersAF termination and nonsustainability after ablating stable leading-driver regions confirmed that a small number of localized regions jointly contribute to maintain the arrhythmia in our porcine model. Despite previous studies using contact^18^ and noncontact^17,19^ AF maps have reported only short-term (s or min) spatiotemporal stability of drivers with controversial results, medium/long-term driver stability has usually been assumed as the underlying rationale for patient-tailored ablation approaches. However, to our knowledge, such stability had not been conclusively validated using detailed high-density in vivo contact maps acquired several hours apart. This might be because previous mechanistic approaches have been directly tested in patients,^7–9,25,26^ and ethical concerns prevented increasing procedure duration.

Interestingly, the majority of patient-tailored ablation approaches do not consider the activation frequency dynamics of the targets, but only their focal/rotational nature.^7,8^ In our opinion, the latter might be justified in some scenarios (eg, patients with ≥1-year PersAF in whom frequency gradients among regions may decrease considerably^26^), but not in others. Our results show that not every region with foci/rotors/spatiotemporal dispersion should be targeted and that energy delivery should be focused on stable leading-driver locations. Importantly, half of leading-driver locations were found in the CS/LA floor or LA appendage/free wall. However, PMEAS using basket catheters^8^ or ECG imaging^7^ may not be optimally suited to map those regions. Around one-third of leading-driver regions were located on the posterior RA/superior vena cava junction. Those locations are consistent with some approaches that routinely isolate the LA appendage^30^ or ablate triggers that can (re)initiate AF from those regions.^31^

There is no controversy about the role of focal triggers in (re)initiating AF. However, the fact that PersAF episodes can be usually terminated (at least transiently) and not only reset by electrical cardioversion does not support a purely focal, nonreentrant mechanism maintaining AF.^5^ Some so-called foci might actually represent breakthroughs generated by intramural scroll waves with nonlinear filament shapes (Figure 4C). Conversely, some short-lived rotational-footprints may be a consequence of wave collisions initiating transient rotational activity. Although the aim of this study was not to conclusively discern mechanisms underlying stable leading-driver regions, the presence of rotational-footprints for ≥5 consecutive cycles in >97% of stable leading-driver regions argues in favor of scroll waves/rotors or microanatomic intramural reentry^32^ as the main underlying mechanisms for long-term maintenance of PersAF in the pig model. Worthy of note is that around 3-quarters of rotational-footprints were found outside stable leading-driver regions. The latter suggests that approaches ablating rotational/focal activation^7,8^ or spatiotemporal dispersion^9^ regions regardless of their iFM may be highly sensitive but have low specificity in identifying relevant driver regions. Indeed, we have seen a plethora of rotors undergoing several rotations and appearing repeatedly in regions that did not seem relevant in maintaining PersAF (eg, Online Movies III and IV).

### Comparison of Our Acute Success Rates With Other Mechanistic, Patient-Tailored Strategies

Previous patient-tailored ablation approaches in PersAF have targeted sites with high DF,^25^ rotational and centrifugal activation detected with PMEAS and propriety algorithms,^7,8^ visually detected spatiotemporal dispersion,^9^ and rotor domains detected with a PentaRay catheter and phase mapping.^26^ AF termination rates during driver ablation ranged between 15% (long-standing PersAF)^26^ and 95% (including 23% of patients with paroxysmal AF),^9^ although most studies reported rates from 36%^25^ to 63%.^7^ Average driver radiofrequency times until first AF termination ranged from 18 to 35 minutes,^8,9^ although these data widely varied depending on AF duration.^7^ Seitz et al^9^ also reported noninducibility in 42% of patients after 49 minutes of radiofrequency delivery. In our study, PersAF terminated in 92.3% of pig procedures after a median of 16.9 minutes and rendered nonsustainable after 20.4 minutes of radiofrequency delivery on regions of higher than surrounding iFM. Therefore, despite potential limitations of pig data for comparison with human procedures, the presented approach may achieve acute termination and nonsustainability rates among the highest reported, while keeping radiofrequency times among the lowest.^9^ See Online Data Supplement for further discussion about long-term ablation outcomes in patients.

### Limitations

Acute procedural end points, although very valuable from a mechanistic point of view, should be interpreted with caution because there is controversy about their value as predictors of long-term outcomes in patients.^26^ We performed coin-like sets of lesions,^25^ but other lesion strategies may potentially minimize radiofrequency delivery.^26^ Microanatomic reentry has been demonstrated to play a role in driving AF.^32^ However, anatomic imaging was not performed in this work to correlate leading-driver sites with the underlying anatomic/fibrotic substrate. Finally, our sheep and pig models are associated with HRAP but human PersAF can have a more complex and slowly evolving cause. Therefore, we included a limited number of complex PersAF patients to complete our translational approach. However, larger clinical series are warranted to further test this novel approach in patients and to quantify its potential synergistic value with respect to PVI or other conventional approaches, especially in cases with enlarged atria, complex causes, impaired ventricular function, or greater regional heterogeneity.

## Sources of Funding

This study was supported by the European Regional Development Fund and the Ministerio de Ciencia, Innovación y Universidades (MCNU; SAF2016-80324-R). The Centro Nacional de Investigaciones Cardiovasculares (CNIC) is supported by the Instituto de Salud Carlos III (ISCIII), the Ministerio de Ciencia, Innovación y Universidades (MCNU) and the Pro-CNIC Foundation, and is a Severo Ochoa Center of Excellence (SEV-2015-0505).

## Disclosures

J.G. Quintanilla, D. Filgueiras-Rama, N. Pérez-Castellano, and J. Pérez-Villacastín are coinventors on a pending patent application pertaining to the results presented in this article. The other authors report no conflicts.

## Supplementary Material

## Novelty and Significance

### What Is Known?

- Pulmonary vein isolation is the cornerstone of atrial fibrillation (AF) ablation although is still associated with suboptimal clinical outcomes in persistent AF (PersAF) as extrapulmonary vein regions may become relevant.
- Costly proprietary panoramic multielectrode (64–256) acquisition systems are being increasingly used to detect and ablate atrial regions with rotational activations (eg, rotors) regardless of their activation frequency dynamics.
- The role of such rotational activations in maintaining PersAF remains controversial.

## References

[1] Weng LC, Preis SR, Hulme OL, Larson MG, Choi SH, Wang B, Trinquart L, McManus DD, Staerk L, Lin H (2018). Genetic predisposition, clinical risk factor burden, and lifetime risk of atrial fibrillation.. Circulation.

[2] Kim MH, Johnston SS, Chu BC, Dalal MR, Schulman KL (2011). Estimation of total incremental health care costs in patients with atrial fibrillation in the United States.. Circ Cardiovasc Qual Outcomes.

[3] Quintanilla JG, Pérez-Villacastín J, Pérez-Castellano N, Pandit SV, Berenfeld O, Jalife J, Filgueiras-Rama D (2016). Mechanistic approaches to detect, target, and ablate the drivers of atrial fibrillation.. Circ Arrhythm Electrophysiol.

[4] Nattel S, Dobrev D (2017). Controversies about atrial fibrillation mechanisms: aiming for order in chaos and whether it matters.. Circ Res.

[5] Weiss JN, Qu Z, Shivkumar K (2016). Ablating atrial fibrillation: a translational science perspective for clinicians.. Heart Rhythm.

[6] Verma A, Jiang CY, Betts TR, Chen J, Deisenhofer I, Mantovan R, Macle L, Morillo CA, Haverkamp W, Weerasooriya R, STAR AF II Investigators (2015). Approaches to catheter ablation for persistent atrial fibrillation.. N Engl J Med.

[7] Haissaguerre M, Hocini M, Denis A, Shah AJ, Komatsu Y, Yamashita S, Daly M, Amraoui S, Zellerhoff S, Picat MQ (2014). Driver domains in persistent atrial fibrillation.. Circulation.

[8] Narayan SM, Krummen DE, Shivkumar K, Clopton P, Rappel WJ, Miller JM (2012). Treatment of atrial fibrillation by the ablation of localized sources: CONFIRM (Conventional Ablation for Atrial Fibrillation With or Without Focal Impulse and Rotor Modulation) trial.. J Am Coll Cardiol.

[9] Seitz J, Bars C, Théodore G, Beurtheret S, Lellouche N, Bremondy M, Ferracci A, Faure J, Penaranda G, Yamazaki M (2017). AF ablation guided by spatiotemporal electrogram dispersion without pulmonary vein isolation: a wholly patient-tailored approach.. J Am Coll Cardiol.

[10] Zaitsev AV, Berenfeld O, Mironov SF, Jalife J, Pertsov AM (2000). Distribution of excitation frequencies on the epicardial and endocardial surfaces of fibrillating ventricular wall of the sheep heart.. Circ Res.

[11] Filgueiras-Rama D, Price NF, Martins RP, Yamazaki M, Avula UM, Kaur K, Kalifa J, Ennis SR, Hwang E, Devabhaktuni V (2012). Long-term frequency gradients during persistent atrial fibrillation in sheep are associated with stable sources in the left atrium.. Circ Arrhythm Electrophysiol.

[12] Quintanilla JG, Moreno J, Archondo T, Chin A, Pérez-Castellano N, Usandizaga E, García-Torrent MJ, Molina-Morúa R, González P, Rodríguez-Bobada C (2013). K_ATP_ channel opening accelerates and stabilizes rotors in a swine heart model of ventricular fibrillation.. Cardiovasc Res.

[13] Kuklik P, Zeemering S, van Hunnik A, Maesen B, Pison L, Lau DH, Maessen J, Podziemski P, Meyer C, Schaffer B (2017). Identification of rotors during human atrial fibrillation using contact mapping and phase singularity detection: technical considerations.. IEEE Trans Biomed Eng.

[14] Roney CH, Cantwell CD, Bayer JD, Qureshi NA, Lim PB, Tweedy JH, Kanagaratnam P, Peters NS, Vigmond EJ, Ng FS (2017). Spatial resolution requirements for accurate identification of drivers of atrial fibrillation.. Circ Arrhythm Electrophysiol.

[15] Walters TE, Lee G, Spence S, Kalman JM (2016). The effect of electrode density on the interpretation of atrial activation patterns in epicardial mapping of human persistent atrial fibrillation.. Heart Rhythm.

[16] Baykaner T, Rogers AJ, Meckler GL, Zaman J, Navara R, Rodrigo M, Alhusseini M, Kowalewski CAB, Viswanathan MN, Narayan SM (2018). Clinical implications of ablation of drivers for atrial fibrillation: a systematic review and meta-analysis.. Circ Arrhythm Electrophysiol.

[17] Jarman JW, Wong T, Kojodjojo P, Spohr H, Davies JE, Roughton M, Francis DP, Kanagaratnam P, Markides V, Davies DW (2012). Spatiotemporal behavior of high dominant frequency during paroxysmal and persistent atrial fibrillation in the human left atrium.. Circ Arrhythm Electrophysiol.

[18] Lee G, Kumar S, Teh A, Madry A, Spence S, Larobina M, Goldblatt J, Brown R, Atkinson V, Moten S (2014). Epicardial wave mapping in human long-lasting persistent atrial fibrillation: transient rotational circuits, complex wavefronts, and disorganized activity.. Eur Heart J.

[19] Salinet JL, Tuan JH, Sandilands AJ, Stafford PJ, Schlindwein FS, Ng GA (2014). Distinctive patterns of dominant frequency trajectory behavior in drug-refractory persistent atrial fibrillation: preliminary characterization of spatiotemporal instability.. J Cardiovasc Electrophysiol.

[20] Gray RA, Jalife J, Panfilov AV, Baxter WT, Cabo C, Davidenko JM, Pertsov AM (1995). Mechanisms of cardiac fibrillation.. Science.

[21] Balasundaram K, Umapathy K, Jeyaratnam J, Niri A, Massé S, Farid T, Nair K, Asta J, Cusimano RJ, Vigmond E (2015). Tracking rotors with minimal electrodes: modulation index-based strategy.. Circ Arrhythm Electrophysiol.

[22] Martins RP, Kaur K, Hwang E, Ramirez RJ, Willis BC, Filgueiras-Rama D, Ennis SR, Takemoto Y, Ponce-Balbuena D, Zarzoso M (2014). Dominant frequency increase rate predicts transition from paroxysmal to long-term persistent atrial fibrillation.. Circulation.

[23] Gray RA, Pertsov AM, Jalife J (1998). Spatial and temporal organization during cardiac fibrillation.. Nature.

[24] Iyer AN, Gray RA (2001). An experimentalist’s approach to accurate localization of phase singularities during reentry.. Ann Biomed Eng.

[25] Atienza F, Almendral J, Ormaetxe JM, Moya A, Martínez-Alday JD, Hernández-Madrid A, Castellanos E, Arribas F, Arias MÁ, Tercedor L, RADAR-AF Investigators (2014). Comparison of radiofrequency catheter ablation of drivers and circumferential pulmonary vein isolation in atrial fibrillation: a noninferiority randomized multicenter RADAR-AF trial.. J Am Coll Cardiol.

[26] Calvo D, Rubín J, Pérez D, Morís C (2017). Ablation of rotor domains effectively modulates dynamics of human long-standing persistent atrial fibrillation.. Circ Arrhythm Electrophysiol.

[27] Li N, Csepe TA, Hansen BJ, Sul LV, Kalyanasundaram A, Zakharkin SO, Zhao J, Guha A, Van Wagoner DR, Kilic A (2016). Adenosine-Induced atrial fibrillation: localized reentrant drivers in lateral right atria due to heterogeneous expression of adenosine A1 receptors and GIRK4 subunits in the human heart.. Circulation.

[28] Ng J, Kadish AH, Goldberger JJ (2006). Effect of electrogram characteristics on the relationship of dominant frequency to atrial activation rate in atrial fibrillation.. Heart Rhythm.

[29] Lalani GG, Coysh T, Baykaner T, Zaman J, Hopper K, Schricker AA, Trikha R, Clopton P, Krummen DE, Narayan SM (2016). Organized sources are spatially conserved in recurrent compared to preablation atrial fibrillation: further evidence for non-random electrical substrates.. J Cardiovasc Electrophysiol.

[30] Romero J, Natale A, Di Biase L (2018). How to perform left atrial appendage electrical isolation using radiofrequency ablation.. Heart Rhythm.

[31] Santangeli P, Marchlinski FE (2017). Techniques for the provocation, localization, and ablation of non-pulmonary vein triggers for atrial fibrillation.. Heart Rhythm.

[32] Hansen BJ, Zhao J, Csepe TA, Moore BT, Li N, Jayne LA, Kalyanasundaram A, Lim P, Bratasz A, Powell KA (2015). Atrial fibrillation driven by micro-anatomic intramural re-entry revealed by simultaneous sub-epicardial and sub-endocardial optical mapping in explanted human hearts.. Eur Heart J.

