# Supplementary figures and images for: Instantaneous Amplitude and Frequency Modulations Detect the Footprint of Rotational Activity and Reveal Stable Driver Regions as Targets for Persistent Atrial Fibrillation Ablation
Source: Circ Res. 2019 Aug 29;125(6):609–27. doi: 10.1161/CIRCRESAHA.119.314930 (PMC6735936; doi:10.1161/CIRCRESAHA.119.314930)

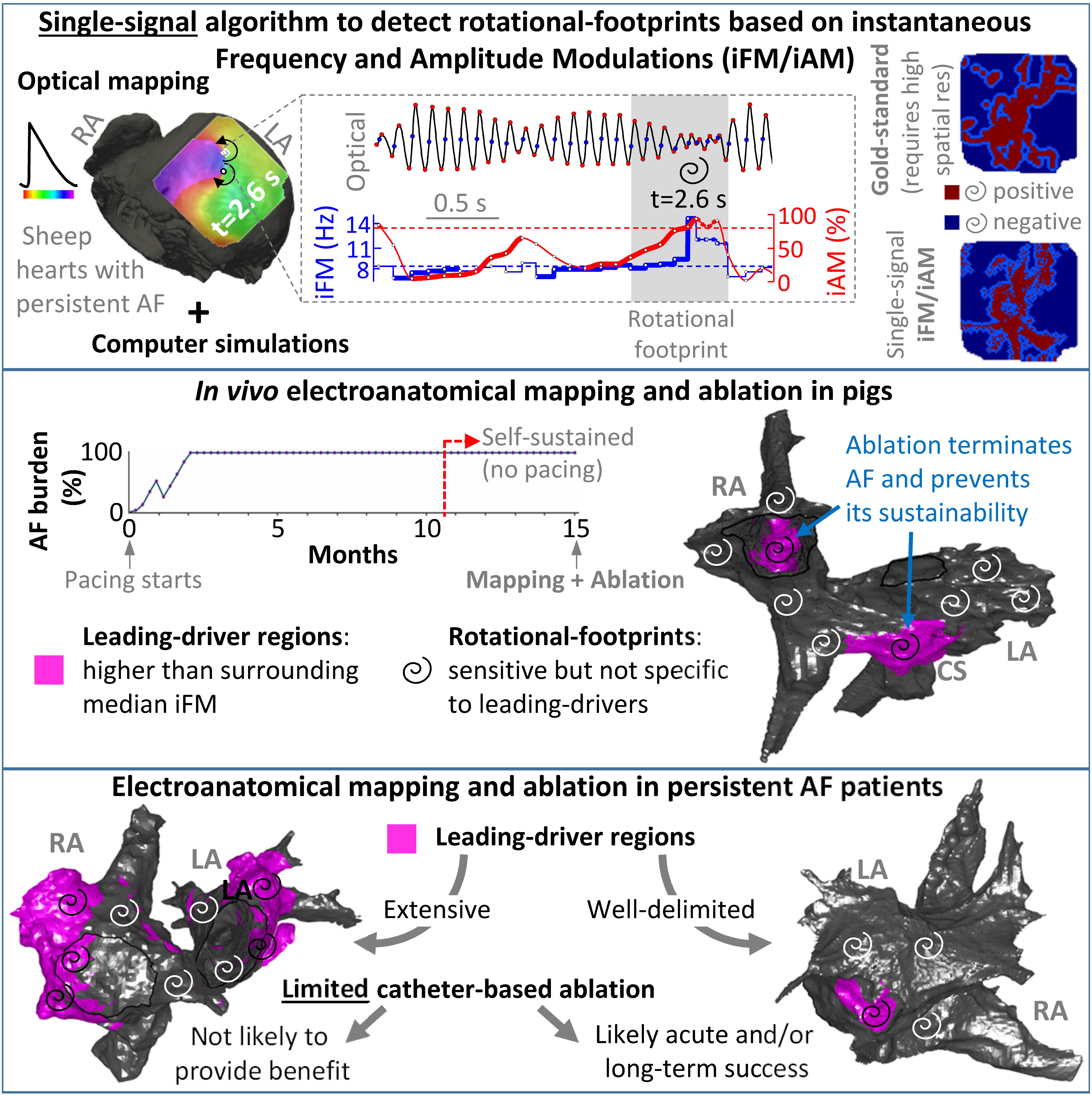

Supplement: Supplementary file 10 [file res-125-609-s010.jpg]
