# Supplementary material for: Instantaneous Amplitude and Frequency Modulations Detect the Footprint of Rotational Activity and Reveal Stable Driver Regions as Targets for Persistent Atrial Fibrillation Ablation
Source: Circ Res. 2019 Aug 29;125(6):609–27. doi: 10.1161/CIRCRESAHA.119.314930 (PMC6735936; doi:10.1161/CIRCRESAHA.119.314930)
Supplement: Supplementary file 12 [file res-125-609-s012.pdf]

SUPPLEMENTAL MATERIAL

Instantaneous amplitude and frequency modulations detect the footprint of rotational activity and reveal stable driver regions as targets for persistent atrial fibrillation ablation

Short title: **Robust targets for persistent AF ablation**

Jorge G. Quintanilla[1,2,3*] MScEng, PhD, José Manuel Alfonso-Almazán[1] MSc, Nicasio Pérez-Castellano[2,3] MD, PhD, Sandeep V. Pandit[4] PhD, José Jalife[1,3,4] MD, Julián Pérez-Villacastín[2,3] MD, PhD, David Filgueiras-Rama[1,2,3*] MD, PhD

[1] Centro Nacional de Investigaciones Cardiovasculares (CNIC). Madrid, Spain.

[2] Arrhythmia Unit. Cardiovascular Institute. Instituto de Investigación Sanitaria del Hospital Clínico San Carlos (IdISSC). Madrid, Spain.

[3] Centro de Investigación Biomédica en Red de Enfermedades Cardiovasculares (CIBERCV). Spain.

[4] Center for Arrhythmia Research. University of Michigan, Ann Arbor, USA.

Subject codes: Arrhythmias, Catheter Ablation, Atrial Fibrillation, Electrophysiology, Animal Models of Human Disease

***Joint corresponding authors:**

Jorge G. Quintanilla / David Filgueiras-Rama, Centro Nacional de Investigaciones Cardiovasculares (CNIC). Melchor Fernández Almagro, 3, 28029, Madrid. Spain. Telf.+34-914531200. Ext. 1503/1510.
E-mail: jgquintanilla@cnic.es / dfilgueiras@cnic.es

Non-standard Abbreviations and Acronyms

AF: atrial fibrillation

AM: amplitude modulation

CS: coronary sinus

DF: dominant frequency

EPS: electrophysiological study

FM: frequency modulation

HRAP: high rate (20 Hz) atrial pacing

iAM: instantaneous amplitude modulation

iFM: instantaneous frequency modulation

LA: left atrium

LAA: left atrial appendage

PMEAS: panoramic multielectrode (64-256) acquisition systems

PersAF: persistent atrial fibrillation

PS: phase singularity

PV: pulmonary veins

PVI: pulmonary vein isolation

RA: right atrium

RAA: right atrial appendage

RP_{min}: minimum refractory period

SNR: signal-to-noise ratio

VF: ventricular fibrillation

EXPANDED/SUPPLEMENTAL METHODS

Ovine and porcine experimental models of persistent AF

All procedures in sheep were approved by the University of Michigan Committee on Use and Care of Animals and complied with National Institutes of Health guidelines. The fast atrial pacing protocol used to generate a persistent AF model in sheep has been previously reported in detail elsewhere.[1] Sheep (≈40 kg, 6-8 month old) were used for implantation. Anesthesia was induced using propofol (4−6 mg/kg IV) and maintained by isoflurane inhalation. A bipolar lead was inserted into the RA appendage (RAA) through the left external jugular vein. The proximal end of the lead was screwed to the sterile pacemaker (Victory XLDR; St Jude Medical, Sylmar, CA), which was sheltered in a subcutaneous pouch. After 10 days of recovery, the pacemaker was programmed to induce AF by high rate atrial pacing (HRAP) with an algorithm consisting of 30-second pacing at 20 Hz followed by 10-second sensing. The pacemaker was interrogated weekly during the study period. Importantly, to generate a clinically relevant persistent AF model, the pacing algorithm was stopped after 20-24 weeks. Persistent AF was defined based on the criteria used for human AF as those episodes lasting >7 days upon switching off the fast pacing program.[1]

All procedures in pigs were approved by the CNIC Committee on Use and Care of Animals and complied with institutional, Spanish (RD53/2013, ECC/566/2015) and European (2010/63/EU) guidelines. We used slow growing cross breeding Yucatan - Large white pigs to avoid problems arising from big animals after long periods in AF. All pacemaker implantations were carried out under general anesthesia. Pigs were pre-medicated with a combination of intramuscular Ketamine (20 mg/kg i.m.) and Midazolam (0.5 mg/kg i.m.). Anesthesia was induced with an intravenous bolus of Fentanyl (0.010 mg/kg i.v.), which allowed oral tracheal intubation. After intubation animals were mechanically ventilated with intermittent positive pressure and anesthesia was maintained by a combination of Fentanyl (0.005 mg/kg/h i.v.) and Sevoflurane (2%).

Unlike the sheep model,[1, 2] in the pig model it is necessary to ablate the atrio-ventricular node and implant a second lead to pace the ventricles at physiological rates in order to avoid fast ventricular rates, which would lead to heart failure.[3] Therefore, we implanted a dual chamber pacemaker with atrial and ventricular leads that were inserted into the RAA and right ventricular apex, respectively. Intravenous cefazoline (25 mg/Kg, Laboratorios Normon, Madrid) was administered 10 min before the procedure and 2 hours after the first dose to prevent surgical site infections. Intramuscular ceftiofur (3 mg/Kg, Laboratorios Hipra S.A., Girona) was also administered every 24 hours for more 3 days. After 10-12 days of recovery, all pigs (age: 5.2[4.5-6.1]-month-old, weight: 44.0[40.0-45.5] kg) underwent atrioventricular node ablation before attempting HRAP. More specifically, the pacemakers were programmed to induce AF by a 30-second burst of HRAP (20 Hz, twice diastolic threshold) followed by a 6-second sensing period, while the ventricles were continuously paced using a physiological rate modulation mode between 60-110 beats per minute. Upon sinus rhythm detection, HRAP was automatically resumed until AF was self-sustained and HRAP was no longer necessary. Atrial electrograms were stored using the automatic mode switch algorithm of the pacemakers to generate AF burden curves and follow AF evolution from initial paroxysmal episodes to PersAF episodes lasting >7 days without HRAP. This protocol allows the generation of a model that progressively evolves from short-lasting AF episodes to long-lasting persistent AF using clinical criteria. The aforementioned has special relevance, as from the moment AF becomes persistent the pacemaker does no longer need to reinitiate HRAP. All animals underwent a sequential follow every 3 weeks including pacemaker interrogation and transthoracic echocardiogram.

Left ventricular ejection fraction was measured using the Teichholz formula and M-mode echocardiography data. The maximum minor axis of the left ventricle at end-diastole and end-systole was measured on the parasternal long-axis or short-axis view.

The animal models of PersAF presented above were used as follows:

First, high spatiotemporal resolution optical mapping data from Langendorff-perfused PersAF sheep hearts were used as the 'gold-standard' to test the ability of the iFM/iAM algorithm to detect rotational-footprints using single-signals.

Second, we used the electroanatomical mapping data from the first 4/16 pigs with self-sustained PersAF (development group) to adapt the ex vivo iFM/iAM optical single-signal processing to the in-vivo unipolar electrical signals, which display a different morphology and ventricular far-field that is not present in optical signals. Proper validation of the single-signal iFM/iAM algorithm to detect rotational-footprints would have not been possible using the electroanatomical data acquired in vivo from PersAF pigs due to its intrinsically low spatial resolution. In addition, phase mapping of electrical data requires multiple signals, is completely dependent on electrode location, contact, separation and filtering, and has low specificity for rotor detection.[4-6] Indeed, electrical phase movies using multispline catheters may be unreliable when catheters are not well deployed and/or good tissue contact is not ensured.[4-6] Also, even if electrodes are well deployed and tissue contact is fine, some unpredictable issues may arise when using the phase of signals interpolated from sparse data[7] (see *Mapping approaches to detect rotational activation and drivers* section in the Discussion of the main manuscript).

Third, sham-operated animals were used as controls to ensure that our pig model of self-sustained PersAF developed significant atrial dilatation and fibrosis without compromising systolic ventricular function.

Finally, the remaining 12/16 pigs with self-sustained PersAF (ablation group) were used i) to demonstrate the ability of iFM to detect leading-driver regions that are key for the sustainability of PersAF, ii) to test their spatiotemporal stability, and iii) to determine whether rotational activity (detected with the previously validated single-signal iFM/iAM algorithm) is specific for such leading-driver regions. Further details are provided in the next sections.

Ex vivo optical mapping of sheep hearts with persistent AF

Optical mapping movies were obtained from five sheep hearts (≈66 kg) with persistent AF after 5-6 months of fast atrial pacing. Details regarding heart excision and preparation for optical mapping were reported in detail elsewhere.[1] Briefly, upon heart excision and aortic cannulation, the hearts were Langendorff-perfused with Tyrode's solution. After atrial trans-septal puncture, all the vein orifices were sealed except the inferior vena cava, which was connected to a digital sensor and to an open-end cannula to control the intra-atrial pressure. The intracavitary pressure was increased to a subthreshold AF-inducing level of 5 cm H_2O, resembling the diastolic LA pressure and maintained throughout the experiment. Optical movies of the epicardial surface of left atrial appendage (LAA) were acquired after dye loading with Di-4-ANEPPS 5-10 mg/mL (532 nm laser excitation) and under Blebbistatin (10 µM). The emitted fluorescence (645 nm) was projected onto a charge-coupled device (CCD) camera (80x80 pixels, 600 frames/sec). Spontaneous AF was allowed to continue uninterruptedly for 50 minutes. Five-second long optical movies were acquired at 2-minute intervals (25 movies/sheep, 125 movies total).

Ex vivo optical signal processing

Filtering, phase movies and phase singularity detection

After low pass spatiotemporal filtering with a conical-shaped kernel, and drift removal when present (subtraction of the output of a running average filter),[1, 8-12] phase movies were obtained by means of Hilbert transformation of the optical action potentials.[13] A phase singularity (PS) was defined as the pivoting point where all phases converged during rotational activation.[14] Specifically, a PS occurs when the line integral of the change of phase around a site equals $\pm 2\pi$ where the sign indicates chirality:[15]

$$\oint_C \nabla\theta(x,y) \cdot \overrightarrow{dr} = \pm 2\pi$$

where $\theta(x, y)$ is the instantaneous value of phase at site (x, y), and *C* is a closed path that surrounds the site. PS detection was implemented in Matlab using the computational method previously described by Iyer & Gray.[15] Briefly, the PS-finding algorithm evaluates the difference in phase over a discrete path. If the phase difference is equal to $\pm 2\pi$ the path encloses a PS. Since large path lengths decrease precision in localizing PSs, we used a square path length of eight pixels, which was the approach recommended in the original reference,[15] where further specific details about the implementation are reported.

Computation of time-domain iFM and iAM

Online Figure I describes in detail the algorithm used to calculate instantaneous frequency modulation (iFM), instantaneous amplitude modulation (iAM) and rotational-footprint detection using the optical signal displayed in **Figure 2A** as example.

The optical signal showed in **Online Figure I** (row 1) corresponds to a specific pixel of an optical movie (80x80=6400 pixels) previously filtered as described above. Note that the same processing was performed for every pixel in the optical movie. First, the slope (first derivative: dV/dt) of the optical signal was computed. Since activation times in optical action potentials are usually defined at the times of maximum positive slope within the phase-0, the intervals in which the slope was negative did not provide useful information regarding activation times and were assigned a zero value. This way, a signal with peaks corresponding only to intervals of positive slope was obtained (absolute positive slope, APS, row 2 in **Online Figure I**). This signal can be easily calculated as follows:

$$APS(t) = \frac{1}{2}\left(\left|\frac{d\ OAP(t)}{dt}\right| + \frac{d\ OAP(t)}{dt}\right)(\frac{au}{ms})$$

where *OAP(t)* is the optical signal. Then, the times when peaks in *APS(t)* met the following criteria were selected as activation times: i) a value above a minimum slope threshold (minimum slope), ii) a prominence above a certain value (minimum prominence), and iii) separated at least a minimum refractory period from the previous and the next peaks.

The minimum slope was set as the fiftieth part (2%) of the 95th percentile of the peak amplitude values. Similarly, the minimum prominence was set as the fiftieth part (2%) of the 95th percentile of the peak prominence values:

$$APS_{min} = 0.02 \cdot P_{95th}(APS)\ au/ms$$

The 95th percentiles were used as reference instead the maximum values to prevent excessive thresholds due to artifactually high values in the optical signal slope:

The minimum refractory period was set for each pixel as:

$$RP_{min} = max\left\{50\ ms, \frac{1000}{1.95 \cdot min\{DF_{OAP}, DF_{APS}\}}\ ms\right\}$$

where DF_{OAP} is the dominant frequency of the optical action potentials and DF_{APS} the dominant frequency of the *APS(t)* signal. DF_{OAP} and DF_{APS} were calculated as the frequencies with the highest peak in the power spectral density (PSD) of the *OAP(t)* and *APS(t)* signals respectively. PSD was estimated by a conventional periodogram of the signal previously multiplied by a Kaiser window (β=2.5) and zero-padded to the next higher power of 2.

Both dominant frequencies are used as a rough surrogate of the reciprocal of the average interval between activations. The 1000 factor is used to convert that time into ms. Since activation intervals shorten and lengthen dynamically during fibrillation, the *1/1.95* factor is used to allow for intervals up to 1.95 times shorter than the surrogate average one [*(1/DF)*1000* ms]. Such a value usually provides a reasonable lower limit for the local refractory period. Nevertheless, if that value was lower than 50 ms, the latter was used as the minimum refractory period for the algorithm.[16] The resulting activation times are displayed as blue dots in row 3 of **Online Figure I**. Despite such a lower limit for the refractory period can account for physiological dynamic shortening of the local activation intervals, it sometimes may result in too short, artificial intervals. Therefore, an iterative adaptive algorithm was used to refine activations by allowing only physiological changes in atrial activation cycles. This was done by 1) inserting potential activations within too long, non-physiological cycles if found and/or 2) excluding potential activations when too short non-physiological cycles were found and 3) adjusting potential activation times to the peak in the APS signal that prevents abrupt and non-physiological inter-activation cycle changes, when very close peaks of similar height were present in the APS signal. In the signal showed in **Online Figure I**, row 4, no changes in activation times were performed by the adaptive iterative algorithm.

In addition, the amplitude of the phase-0 of the action potentials was calculated as the fluorescence excursion (arbitrary units) between the times at which the slope goes below the 4% of the slope at the activation time. **Online Figure I**, row 5, shows an example of the fluorescence values at the activation times (blue dots) and their corresponding lower and upper amplitude limits for the phase-0 (red dots). By interpolating such amplitude excursions, we obtained the optical envelope signal (**Online Figure I**, *OE(t)*, row 6). Since these amplitudes dynamically change (i.e. are modulated by several factors), their corresponding instantaneous amplitude modulation (iAM) signal (**Online Figure I**, row 9, red trace) was calculated as follows:

$$iAM(t) = 100\left(1 - \frac{OE(t)}{\max\{OE(t)\}}\right)(\%)$$

Importantly, iAM is not an absolute measurement but a relative one. Thus, the lower the optical envelope (normalized by its maximum value during the 5-second acquisition), the higher the iAM is. Intervals with low iAM values correspond to times when the optical envelope is unaffected, whereas intervals with high iAM values correspond to times when the optical envelope is highly affected by some factor/s (e.g. a drifting rotor is passing nearby). Despite photobleaching affects the amplitude of the acquired optical signals in the medium term (minutes to hours), it is not expected to have a relevant effect during intervals

as short as 5 seconds. Indeed, note that although photobleaching may be significantly different between different movies taken minutes apart, it does not constitute a problem for *iAM(t)* calculation because *OE(t)* is normalized with the maximum OE value for each optical signal within the 5-second interval of acquisition.

Also, a frequency modulated signal (*FM(t)*) was obtained by adjusting a sinusoidal wave in order to reach its maxima at the previously detected activation times (**Online Figure I**, row 7). This way, the distribution of the FM sinusoidal cycles directly reflects the dynamic changes in tissue activation rate at any specific location.

Furthermore, a frequency- and amplitude-modulated (FM|AM) signal was obtained by multiplying the FM signal and the optical envelope signal (**Online Figure I**, row 8):

$$FM|AM(t) = OE(t) \cdot FM(t)$$

In addition to directly reflect in an intuitive way the dynamic changes in the tissue activation rate at any specific location, this FM|AM signal displays the dynamic changes in the amplitude of the phase-0.

Finally, the instantaneous frequency value between 2 consecutive activation times was calculated as the inverse of the interval between those activations in seconds. Analytically, given N consecutive activation times in ms ($t_0, t_1, \dots, t_N$), the *iFM(t)* signal is calculated as follows:

$$iFM(t) = \sum_{n=0}^{N-1} \frac{1000}{t_{n+1} - t_n} [u(t - t_n) - u(t - t_{n+1})]$$

where $u(t)$ is the Heaviside unit step function, defined as $u(t) = \begin{cases} 0, t < 0 \\ 1, t \geq 0 \end{cases}$

Row 9 in **Online Figure I** displays the resulting instantaneous frequency modulation (iFM) signal (blue trace).

Rotational-footprint detection

The parameters included in the algorithm (minimum number of increasing iFM cycles, minimum number of increasing iAM cycles, iAM threshold, minimum iAM excursion, and minimum iFM percentile) and the logical conditions that implemented rotational-footprint detection were theoretically derived. At the phase singularity (rotor tip), the curvature of the spiral wavefront reaches a critical value that makes it impossible for the activity to invade the rotor core. The latter results in decreased voltages in the vicinity of the rotor tip. Therefore, as a drifting scroll-wave filament/rotor core approaches a tissue spot, the amplitude of the action potentials should decrease resulting in an increased iAM. Simultaneously, as the wave-emitting source (scroll-wave filament/rotor core) is approaching, the perceived iFM at the spot should increase due to Doppler Effect. Therefore, a simultaneous iAM/iFM increase may be indicative of drifting scroll-waves/rotors in the surroundings. However, in order to achieve more specificity, the algorithm requires a 'minimum number of increasing iFM cycles' and a 'minimum number of increasing iAM cycles'. Also, if the drifting rotor tip actually crosses the spot, the amplitude of the action potentials at that location and that very instant should be theoretically zero. For this reason, it is required that iAM goes above a 'minimum iAM threshold' for positive rotational-footprint detection. In addition, a 'minimum iAM excursion' is also required because slight changes in iAM, even when going above the 'minimum iAM threshold' may not be related with a rotor tip crossing the spot. A fifth parameter was also included in the

algorithm to make it more sensitive for quasi-stationary/meandering rotors. Since these types of rotors are not necessarily approaching the spot but surrounding it, they do not necessarily result in an iFM increase at the spot. However, near their cores, iAM should be high (low voltages, iAM above the 'minimum iAM threshold') and iFM should be also high (above a 'minimum iFM percentile'). Taking all of these into consideration, single-signal detection of a rotational-footprint was based on compliance with one of the following conditions:

Condition A: a simultaneous increase in iFM and iAM, which is more specific to drifting rotors approaching a spot (e.g. rotors 1 and 2 in rows 9/10 of **Online Figure I**). Specifically, the following logical condition must be fulfilled:

Increasing *iFM(t)* for at least *parameter1* cycles AND [(increasing *iAM(t)* with a minimum excursion of *parameter2* % for at least *parameter3* cycles reaching at least *parameter4* %) OR *iAM(t)* ≥ *parameter4* %]

Condition B: simultaneous high iAM and iFM values, which is more sensitive to quasi-stationary rotors or rotors meandering around a location (e.g. rotor 3 in rows 9/10 of **Online Figure I**) which do not necessarily present Doppler effect since they are not necessarily approaching the location but surrounding it. Specifically, the following logical condition must be fulfilled:

iFM(t) ≥ *parameter5* percentile AND *iAM(t)* over *parameter4* % for at least 2 cycles

In the examples shown in **Figures 2**, **4** and **Online Figures I**, **III**, **IV** and **XVII** the following parameters were used:

parameter1= 4 (minimum number of increasing iFM cycles)

parameter2= 25 % (minimum iAM excursion)

parameter3= 3 (minimum number of increasing iAM cycles)

parameter4= 80 % (iAM threshold)

parameter5= 70th percentile (minimum iFM percentile without increasing iFM together with iAM ≥ parameter4)

As reported in the results section, this combination of parameters provided the maximal sum of sensitivity and specificity using a 1.25 mm tolerance.

Note that if iAM remains above *parameter4* %, the rotational-footprint is still detected after the end of the increasing iFM cycles in condition A. In other words, after simultaneously increasing iAM and iFM intervals, the algorithm considers that the rotor is still around there while iAM keeps over the *parameter4* threshold regardless of iFM. That specific detail of the algorithm is warranted by the extremely low amplitude/high frequency of the optical action potentials during such intervals, which can result in infra-detection of actual complete or partial depolarizations for not fulfilling the algorithm criteria. Examples of this kind of situation are shown in **Online Figure II** and **Online Movie II**.

Online Figure III shows the results of the algorithm if the minimum refractory period is established at 50 ms regardless of the dominant frequencies of the OAP and APS signals. Such a change achieves that the low amplitude/high frequency partial depolarizations displayed in **Online Figure II** and **Online Movie II** due

to the proximity of a meandering rotational core are not been ignored. However, despite the fact that this approach might be more sensitive to detect rotors that are stationary or meander around the pixel, introduces the risk of considering other potential causes of signal change (impulsive noise, depolarizations at the contralateral layer of the atria,[17, 18] etc.) as actual partial or complete depolarizations. That is the reason why the algorithm was henceforth applied with the lower limit of the refractory period calculated from the dominant frequencies of the OAP and APS signals (algorithm version shown in **Online Figure I**). Anyway, the rotational-footprint positive intervals eventually detected by the two versions of the algorithm were practically the same. This was thanks to the algorithm detail described above and marked with an asterisk in **Online Figure I**, which enables to still detect the presence of a rotor in intervals of very low amplitude after simultaneously increasing iAM and iFM, but without the counterpart of over-sensing risks.

Online Figure IV shows additional examples of the processing applied to single optical signals to detect the footprint of rotational activity.

Assessment of the sensitivity and specificity of the iFM/iAM algorithm to detect rotational-footprints

The optimal values for the parameters were determined by quantifying the sensitivity and specificity of all possible parameter combinations (with the parameter taking values in reasonable ranges, see **Online Table I**) in 117 optical mapping movies from 5 sheep hearts with persistent AF. For each parameter combination, we calculated the sensitivity, specificity and their sum for every movie out of the 117, considering all 117 movies to obtain mean (SD) values. This process was repeated for each one of the 8820 parameter combinations tested, and considering spatial tolerances of 0, 1.25 and 2.5 mm.

Of course, the algorithm can display its maximal sensitivity, specificity or sensitivity+specificity using a slightly different combination of parameters for each optical movie. Indeed, the optimal parameter combinations were similar but not exactly the same for all the movies. For this reason we did not rely on parameter combinations that were optimal for individual movies, but on the parameter combinations that, considering all the 117 movies: 1) maximized the average sum of sensitivity and specificity, or 2) maximized the average sensitivity as long as the average specificity was ≥95% or ≥97.5%, or 3) maximized the average specificity as long as the average sensitivity was ≥95% or 97.5%. Therefore, parameters do not need to be adjusted for different types of experiments or different individual experiments because they are calculated to provide the best average performance for all the movies analyzed. However, one could select a different set of parameter values if wants to prioritize sensitivity over specificity, or vice versa.

The tested parameter combinations were summarized in **Online Table I.** The results of the analysis of all tested combinations are shown in **Online Table II**. It displays the parameter combinations that maximized: 1) sensitivity+specificity, 2) sensitivity as long as specificity was ≥95%, and 3) specificity as long as sensitivity ≥95%, for any number of parameters (from 1 to 5). This analysis showed that some of the theoretically derived parameters were much more relevant than others. In fact, the most relevant ones were the 'iAM threshold' and the 'minimum number of increasing iFM cycles'. As shown in **Online Table II**, pretty decent values of sensitivity and specificity can be achieved by only using those 2 parameters considering tolerances ≥1.25 mm. Indeed, the introduction of the other 3 parameters only slightly improves the sum of sensitivity and specificity. However, since the sum of sensitivity and specificity is maximized when all the 5 parameters are used, we reported a summary of the combinations of the 5 parameters that provided the highest values of 1) sensitivity+specificity, 2) sensitivity as long as specificity ≥97.5%, and 3) specificity as long as sensitivity ≥97.5% in **Figure 4D**. This figure panel also specified which

parameter combination was used for the optical mapping examples, and which one was chosen for in vivo experiments.

Sensitivity and specificity of the rotational footprint detection algorithm were calculated using conventional criteria:

$$Sensitivity = \frac{True\ Positives}{True\ Positives + False\ Negatives}$$

$$Specificity = \frac{True\ Negatives}{True\ Negatives + \ False\ Positives}$$

True positives: pixels actually crossed by a phase singularity (pivoting point of a rotational activation) that were classified as rotational-footprint positive by the single-signal iFM/iAM algorithm.

True negatives: pixels not crossed by a phase singularity that were classified as rotational-footprint negative by the single-signal iFM/iAM algorithm.

False positives: pixels not crossed by a phase singularity that were classified as rotational-footprint positive by the single-signal iFM/iAM algorithm.

False negatives: pixels actually crossed by a phase singularity that were classified as rotational-footprint negative by the single-signal iFM/iAM algorithm.

Those were the definitions used at the pixel level (i.e. when tolerance was fixed to 0 mm). However, in a real scenario, if an ablation catheter were centered at one pixel location positive for phase singularities and radiofrequency energy were delivered there, the created lesion would be much larger than the actual pixel size. Therefore, we also calculated sensitivity and specificity of the iFM/iAM algorithm allowing tolerances of 1.25 mm (radius of a conventional ablation catheter) and 2.5 mm (diameter of a conventional ablation catheter). Note that these tolerances are smaller than the actual lesions created by a conventional ablation catheter.[19, 20] Thus, the definitions were modified as follows when a tolerance of 1.25 or 2.5 mm was considered:

True positives: pixels actually crossed by a phase singularity that were classified as rotational-footprint positive or were within a 1.25/2.5 mm distance from those actually classified as rotational-footprint positive by the single-signal iFM/iAM algorithm.

True negatives: pixels not crossed by a phase singularity and beyond a 1.25/2.5 mm distance from those actually crossed by a phase singularity, that were classified as rotational-footprint negative by the single-signal iFM/iAM algorithm.

False positives: pixels not crossed by a phase singularity and beyond a 1.25/2.5 mm distance from those actually crossed by a phase singularity, that were classified as rotational-footprint positive by the single-signal iFM/iAM algorithm.

False negatives: pixels actually crossed by a phase singularity that were classified as rotational-footprint negative and were beyond a 1.25/2.5 mm distance from those actually classified as rotational-footprint positive by the single-signal iFM/iAM algorithm.

Computer simulations

We further tested the new algorithms presented in this study using previously published simulations of rotors generated with computer models of PersAF.[21, 22] These previous papers contain the implementation methods. Briefly, the equations and various parameters for the cellular ionic models used in the human PersAF and sheep PersAF simulations were derived from atrial ionic models by Courtemanche et al.[23] and Grandi et al.[24], respectively. The parameters and equations for both ionic atrial models are freely available in Pubmed. The cellular ionic models were then incorporated into 2D sheets to generate rotors, and implemented in the “C” language and standard message passing interface (MPI) on parallel processors.[21, 22]

More specifically, the propagation of the cardiac impulse was simulated in a two-dimensional (2D), homogeneous, isotropic tissue of 5x5 cm^2 (human) or 6x6 cm^2 (sheep), consisting of 500x500 atrial cells (human) or 600x600 atrial cells (sheep), and no-flux boundary conditions at the edges. The Euler method was used to integrate the voltage at each node, which was governed by the conventional reaction-diffusion equation, assuming uniform, isotropic tissue:

$$\frac{\partial V_m}{\partial t} = -\frac{I_{ion}}{C_m} + D\left(\frac{\partial^2 V_m}{\partial x^2} + \frac{\partial^2 V_m}{\partial y^2}\right)$$

where V_m is the membrane voltage, I_{ion} is the total membrane ionic current, C_m is the membrane capacitance, and D is the diffusion coefficient. Rotors were initiated by the standard cross-field stimulation protocol, and the rotational activity was studied for 4 s (sheep) or 10 s (human). In addition to baseline conditions for PersAF, the effect of modifying or blocking specific currents (I_{Kur} and I_{K1} in sheep, I_{K1}, $I_{Ca\text{-}L}$, I_{Kr} and I_{Ks} in human) was also simulated to test the algorithm with a wider range of spiral-wave tip meandering behaviors (**Online Figure V**). More specific details and changes in conductance values can be found in the previous studies that incorporated and described the sheep[22] and human[21] simulations of PersAF.

Additionally, in this study, for each simulated epoch, a uniform mesh of unipolar electrograms was calculated under the assumption of a homogenous, unbounded, and quasi-static conducting medium by summing up all effective dipole contributions over the entire 2-D sheet at each timestep as follows:

$$V_{uni}(\vec{r}) = \sum_{i=1}^{M}\sum_{j=1}^{N} D\,\frac{\nabla V_m(\vec{r})\cdot(\vec{r}-\overrightarrow{r_{IJ}})}{\left|\vec{r}-\overrightarrow{r_{IJ}}\right|^3}$$

where $V_{uni}(\vec{r})$ is the unipolar signal generated at location $\vec{r}$ within the 5x5 cm^2 (human) or 6x6 cm^2 (sheep) 2D sheet, M the number of elements in the 2D sheet in the row dimension, N the number of elements in the 2D sheet in the column dimension and $\overrightarrow{r_{IJ}}$ the location of each contributing element in the 2D sheet. In addition, the effect of the unipolar electrode size was simulated by creating unipolar signals as the sum of all unipolar contributions within the surface covered by a 1 mm^2 electrode (size of the PentaRay electrodes used during our *in vivo* experiments).

Then, the sensitivity and specificity of the single-signal rotational-footprint detection algorithm was quantified using both transmembrane potentials and unipolar electrograms (with pixel and electrode sizes) in the simulated movies under multiple combinations of algorithm parameters.

In addition, to quantifying the effect of noise on the detection of rotational footprints and leading-driver regions, different levels of additive white gaussian noise (signal-to-noise rations, SNR=∞, 30, 20, 10 and 0 dB) were added to the simulated signals before performing a conventional unipolar filtering (0.05-300 Hz, similar to most electrophysiological recording and electroanatomical mapping systems). Low-pass filtering (300 Hz) alone was applied to noisy transmembrane action potentials. SNR was quantified as follows:

$$SNR\ (dB) = 10 \log_{10} \frac{P_{signal}\ (\mu W)}{P_{noise}\ (\mu W)}$$

where *P* is power. Using these noisy signals, we quantified *i)* the sensitivity and specificity of the iFM/iAM rotational-footprint detection algorithm, and *ii)* the accuracy in the measurement of iFM_{median} (that is the value used to detect leading-driver regions). The 'gold-standard' iFM signal that was used as reference to quantify the error, was obtained detecting the times of the maximum slopes within the phases 0 of the simulated transmembrane action potentials devoid of noise. Only depolarizations that reached the atrial threshold potential (-60 mV) were considered true phase-0 upstrokes. Also, since depolarizations starting well above the resting potential are usually observed in computer models of fibrillation, only those that reached the atrial threshold potential with an ascending potential excursion of at least 20 mV were considered true action potentials.

Of note, when comparing and interpreting the results obtained from optical mapping data and computer simulations, it is important to consider the differences between them. Thus, the rotors induced by cross-field stimulation in computer simulations of PersAF were mostly quasi-stationary/meandering, likely because of the homogeneous and isotropic nature of the simulated 2D sheet. Conversely, the prevalence of quasi-stationary rotors in our optical mapping data was extremely small and most rotors displayed a drifting behavior. Therefore, Doppler effect would be less noticeable in computer simulations than in experimental data. Also, the models we used were two-dimensional, but atrial tissue, although thin, is actually three-dimensional. Therefore, in computer simulations, rotor cores had 1 less dimension to approach or retreat from the location where iFM and iAM were being measured than had potential scroll-wave filaments in *ex vivo* or *in vivo* experimental scenarios. Differences in restitution properties between ionic models of PersAF and actually remodeled tissue in experimental animal models of self-sustained PersAF, may also lead to differences in the iFM and iAM patterns of increase (e.g. the number of cycles they are present or the maximum values they reach) associated with a rotor phase singularity/scroll-wave filament approaching the analyzed spot.

In vivo electroanatomical mapping of pigs with PersAF

All *in vivo* procedures were performed under general anesthesia as described for pacemaker implantations. Vital signs were monitored during the procedure to control anesthesia status. The invasive electrophysiology study was performed using percutaneous venous and arterial femoral access to reach the atrial chambers and continuously monitor the blood pressure, respectively. Electrical and blood pressure signals were continuously monitored using the LabSystem-Pro electrophysiology recording system (Boston Scientific, Lowell, MA). During the mapping procedure, ventricular pacing was adjusted between 40 and 60 beats per minute to minimize the number of ventricular artifacts on atrial signals while ensuring appropriate pressures. Mapping and ablation procedures were guided with the Ensite Precision (Abbott, Princeton, NJ). A decapolar catheter was positioned into the superior vena cava, assessing that the signal of the distal bipole was negligible. Then, the distal electrode was used as unipolar reference. AF maps were obtained using the CFE-mean mode available in the Ensite Precision system. Although CFE data

were not used, it enabled us to store 8-second unipolar signals at each location. More specifically, a PentaRay catheter (20 poles, Biosense Webster, Diamond Bar, CA) was introduced through a long steerable sheath (8.5 F Agilis NxT Steerable Introducer, 91 cm Lumen Length. Abbott, St. Paul, MN) to sequentially position the catheter at different locations of the right atrial (RA) endocardium and coronary sinus (CS). The PentaRay catheter was connected to the pin box of the electrophysiology recording system using a custom-made adaptor. A continuous flow (200 ml/h) of heparinized saline (1000 IU/500 mL) was administered through the long sheath to prevent luminal clot formation. Eight-second long unipolar signals were acquired at each location, while we reconstructed the atrial anatomy. An ECG lead was simultaneously stored to feed an off-line algorithm that minimized the ventricular far-field in the unipolar recordings. Catheter and electrogram stabilities during the 8-second window were ensured using the tool implemented in the Ensite Precision system for that purpose. Upon completion of the RA and CS maps, a transeptal puncture was performed to get access to the left atrium (LA) and sequentially acquire signals as in the RA chamber and CS. Heparin (130 IU/kg) was administered to avoid thrombi formation after the transeptal puncture. One-to-two hours later after completion of the first map, we started a second biatrial map to test for spatiotemporal stability between the first and second sets of data.

Electroanatomical mapping and ablation protocol in patients

Electroanatomical mapping was performed in an identical manner as in the pig model to complete the translational approach. The unipolar reference was obtained from the distal electrode of a decapolar catheter positioned into a hepatic vein. Based on the temporal stability of the driver maps in the pig model, we only acquired one biatrial map in patients to avoid unnecessary prolongation of the procedure. The absence of electrical activity inside the PVs was used to confirm PVI. Intravenous heparin (140 IU/Kg) was administered to maintain an activated clotting time >300 seconds throughout the procedure. Data acquisition was performed in AF using the Ensite Precision System and a PentaRay catheter, which was sequentially positioned at different right and left atrial locations. The PentaRay was connected to the pin box of the electrophysiology recording system (Prucka CardioLab, General Electric Medical, Milwaukee, WI) using a custom-made adaptor. Data from the CS was obtained with the ablation catheter (FlexAbility, Abbott) before LA mapping. Mapping data were exported and processed intra-procedure. After signal processing (~10 min), the ablation targets were shown in a second screen next to the Ensite Precision display. An open-irrigated tip catheter was used to deliver radiofrequency energy on the leading-driver locations (30-35W, saline irrigation: 17 ml/min). The physician in charge could stop ablation if the leading-driver regions were considered too large to be safely and effectively ablated with catheter-based radiofrequency delivery. In case of AF termination during radiofrequency delivery, AF reinduction was attempted as in the pig model. Any atrial tachycardia/flutter occurring after AF termination/reinduction were also mapped and ablated. If AF persisted after stopping ablation, it was cardioverted and the procedure terminated after further confirmation of PVI. All patients underwent clinical follow-up to assess recurrences at 1, 3, 6, 9, 12 and 16 months after the procedure.

In vivo electrical signal processing

Ventricular and pacing artifact minimization and rejection

An estimation of the ventricular far-field was subtracted from atrial unipolar signals. This estimation was based on Principal Component Analysis (PCA), as described elsewhere.[1] More specifically, the PCA method was applied to a window around the R-peak of the QRS complex (orange intervals in **Online Figure VI A**), which comprised from the beginning of the Q-wave (or the spike of the stimulus if ventricular pacing was

present: cyan spikes in **Online Figure VI A**) to the end of the S-wave. The same window size was used for all the QRS complexes in a unipolar signal (size of the longest spike + QRS interval in the corresponding simultaneous ECG, **Online Figure VI A-B**). Note that the QRS complexes in the atrial unipolar signal generate spurious peaks in the absolute negative slope (ANS) signal, which is used to detect activations in further algorithm steps. Therefore, the ANS signal would be affected by such spurious peaks if QRS complexes are not properly removed. Those peaks are marked with red arrows in **Online Figure VI C**. In the PCA method, the QRS complexes from the unipolar traces are considered as realizations from stochastic processes for which a covariance matrix is derived. Then, the eigenvectors and eigenvalues of the covariance matrix are calculated, with the eigenvectors placed in one matrix *E* and the eigenvalues placed in a diagonal matrix *D*. From these two matrices, a whitening matrix (a matrix that transforms the original observation matrix *X* into a matrix of whitened principal components) and a dewhitening matrix (a matrix which undoes the whitening process) are derived:

Whitening Matrix: $W = D^{-1/2}E^T$

Dewhitening Matrix: $W^{-1} = ED^{1/2}$

The principal components can then be obtained from the observation matrix and whitening matrix by multiplication:

Principal Components: $P = WX$

These principal components were combined using their associated weights in the dewhitening matrix to create an individualized template for each QRS complex in the unipolar signal. Since the first principal components are assumed to express the ventricular activity because they express most of the variance from the original observations, QRS templates were created by combining the first 1-2 principal components, using their associated mixing variables from the dewhitening matrix W^{-1} (**Online Figure VI D**). These QRS templates were then subtracted from the original unipolar signal (**Online Figure VI E**).

Although this algorithm was quite effective, residual ventricular artifacts and their corresponding spurious activity in the ANS signal may remain (red intervals in **Online Figure VI F**). Therefore, a second novel algorithm using a surface ECG-lead, unipolar and bipolar signals was applied in order to discern whether the negative slope intervals during ventricular activation were ventricular far-field residues or true atrial activations. Thus, significant bipolar activity as a consequence of an atrial activation was detected in the bipolar signal. Intervals within the QRS windows previously used for the PCA algorithm (grey rectangles) that did not present significant bipolar activity were detected (green rectangles in **Online Figure VI G**). Then, the ANS signal was blanked during those green intervals since the negative slope activity present inside them was not considered as a true atrial activation but as residual ventricular far-field (red intervals in **Online Figure VI F**). Then, if present, true atrial activation times were detected on the previously 'blanked' ANS signal (**Online Figure VI H**) and translated into the corresponding unipolar signal after the QRS subtraction (**Online Figure VI I**). More details about the detection of activation times are described below. An example of the performance of these two complementary algorithms with ventricular activity of variable cycle length in patients with persistent AF is shown in **Online Figure VII**.

Computation of time-domain iFM and iAM

Online Figure VIII shows examples of instantaneous frequency modulation (iFM) computation from 2 unipolar signals (same pig presented in **Figure 3**). The signal displayed in panel A is from a region with

rotational activation, although with low hierarchy (median iFM: 5.3 Hz, 70th percentile). The signal displayed in panel B is from a region with high hierarchy (median iFM: 7.3 Hz, maximum value). Ablation of such a high hierarchy/leading-driver region terminated PersAF and rendered it non-sustainable. **Online Figure IX** describes in detail the algorithm to compute the instantaneous frequency modulation (iFM), instantaneous amplitude modulation (iAM) and rotational-footprint detection using as a representative example the unipolar signal displayed in **Online Figure VIII A** (row 1 in **Online Figure IX**). First, the slope (first derivative: dV/dt) of the unipolar signal was computed. Although intervals with positive slopes can provide useful information about the orientation of the electrical wavefront with respect of the recording unipolar electrode, activation times in unipolar action potentials are usually defined at the times of maximum negative slope within intrinsicoid deflections. Therefore, intervals in which the slope was positive were assigned a zero value in the first derivative. This way, a signal with peaks corresponding only to intervals of negative slope was obtained (absolute negative slope, ANS, measured in mV/ms, row 2 in **Online Figure IX**). This signal can be easily calculated as follows:

$$ANS(t) = \frac{1}{2}\left(\left|\frac{d\ Unipolar(t)}{dt}\right| - \frac{d\ Unipolar(t)}{dt}\right)(\frac{mV}{ms})$$

Then, the times when peaks in *ANS(t)* met the following criteria were selected as activation times: i) an amplitude above a minimum threshold (minimum slope), ii) a prominence above a certain value (minimum prominence), iii) an amplitude over the noise threshold, and iv) separated at least a minimum refractory period from the previous and the next ones.

The minimum slope (amplitude and prominence) was set as:

$$ANS_{min} = max\{0.03\ mV/ms,\ 0.05 \cdot P_{95th}(ANS)\ mV/ms\}$$

where the noise threshold for the ANS signal was set to 0.03 mV/ms. The 95th percentiles were used as reference instead the maximum values to prevent excessive thresholds due to artifactually high values in the unipolar signal slope. Importantly, to detect activations during intervals in which deflections are below the noise threshold, the algorithm lowers iteratively the noise threshold in steps of 0.005 mV until the algorithm detects physiological activations. If physiological activations are not found, the interval in which deflection amplitudes were below the noise threshold is blanked, so that such intervals do not influence the calculated parameters.

The minimum refractory period was set as follows:

$$RP_{min} = max\left\{50\ ms, \frac{1000}{1.95 \cdot min\{DF_{UNI}, DF_{ANS}, DF_{BIP}\}}\ ms\right\}$$

where DF_{UNI}, DF_{ANS} and DF_{BIP} are the dominant frequencies of the unipolar, ANS and bipolar signals respectively. DF_{UNI}, DF_{ANS} and DF_{BIP}were calculated as the frequencies with the highest peak in the power spectral density (PSD) of the *Unipolar(t), ANS(t)* and *Bipolar(t)* signals respectively. PSD was estimated by a conventional periodogram of the signal previously multiplied by a Kaiser window (β=2.5) and zero-padded to the next higher power of 2. All these dominant frequencies can be used as a rough surrogate of the reciprocal of the average interval between activations. The 1000 factor is used to convert that time into ms. Since activation intervals shorten and lengthen dynamically during fibrillation, the *1/1.95* factor is used to allow for intervals up to 1.95 times shorter than the surrogate average one [$(1/DF_{min})*1000$ ms]. This factor was chosen because it allows for activation intervals of almost half of the

surrogate average one (leaving room for considerable dynamic shortening), but at the same time it avoided considering double potentials as two consecutive activations. Values ≥2 would be highly problematic because double potentials would be considered as two consecutive activations which would bias the detection of activations. On the other hand, lower values would not leave much room for dynamic shortening of the AF cycle lengths. Note that in the first phase of activation detection there is not a high limit for the refractory period (the value 1.95 is not used for that purpose).

The calculated RP_{min} value usually provided a reasonable lower limit for the local refractory period. Nevertheless, if that value was lower than 50 ms, the algorithm used 50 ms as the minimum refractory period.[16, 25-27] It is worth noting that we chose a somehow conservative approach because this lower limit for the refractory period may miss some depolarizations when a phase singularity is close to the recording electrode/pixel. At the same time, the approach prevents oversensing of unspecific deflections related to wave collisions or depolarizations in the contralateral layer of the atria, etc, but unrelated to a driver. Anyway, rather than using a fixed value, our approach uses a changing minimum refractory period for each signal. It is calculated using several DF values (rough estimations of the activation rate) of the analyzed signals. The latter enables the algorithm to account for regional heterogeneities in activation rate. The 50 ms value is only used as a minimum refractory period. The resulting activation times are displayed as blue dots in row 4 of **Online Figure IX**. Note that, although the 50 ms limit seems to have a relevant role in determining the minimum refractory period, it is not as important as it first seems since the other value (calculated as $1000/(1.95*DF_{min})$) is usually higher than 50 ms. Indeed, for the result of such an operation to be 50 ms, the smallest DF should be >10.25 Hz:

$$\frac{1000}{1.95 \cdot \mathrm{DF_{min}}} < 50 \rightarrow \mathrm{DF_{min}} > 10.25\ \mathrm{Hz}$$

Therefore, the 50 ms value would be only used when extremely fast signals are mapped. The fastest AF we have seen was ~16 Hz (only in the LA) and was clearly an outlier (**Figure 6B**, the only pig in which ablation was not successful). Even in such a fast case, activations would be separated in average by 1000/16=62.5 ms which is higher than the 50 ms limit. In addition, the latter scenario is not usual, especially in human AF where is extremely rare to find signals with activation rates above 10 Hz.

In some specific subjects in which $min\{DF_{UNI}, DF_{ANS}, DF_{BIP}\}$ was erroneously low, the median value of the dominant frequencies was used instead:

$$RP_{min} = max\left\{50\ ms, \frac{1000}{1.95 \cdot median\{DF_{UNI}, DF_{ANS}, DF_{BIP}\}}\ ms\right\}$$

However, although such a lower limit for the refractory period can account for physiological dynamic changes in local activation intervals, it sometimes may result in too short, artificial intervals. Therefore, an iterative adaptive algorithm was used to refine activations allowing only physiological changes in atrial activation cycle lengths. This was done by 1) inserting potential activations within too long, non-physiological cycles if found, and/or 2) excluding potential activations when too short non-physiological cycles were found, and 3) adjusting potential activation times to the peak in the ANS signal that made inter-activation cycles change in a physiological not abrupt way when several, very close peaks of similar amplitude were present in the ANS signal. In our experience, this approach reliably accounted for the dynamic changes (shortening/lengthening) in AF cycle lengths in the sheep, pig and human data analyzed. In this specific signal no changes in activation times were performed by the adaptive iterative algorithm (**Online Figure IX**, row 5).

In addition, the amplitude of the intrinsicoid negative deflections in unipolar signals (surrogate of the phase-0 in optical action potentials) was calculated as the unipolar voltage excursion (mV) between the times at which the slope goes below the 4% of the slope at the corresponding activation time. The unipolar values at the activation times and their corresponding lower and upper amplitude limits for the intrinsicoid negative deflection are displayed as blue and red points respectively in row 6 of **Online Figure IX**. By interpolating such amplitude excursions, we obtained the unipolar envelope signal (*UE(t)*, **Online Figure IX**, row 7) as a surrogate of the optical envelope signal used in optical signals. Importantly, amplitudes during intervals with ventricular activation are interpolated from the neighboring ones because ventricular far-field could affect them, even after applying QRS minimization strategies. Since these amplitudes dynamically change as well (i.e. are modulated by several factors), their corresponding instantaneous amplitude modulation (iAM) signal (**Online Figure IX**, row 10, red trace) is calculated as follows:

$$iAM(t) = 100\left(1 - \frac{UE(t)}{\max\{UE(t)\}}\right)(\%)$$

Note that iAM is not an absolute measurement but a relative one. Thus, the lower the unipolar envelope normalized by its maximum value during the 8-second acquisition, the higher the iAM is. Intervals with low values of the iAM signal correspond to times when the unipolar envelope is unaffected, whereas intervals with high values of the iAM signal correspond to times when the unipolar envelope is highly affected by some factor/s (e.g. a drifting rotor is passing nearby, or the recording electrode is temporally far from the endocardium).

Also, a frequency modulated signal (*FM(t)*) was obtained by adjusting a sinusoidal wave in order to reach its maxima at the previously detected activation times (**Online Figure IX**, row 8). This way, the distribution of the FM sinusoidal cycles directly reflects the dynamic changes in tissue activation rate at any specific location.

In addition, a frequency- and amplitude-modulated (FM|AM) signal was obtained by multiplying the FM signal and the unipolar envelope signal (**Online Figure IX**, row 9):

$$FM|AM(t) = UE(t) \cdot FM(t)$$

Note that this FM|AM signal shows a similar morphology to optical signals. In addition to directly reflect the dynamic changes in tissue activation rate at any specific location in an intuitive way, this FM|AM signal clearly displays the dynamic changes in the amplitude of the unipolar intrinsicoid negative deflections.

Finally, the instantaneous frequency value between 2 consecutive activation times was calculated as the inverse of the interval between those activations in seconds. Analytically, given N consecutive activation times in ms ($t_0, t_1, \ldots, t_N$), the iFM(t) signal is calculated as follows:

$$iFM(t) = \sum_{n=0}^{N-1} \frac{1000}{t_{n+1} - t_n}\left[u(t - t_n) - u(t - t_{n+1})\right]$$

where $u(t)$ is the Heaviside unit step function, defined as $u(t) = \begin{cases} 0, t < 0 \\ 1, t \geq 0 \end{cases}$

Row 10 in **Online Figure IX** displays the resulting instantaneous frequency modulation (iFM) signal (blue trace).

Note that iAM tracks the relative or percent changes in unipolar negative deflection amplitudes regardless of the fact that such amplitudes are low (for example in fibrotic areas) or high (for example in atrial appendages). Therefore, low amplitudes should not constitute, per se, a drawback for the algorithm as shown in **Online Figure X**.

However, in regions with a high degree of fibrosis, signal fractionation may be present. Most literature about fractionation used bipolar signals acquired using ablation catheters with 3-4 mm length and 2.5 mm diameter electrodes. Bipolar configurations and especially those with poor spatial resolution (big electrodes) can result in increased fractionation,[28] especially in areas with a high degree of fibrosis. It is important to note that our algorithm mainly relies in unipolar signals acquired with tiny electrodes, which extremely reduces the degree of signal fractionation. An example of the different degrees of fractionation observed in 2 unipolar and the corresponding bipolar signal is shown in **Online Figure XI**. However, some fractionation may still be present in unipolar signals and influence the calculation of iFM, key to detect leading-driver regions. Such a calculation is mainly dependent on the algorithm used to detect atrial activations. When the algorithm was designed and tested, we took the maximum care in defining reasonable criteria that resulted in the detection of physiological activations even in the presence of fractionation. Note that if the refractory period set by the algorithm were too low, multiple deflections corresponding to the same activation or sequential activations in close cells would be detected as multiple activations in a short time interval, and therefore, it would result in high iFM values. However, we set the minimum refractory period between consecutive detected activations as described above, which combined with the subsequent iterative adaptive algorithm to refine activation times, results, in our experience, in a very physiological detection of activations even in the presence of fractionation. Examples of the algorithm performance with highly fractionated signals are shown in **Online Figure XII**. Note that despite a very high fractionation in the bipolar signal, the detection of activation times and calculation of iFM from the ANS signal is accurate.

To increase the quality of the signals used for the analysis, we discarded the signals acquired farther than 7 mm from the endocardial surface mesh generated with the electroanatomical mapping system and regardless of their morphology (standard approach in conventional electroanatomical mapping systems). In the remaining signals, and after ventricular far-field removal, the algorithm checked for every signal the percentage of time in which activations were not detected above the noise threshold in the ANS signal.

If such percentage was higher or equal than 60%, the algorithm then checked the distance at which the signal was acquired from the mesh surface:

- If such a distance was ≥3 mm, the registered low ANS values for long temporal periods (after ventricular far-field removal) was likely due to poor tissue-electrode contact. Thus, the signal is likely mostly composed of ventricular far-field and therefore discarded.

- If the signal was acquired near the surface mesh (<3 mm), the parameters measured from the signal were excluded from the maps because they may not be reliable, but the signal was not discarded. The absence of atrial voltages above the ANS noise threshold in the majority of these signals was due to the fact that were acquired in advanced positions inside the superior/inferior cava veins or pulmonary veins. Nevertheless, the few ones with these features and acquired from atrial myocardium (not veins), at least theoretically, might be due to the phase singularity of a meandering/drifting rotor staying very close to the electrode for long periods of time (>60% of the acquired 8 seconds). That is the reason why these

signals are not discarded, since they may make sense as islets of sustained ANS values below the noise threshold corresponding to rotor cores inside regions of higher signal quality.

However, it is important to note that purely stationary rotors may be only seen in very specific conditions (e.g. pharmacological K_{ATP} channel opening[9]). In more physiological conditions, most rotors are not stationary but drifting or meandering. Thus, the intervals of rotor-mediated low voltages are limited because it is not likely that the rotor stays exactly at the same location for 8 seconds. For this reason, a location that can be near the phase singularity at one specific time, can be far away at another time within the acquired 8 seconds, but still influenced by its rotational wavefront. Therefore, in practice, the number of signals with ANS values below the noise threshold for >60% of the acquisition time and acquired at low distance (<3 mm) from the endocardial mesh of atrial myocardium was negligible.

After this screening process to discard or exclude signals from analysis, the remaining signals were further analyzed and activations were detected as described above. Some examples are shown in **Online Figure XIII**.

Rotational-footprint detection

Single-signal detection of a rotational-footprint using electrical unipolar signals was performed identically as it was performed for optical signals. Note that there is no way to adjust the parameter combination to be optimal for an individual *in vivo* experiment since we do not have a 'gold-standard' *in vivo* with which we can obtain sensitivity and specificity. As discussed in the main manuscript, activation/phase mapping of electrical data cannot be considered a 'gold-standard' for multiple technical reasons. Thus, we only used electrical phase movies to qualitatively assess the correlation between the rotational cores in such movies and the rotational-footprint positive locations detected by the iFM/iAM algorithm within areas where the PentaRay catheter was fully-deployed (see **Figure 3B** and **Online Movies III-V**). However, we used *ex vivo* high-quality optical mapping movies (which are considered the 'gold-standard') to obtain the optimal parameter combinations and to properly validate the iFM/iAM algorithm to detect rotational-footprints. Thus, for *in vivo* experiments, we used the *ex vivo* parameter combination that maximized sensitivity as long as specificity was ≥97.5% with a 2.5 mm tolerance (note that this tolerance is considerably smaller than the actual lesions created with a conventional ablation catheter[19]). Therefore, we prioritized specificity over sensitivity in order to minimize false positives for rotational-footprint detection *in vivo*. Specifically, such a combination provided 92.6 (4.3) % average sensitivity and 97.5 (2.3) % average specificity. The following parameters were used to achieve such values of sensitivity and specificity:

parameter1= 4 (minimum number of increasing iFM cycles)

parameter2= 25 % (minimum iAM excursion)

parameter3= 3 (minimum number of increasing iAM cycles)

parameter4= 85 % (iAM threshold)

parameter5= 70th percentile (minimum iFM percentile without increasing iFM together with iAM ≥ parameter4 to consider a quasi-stationary / meandering in the surroundings rotor footprint)

Thus, and considering the 2 main parameters, to detect the footprint of a drifting rotor core in vivo with a maximum location error of 2.5 mm, the algorithm requires *i)* that the iFM increases for at least 4 cycles (i.e. cycle length progressively shortens for at least 4 cycles), and *ii)* the iAM increases and goes over 85%

(i.e. the amplitude of unipolar negative deflections decreases below the 15% of the maximum unipolar negative deflection in the analyzed signal). Note that a minimum increasing number of iFM cycles in the range of 3-5 and iAM thresholds between 75% and 95% could be considered physiologically reasonable. Therefore, the used values *in vivo* make sense from a physiological point of view. The fact that the minimum increasing number of iFM cycles is 4 (and not 3, 5, etc.), and that the iAM threshold is 85% (and not 80%, 90%, etc.), is that 4 and 85 were the exact values that maximized sensitivity as long as specificity was ≥ 97.5% with a 2.5-mm tolerance. Also, note that although a few rotational-footprints may have been missed in our analysis of in vivo data (sensitivity: 92.6 (4.3) %), very few false rotational-footprint negatives should be present (specificity: 97.5 (2.3) %). Some examples are shown in **Figures 3**, **6**, **7**, **8** and **Online Figures IX**, **XIV** and **XXIII-XXV**.

Note that this algorithm enables *in vivo* detection of individual locations with a rotational footprint without the need of performing phase or activation mapping of multiple electrograms simultaneously acquired using costly panoramic acquisition techniques (basket catheters[29] /electrode vests[30]) or fully deployed multispline catheters.[31] Since single-signals are used, no adjustment is needed in the algorithm regardless of the spatial resolution of the method used to map AF and despite the fact that optical and electrical mapping have completely different spatial resolutions. Besides, optical and electrical mapping have temporal resolutions in the same range (fs=600 Hz, ts=1/600=1.67 ms in our optical mapping movies; fs=1000 Hz, ts=1/1000=1 ms in our electrical signals) that are sufficient to appropriately sample the activation-repolarization phenomena and capture the changes in frequency and amplitude of both optical and electrical signals. Thus, the changes in phase 0 (in optical signals) or intrinsicoid negative deflections (in unipolar electrical signals) will be properly captured in order to calculate the iAM and iFM signals. Therefore, the parameters of the algorithm do not need to be adjusted due to differences in spatio-temporal resolutions between optical and electrical mapping.

Worthy of note is the fact that the signal in one optical pixel/electrode represents an integration of signals from cells with different activation times. The latter may result in integrated and artificial optical upstrokes/electrical deflections. This effect would be especially relevant near phase singularities of rotational activations due to decreased conduction velocity. The spatial resolution of our optical mapping experiments was ~0.7 mm/pixel and we used 1-mm electrodes to acquire electrical signals. Therefore, this effect, if present, should be similar in both scenarios. Therefore, such an issue should not affect the sensitivity and specificity of the algorithm when applied to electrical signals. However, it might affect to the detection of activation times, and hence the iFM, in signals very close to rotor cores. Analyzing the signals close to rotor cores is always challenging since they are of extremely low amplitude. Indeed, in some studies these signals were ignored (e.g. see figure 5 in reference[26]). However, at a small distance from the rotor core, cells in one pixel/electrode should be activating almost synchronically, and therefore, such an issue should not be problematic. Indeed, even though the detection of activation times was not accurate in the close vicinity of rotor cores, since ablation lesions have usually diameters ≥3 mm,[19] it is very likely that an ablation lesion delivered not at a rotor core but close to it, would also encompass the rotor core. Anyway, we do not think that ablating the very rotor core would be enough to prevent future episodes of AF. Indeed, this kind of lesion could provide a potential anatomical center of rotation to sustain reentry within the high iFM region. Indeed, most rotors are drifting, and therefore, ablation strategies should be focused on limiting the region capable of sustaining activity with high iFM, either by creating lines inside them that reach anatomical obstacles,[31] or by performing coin-like sets of lesions.

Data interpolation on 3D maps

Similarly to what is done in conventional electroanatomical mapping systems, we used a modified inverse distance weighting algorithm to interpolate data values in points of the 3D surface mesh from the known values at scattered non-regularly distributed spatial locations (the ones where catheter electrodes were actually located during acquisition). The assigned values for a function f in an unknown point (x, y, z) in the 3D mesh were calculated as a weighted average of the values available at the *N* truly acquired points (x_i, y_i, z_i) within a distance *D* from (x, y, z):

$$f(x,y,z) = \begin{cases} \frac{\sum_{i=1}^{N} w_i(x,y,z) \cdot f(x_i,y_i,z_i)}{\sum_{i=1}^{N} w_i(x,y,z)}, & if\ \forall i \in \{1,\dots,N\} \mid d\{(x,y,z),(x_i,y_i,z_i)\} \neq 0 \\ f(x_i,y_i,z_i), & if\ \exists i \in \{1,\dots,N\} \mid d\{(x,y,z),(x_i,y_i,z_i)\} = 0 \end{cases}$$

The weighting value $w_i(x, y, z)$ was proportional to the inverse of the distance to each known point raised to a *p* power:

$$w_i(x,y,z) = \frac{1}{(d\{(x,y,z),(x_i,y_i,z_i)\})^p}\ , i \in \{1,\dots,N\}$$

where $d\{(x, y, z), (x_i, y_i, z_i)\}$ is the distance between the point at which we are obtaining the f function interpolated value and the point at which the function f has a known value. For 3D median/mean iFM maps, bipolar voltage maps and dominant frequency maps, the following parameters were used: *D*=7 mm, *p*=2. For 3D phase movies (see next section) *p*=1 was used instead. Note that in the truly acquired points, there is no interpolation since the actual acquired value is assigned to the mesh for that location. Since rotational-footprint is a binary function (positive=1/negative=0), a 2.5 mm distance criterion (the tolerance of the iFM/iAM algorithm) was applied to interpolate such values to the nearest neighbors from the actual acquired points (x_i, y_i, z_i). An example is shown in **Online Figure XIV** (left column).

Phase Mapping of in-vivo electrical data

We obtained phase movies using the electrical data provided by the 20 poles of the PentaRay catheter. Note that the detection of rotational activation using phase mapping of electrical data requires multiple electrodes and is completely dependent on their location and separation.[4-6] Also, depending on the signal processing performed, phase mapping of electrical signals could be little specific for rotor detection.[4, 6, 32] Therefore, we could only expect reliable phase movies when the PentaRay catheter was well deployed and attached to the tissue.[5] However, this scenario is not achievable in important areas of the atria.[31] Even so, some difficultly predictable issues can arise due to interpolation of sparse electrode data.[7] Note that all these major drawbacks are not present in phase movies obtained from optical mapping due to a large number of signals acquired (6400 in our optical movies) and the high spatial resolution (0.43-0.62 mm in our optical movies). Therefore, unlike phase mapping of optical data, phase mapping of electrical data cannot be considered as a *gold-standard*. Thus, we only used these phase movies of electrical data to qualitatively assess the correlation between the rotational cores in these phase movies obtained from electrical data and the rotational-footprint positive locations detected by the iFM/iAM algorithm in areas where the PentaRay catheter was fully deployed (see **Figure 3B** and **Online Movies III-IV**).

To obtain phases we performed Hilbert transformation (HT) of the frequency modulated (FM) signals from the 20 PentaRay electrodes $(FM_i(t), i = 1, \dots, 20)$. Since such signals are sinusoids that reflect the changes

in AF intervals rather than raw unipolar potentials, their morphology is more convenient for phase transformations.[33] Thus,

$$\varphi_n(t) = phase[FM_n(t) + jHT\{FM_n(t)\}], \qquad n = 1, \dots, 20$$

where

$$HT\{FM_n(t)\} = \frac{1}{\pi} \int_{-\infty}^{+\infty} \frac{FM_n(\tau)}{t - \tau} d\tau$$

Upon computation of the phase signal from the 20 electrodes, complex vectors $e^{j\varphi_n(t)}$ whose scalar phases were $\varphi_n(t)$, were also obtained. We used these complex vectors to obtain, by means of interpolation, the corresponding complex vectors in all the spatial locations of the 3D surface mesh covered by the deployed PentaRay. Of note, we did not directly interpolate the scalar phases $\varphi_n(t)$ (between $-\pi$ and $+\pi$),[34, 35] but their associated complex vectors $e^{j\varphi_i(t)}$, because the first approach would result in phase maps without clear activation fronts.[36] Finally, the scalar phases (between $-\pi$ and $+\pi$) for every point in the 3D surface mesh were retrieved from the interpolated complex vectors. Phase movies display the changes in the scalar phases at each location of the surface mesh for every time point (see **Online Movies III** and **IV**).

QRST complex removal and Dominant Frequency calculation of surface ECG tracings

If ablation did not achieve AF termination, the atrial frequency content of 12-lead surface ECG tracings was compared before and after ablation to discern whether ablation effectively modified the AF substrate. To do so, ventricular components (QRST complexes) were removed from the surface ECG before calculating DF. To estimate the ventricular subtracting signals, we used the same PCA method described above. See **Online Figures XV-XVI** for sample representations of this methodology.

SUPPLEMENTAL DISCUSSION

Additional technical considerations

Median iFM maps may look similar to dominant frequency (DF) maps used in previous mechanistic approaches to treat PersAF that reported suboptimal results.[37] However, despite DF and median iFM approaches try to summarize the frequency content of a signal in one number, important conceptual differences are present between both:

First, DF is a spectral measurement (obtained from the frequency-domain) and is a good surrogate of the activation rate in optical mapping recordings or for good quality and quite regular in amplitude and frequency electrical signals.[38-40] Otherwise, DF interpretation may be challenging due to the presence of multiple spectral peaks of similar height.[38-40] Moreover, the time intervals with the highest iFM values usually show the lowest amplitudes and vice versa, which conditions the height of their corresponding power spectral peaks, and hence, the selected DF. Unlike DF, median iFM is derived from time-domain measurements that indeed seem to be more robust than spectral measurements for a wider range of electrical signals after applying properly designed algorithms.[16]

Second, iFM provides very useful information for detecting rotational-footprints in single signals. Furthermore, iFM_{median} maps can be displayed in combination with the location of rotational footprints. However, such information cannot be obtained using DF analysis.

Third, since iFM tracks dynamic changes in the local activation rate during acquisition, it provides additional advantages compared with DF. For example, it potentially enables to automatically detect regions with transient bursts of focal activity (3D intramural reentry or not) that could contribute to (re)initiate or maintain AF. Simple comparison of 50th (median) and 90th percentiles of iFM would help on such analysis. However, DF analysis is not sensitive to rapid dynamic variations of the activation rate during the acquisition interval.

We further analyzed a sample pig with persistent AF using the iFM/iAM algorithm to detect leading-driver regions, and using the previously reported DF mapping approach.[37, 41] We obtained DF maps from the 8-second bipolar signals at each location. Briefly, power spectral density was obtained via Welch's periodogram (Hanning window)[37, 41] using Matlab scripts (Mathworks Inc., Natwick, USA). At each spatial location, a DF value was assigned based on the highest peak in the power spectral density within a 3-20 Hz range. The power spectrum was also used to quantify the aperiodic complexity of the signals using the regularity index (RI), which varies between 0 (completely aperiodic) and 1 (completely periodic). RI was defined as the ratio of the power at the DF and adjacent frequencies (≈0.75 Hz band)[37, 41] to the sum of the power in the whole analyzed frequency range. This index is based on the fact that the purest signal in the frequency domain is a sinusoidal with infinite duration, which should have a single spectral line (Dirac delta, no spectral width) and therefore its RI should be 1. Since the analyzed signals do not have an infinite duration, even the spectrum calculated from a sinusoid will have some width due to the temporal windowing produced by their limited length. This justifies that a certain frequency margin around the DF is considered when the RI is calculated.

Although by definition DF should be the frequency corresponding to the highest peak in the power spectral density, this may return DF values as high as 20 Hz which is not a physiological value in this scenario. Therefore, we established 16 Hz as the upper limit for DF, since, although rare, 16 Hz is the highest

activation rate we have seen in pigs with persistent AF *in vivo* (see **Figure 6B**). However, harmonic peaks (at double, triple, etc. frequencies of the fundamental one) makes this approach little reliable with bipolar electrical signals since the second harmonic is many times selected as DF because is the highest peak in the analyzed band.[42] Therefore, we also tested how the bipolar DF map would be after setting an upper limit of only 8 Hz. Finally, since disorganized signals can display artifactual DF values, we also calculated the bipolar DF map after discarding signals with RI<0.2, as previously reported.[37, 41] While this measure can theoretically help to clean the bipolar DF map by removing low quality or very irregular signals (although is not clear that irregular signals deserve to be ignored),[43] it also eliminates very good signals that display a RI<0.2 due to their markedly harmonic spectrum.

As a representative example, **Online Figure XXVII** shows a comparison of the following maps: 1) Median iFM, 2) Mean iFM, 3) Bipolar DF (upper limit for DF: 16 Hz), 4) Bipolar DF (upper limit for DF: 8 Hz) and 5) Bipolar DF (upper limit for DF: 8 Hz, only points with RI≥0.2). Maps 1 and 2 (median and mean iFM) are virtually identical. Indeed, the correlation between median and mean iFM values at the acquired locations was extremely high (R^2=0.94, p<0.0001, n=3204 points) which is a criterion for converge in some iterative algorithms.[16]

When the upper limit for DF maps was set at 16 Hz, the bipolar DF map did not show any global correlation with the median iFM map (R^2=0). Although a local good/acceptable correlation is achieved for some points (see **Online Figure XXVIII a-c**), the correlation plot shows that most DF values were much higher that the corresponding median iFM values. Many of these wrong values are related to selection of the second harmonic as DF (points around the red line in the correlation plots) or simply because of the existence of too many peaks of similar height in the spectrum. Examples of wrongly selected bipolar DF values are shown in **Online Figure XXVIII d-i**.

When the upper limit for DF maps was set at 8 Hz, the DF map was slightly more similar to the median iFM map. However, the global correlation was still practically zero (R^2=0.01). Note that the leading driver area in purple in the median iFM map also yielded high bipolar DF values (**Online Figure XXVII**). However, there were many more areas with similar DF values (LIPV, RIPV, RSPV, ICV, SCV, free wall of the RA), which made the DF map unspecific. Note that after limiting the upper threshold for DF maps to 8 Hz, signals d-i presented more physiological DF values, but even so several of them showed a poor correlation with median iFM.

In an attempt to increase the specificity of the DF map, points with RI<0.2 were removed as previously reported.[37, 41] This considerably increased the correlation between bipolar DF values and median iFM (R^2=0.597, p<0.0001, n=147 points). However, >95% of points were removed and only 147 out of 3204 points remained. For example, signal *b* whose DF value was very similar to the median iFM value, was removed because its RI was 0.18. This was because the harmonic peaks increased the value of denominator but not numerator in the RI ratio, resulting in low RI values even when the signal was very regular.[44] Note that only 2 out of the 9 sample signals showed in **Online Figure XXVIII** presented a RI≥0.2 (*a* and *c*). That yielded a map with large atrial areas without information (dark gray areas). These areas were not even larger because of the interpolation methods used in 3D maps. In fact, although points *b* and *d-i* were removed because of RI<0.2, they are located inside colored regions that are the result of interpolation from the sparse neighboring points that remained in the map.

Long-term follow-up in patients

To complete our translational endeavor, we tested that the presented approach could be safely translated to a small cohort of patients and that it may provide potential value, either to successfully treat the arrhythmia, or to identify the patients in whom a limited catheter ablation is unlikely to provide beneficial effects. For example, ablation outcomes in a patient with a 5-month PersAF episode were similar to those in pig procedures (AF termination and non-sustainability after ~10 min radiofrequency delivery, patient #2 of the interventional group, **Online Table X**, **Figure 8B**). However, AF termination may not always acutely occur after targeting driver regions. Nevertheless, the fact that patients remain in sinus rhythm after 16 months suggests that the AF substrate was effectively modified.[31] We observed such a scenario after targeting a leading-driver region next to the antrum of a reconnected PV (patient #3 of the interventional group, **Online Table X**). No recurrences were documented in the aforementioned 2 patients after a 16-month follow-up period without antiarrhythmic drugs. Conversely, long-standing PersAF episodes (e.g. 12-month episode in patient #1 of the interventional group **Online Table X**, **Figure 8A**) or subject-specific atrial functional properties (e.g. pig case showed in **Figure 6B**) may result in large areas of atrial remodeling that potentially generate too large and fast driver regions that make catheter-based ablation prone to fail. Therefore, and although more testing is needed in patients, we surmise that this kind of maps, with large proportions of highly remodeled atrial tissue capable of sustaining high iFM activity, may anticipate the failure of a limited catheter ablation to acutely terminate AF and to maintain the patient in sinus rhythm. Conversely, maps with clearly delimited 'islets' of high iFM_{median} may predict better acute and long-term outcomes after limited catheter ablation.

Online Table I. iFM/iAM algorithm to detect rotational-footprints: tested combinations

Total persistent AF optical movies	125						
Excluded persistent AF optical movies [ʃ]	8						
Analyzed persistent AF optical movies	117						
Signals per movie [‡]	5970 ± 150						
Total signals	698,456						
**Parameters**							
Min. number of increasing iFM cycles (parameter_1)	0 (disabled)	2	3	4	5	6	7
Min. number of increasing iAM cycles (parameter_3)	0 (disabled)	1	2	3	4		
iAM threshold (%) (parameter_4)	0 (disabled)	75	80	85	90	95	
Min. iAM excursion (%) (parameter_2)	0 (disabled)	5	15	25	35	45	
Min. iFM percentile [µ] *(parameter_5)*	70	75	80	85	90	95	100 (disabled)
Tested parameter combinations per movie: 8820							
Minimum refractory period (ms)	constant: 50		signal-specific: max{50, 1/[1.95 min{DF_{OAP},DF_{APS}}]}				
All criteria met for ≥	1 cycle	2 cycles	3 cycles	4 cycles	5 cycles		
Tolerances (mm)	0	1.25	2.5				
Total data registers per movie	264,600						
Total data registers	30,958,200						

APS: absolute positive slope, DF: dominant frequency, iAM: instantaneous Amplitude Modulation, iFM: instantaneous Frequency Modulation, OAP: optical action potential

ʃ Exclusion based on suboptimal quality (poor signal-to-noise ratio, lighting artifacts, etc.)

‡ Camera resolution= 80x80= 6400 pixels but some corner areas were masked due to poor lighting or absence of atrial tissue

µ Min iFM percentile without increasing iFM together with iAM ≥ iAM threshold to consider a quasi-stationary / meandering rotor footprint

Online Table II. iFM/iAM algorithm to detect rotational-footprints: optimal parameters

	Tolerance	# Parameters	iAM threshold (%) *(parameter_4)*	Min. number of increasing iFM cycles *(parameter_1)*	Min. number of increasing iAM cycles *(parameter_3)*	Min. iFM percentile[µ] *(parameter_5)*	Min. iAM excursion (%) *(parameter_2)*	**Sensitivity (%) ʃ**	**Specificity (%)**	**Sum (%) ʃ**
Maximization of the sum of Sensitivity + Specificity	0 mm	1[ʒ]	85					85.5 (12.1)	61.6 (11.6)	147.0 (10.4)
		1		4				87.7 (7.3)	33.1 (11.2)	121.4 (6.9)
		2	80	3				71.1 (12.1)	69.1 (9.1)	140.3 (7.8)
		3	80	3	2			70.3 (10.0)	72.0 (10.2)	141.8 (6.8)
		4	80	3	2	70		77.1 (9.4)	69.8 (10.7)	146.4 (6.9)
		5	80	3	2	70	15	77.0 (9.4)	70.0 (10.6)	146.4 (6.8)
	1.25 mm*	1[ʒ]	90					94.5 (5.5)	82.9 (12.1)	177.8 (12.7)
		1		5				93.0 (5.8)	70.9 (12.5)	164.3 (12.8)
		2	85	3				92.2 (6.4)	87.7 (10.6)	180.3 (12.4)
		3	85	3	2			91.8 (5.5)	90.6 (6.2)	182.2 (6.6)
		4	80	4	2	70		93.2 (3.9)	90.4 (5.0)	183.4 (4.6)
		5	80	4	3	70	25	93.1 (3.9)	90.6 (4.9)	183.5 (4.5)
	2.50 mm‡	1[ʒ]	90					98.2 (2.4)	85.5 (13.2)	184.1 (12.5)
		1		5				98.5 (2.1)	72.1 (13.4)	171.0 (12.9)
		2	80	4				95.4 (4.1)	93.3 (7.0)	189.1 (6.3)
		3	85	3	2			97.2 (3.0)	93.3 (6.2)	190.4 (6.4)
		4	80	4	3	70		97.1 (2.4)	94.8 (3.5)	191.9 (4.0)
		5	80	4	3	70	35	97.0 (2.6)	95.1 (3.4)	192.0 (4.0)
Maximization of Sensitivity with Specificity ≥ 95%	0 mm	1[ʒ]	N/A					N/A	N/A	N/A
		1		N/A				N/A	N/A	N/A
		2	N/A	N/A				N/A	N/A	N/A
		3	N/A	N/A	N/A			N/A	N/A	N/A
		4	90	4	3	70		21.3 (7.0)	95.0 (2.8)	116.3 (5.6)
		5	90	4	3	70	15	21.3 (7.0)	95.0 (2.8)	116.2 (5.6)
	1.25 mm*	1[ʒ]	N/A					N/A	N/A	N/A
		1		7				23.4 (14.4)	97.2 (3.5)	120.6 (12.2)
		2	90	4				63.0 (11.0)	95.8 (6.5)	159.3 (10.25)
		3	85	4	2			76.7 (8.6)	95.4 (3.6)	172.0 (8.2)
		4	80	5	3	80		82.6 (7.6)	95.5 (2.7)	177.9 (6.9)
		5	80	5	2	80	35	83.9 (7.3)	95.1 (2.8)	178.9 (6.7)
	2.50 mm‡	1[ʒ]	N/A					N/A	N/A	N/A
		1		7				39.6 (18.1)	97.3 (3.8)	137.0 (16.2)
		2	85	4				91.5 (5.9)	95.5 (7.0)	187.5 (7.0)
		3	85	3	3			94.2 (4.1)	95.7 (4.1)	189.8 (5.3)
		4	80	4	3	80		96.8 (2.6)	95.1 (3.3)	191.9 (4.0)
		5	80	4	3	70	35	97.0 (2.6)	95.1 (3.3)	192.0 (4.0)
Maximization of Specificity with Sensitivity ≥ 95%	0 mm	1[ʒ]	N/A					N/A	N/A	N/A
		1		N/A				N/A	N/A	N/A
		2	N/A	N/A				N/A	N/A	N/A
		3	N/A	N/A	N/A			N/A	N/A	N/A
		4	N/A	N/A	N/A	N/A		N/A	N/A	N/A
		5	N/A	N/A	N/A	N/A	N/A	N/A	N/A	N/A
	1.25 mm*	1[ʒ]	85					97.6 (3.1)	76.1 (12.4)	173.9 (11.6)
		1		4				99.9 (0.2)	43.3 (12.1)	144.0 (11.2)
		2	80	3				96.4 (3.9)	81.4 (11.0)	178.1 (9.1)
		3	80	3	2			95.9 (3.5)	84.5 (7.5)	180.1 (6.7)
		4	75	4	3	70		95.6 (3.1)	86.3 (6.1)	181.7 (5.5)
		5	75	4	3	70	45	95.1 (3.3)	87.1 (6.0)	182.0 (5.3)
	2.50 mm‡	1[ʒ]	90					98.2 (2.4)	85.5 (13.2)	184.1 (12.5)
		1		5				98.5 (2.1)	72.1 (13.4)	171.0 (13.0)
		2	80	4				95.4 (4.1)	93.3 (7.0)	189.1 (6.3)
		3	85	3	2			97.2 (3.0)	93.3 (6.2)	190.4 (6.4)
		4	80	5	2	70		95.4 (4.1)	96.4 (2.6)	191.7 (4.4)
		5	80	5	2	70	35	95.2 (4.1)	96.6 (2.6)	191.7 (4.5)

Data are expressed as Mean (SD)

iAM: instantaneous Amplitude Modulation, iFM: instantaneous Frequency Modulation

ʒ Note that this parameter standalone would not be specific enough used with electrical signals acquired in vivo due to the amplitude modulation produced by the changes in electrode-tissue contact due to catheter or respiratory displacement, ventricular contraction, etc.

* Radius of a conventional ablation catheter

‡ Diameter of a conventional ablation catheter

µ Min iFM percentile without increasing iFM together with iAM ≥ iAM threshold to consider a quasi-stationary / meandering rotor footprint

ʃ Two movies without true positives were not included for these descriptive statistics since sensitivity could not be calculated

Online Table III. Optimal parameter combinations for single-signal detection of rotational footprints in optical mapping movies of PersAF (N=117) using either constant or signal-specific minimum refractory periods

Tolerance	Signal Type	RP_{min} (ms)	iAM threshold (%)	Min. number of increasing iFM cycles	Min. number of increasing iAM cycles	Min. iFM percentile[µ]	Min. iAM excursion (%)	Sensitivity (%)	Specificity (%)	Sum (%)
1.25 mm*	Optical	50	85	4	3	70	25	96.7 [93.6-98.9]	81.3 [69.5-90.2]	175.6 [166.9-181.5]
		$\max\{50, 1/[1.95 \min\{DF_{OAP}, DF_{APS}\}]\}$	80	4	3	70	25	93.9 [91.0-96.3]	91.1 [87.96-94.1]	184.3 [180.9-186.4]
2.5 mm‡	Optical	50	85	4	3	70	25	99.5 [97.9-99.9]	86.9 [75.5-93.6]	185.1 [174.2-191.2]
		$\max\{50, 1/[1.95 \min\{DF_{OAP}, DF_{APS}\}]\}$	80	4	3	70	35	97.9 [96.2-98.9]	95.5 [92.7-97.7]	192.6 [189.2-194.9]

Data are expressed as Median [P25th-P75th]

iAM: instantaneous Amplitude Modulation, iFM: instantaneous Frequency Modulation

Color legend: Median ≥ 90% / 70% ≤ Median < 90% / Median < 70%

* Radius of a conventional ablation catheter

‡ Diameter of a conventional ablation catheter

µ Min iFM percentile without increasing iFM together with iAM ≥ iAM threshold to consider a quasi-stationary / meandering rotor footprint

APS: absolute positive slope

DF: dominant frequency

OAP: optical action potential

RP: refractory period

Online Table IV. Performance of the experimentally derived parameters of the iFM/iAM algorithm for single-signal detection of the rotational footprints produced by quasi-stationary rotors in computer simulations of PersAF

Tolerance	Signal Type	RP_{min} (ms)	iAM threshold (%)	Min. number of increasing iFM cycles	Min. number of increasing iAM cycles	Min. iFM percentile[µ]	Min. iAM excursion (%)	Sensitivity (%)	Specificity (%)	Sum (%)
1.25 mm*	Transmembrane	50	80	4	3	70	25	93.8 [83.5-95.0]	100 [100-100]	193.6 [183.5-195.0]
		$\max\{50, 1/[1.95 \min\{DF_{AP}, DF_{APS}\}]\}$						39.7 [37.2-41.3]	100 [100-100]	139.7 [137.2-141.3]
	Unipolar pixel	50						89.5 [81.8-90.7]	97.4 [96.2-98.3]	186.7 [178.8-187.7]
		$\max\{50, 1/[1.95 \min\{DF_{UNI}, DF_{ANS}, DF_{BIP}\}]\}$						57.6 [48.9-64.2]	97.4 [96.2-98.4]	154.1 [146.1-161.5]
	Unipolar electrode	50						87.7 [80.9-89.4]	98.1 [97.6-98.4]	185.8 [178.9-187.4]
		$\max\{50, 1/[1.95 \min\{DF_{UNI}, DF_{ANS}, DF_{BIP}\}]\}$						51.6 [48.9-56.4]	98.1 [97.6-98.6]	150.0 [146.7-154.5]
2.5 mm‡	Transmembrane	50	80	4	3	70	25	97.3 [96.7-98.6]	100 [100-100]	197.3 [196.7-198.6]
		$\max\{50, 1/[1.95 \min\{DF_{AP}, DF_{APS}\}]\}$						52.7 [50.5-63.4]	100 [100-100]	152.7 [150.5-163.4]
	Unipolar pixel	50						96.6 [93.6-97.4]	97.3 [96.0-98.3]	193.4 [191.5-194.8]
		$\max\{50, 1/[1.95 \min\{DF_{UNI}, DF_{ANS}, DF_{BIP}\}]\}$						81.3 [74.9-82.6]	97.4 [96.1-98.4]	177.3 [172.8-180.1]
	Unipolar electrode	50						96.1 [94.1-97.6]	98.0 [97.5-98.5]	194.5 [192.1-195.2]
		$\max\{50, 1/[1.95 \min\{DF_{UNI}, DF_{ANS}, DF_{BIP}\}]\}$						71.1 [68.4-76.6]	98.2 [97.6-98.7]	169.4 [166.2-174.6]

Data are expressed as Median [P25th-P75th], N= 9 computer simulations of PersAF (see text for details)

iAM: instantaneous Amplitude Modulation, iFM: instantaneous Frequency Modulation

Color legend: Median ≥ 90% / 70% ≤ Median < 90% / Median < 70%

* Radius of a conventional ablation catheter

‡ Diameter of a conventional ablation catheter

µ Min iFM percentile without increasing iFM together with iAM ≥ iAM threshold to consider a quasi-stationary / meandering rotor footprint

AP: action potential

APS: absolute positive slope

ANS: absolute negative slope

BIP: bipolar

DF: dominant frequency

RP: refractory period

UNI: unipolar

Unipolar electrode of 1 mm^2

Online Table V. Optimal parameter combinations for single-signal detection of the rotational footprints produced by quasi-stationary rotors in computer simulations of PersAF

Tolerance	Signal Type	RP_{min} (ms)	iAM threshold (%)	Min. number of increasing iFM cycles	Min. number of increasing iAM cycles	Min. iFM percentile$^{\mu}$	Min. iAM excursion (%)	Sensitivity (%)	Specificity (%)	Sum (%)
1.25 mm*	Transmembrane	50	75	3	2	70	0	96.3 [91.2-98.2]	100 [100-100]	196.3 [191.3-191.2]
		max{50, 1/[1.95 min{DF_{AP},DF_{APS}}]}	75	3	2	70	0	80.5 [78.8-81.5]	100 [100-100]	180.5 [178.8-181.3]
	Unipolar pixel	50	85	3	2	70	25	92.5 [87.2-94.0]	96.7 [96.5-97.6]	189.9 [182.4-190.9]
		max{50, 1/[1.95 min{DF_{UNI},DF_{ANS},DF_{BIP}}]}	80	3	2	70	0	82.0 [81.0-86.4]	90.4 [89.9-93.3]	175.1 [171.1-176.1]
	Unipolar electrode	50	85	3	2	70	25	92.0 [87.6-93.1]	97.5 [97.4-98.1]	189.9 [184.3-190.6]
		max{50, 1/[1.95 min{DF_{UNI},DF_{ANS},DF_{BIP}}]}	75	3	2	70	25	89.3 [88.1-90.8]	84.5 [83.8-87.1]	174.9 [173.8-176.3]
2.5 mm‡	Transmembrane	50	75	3	2	70	0	99.5 [98.3-99.8]	100 [100-100]	199.4 [198.3-199.8]
		max{50, 1/[1.95 min{DF_{AP},DF_{APS}}]}	75	3	2	70	0	93.8 [90.7-94.8]	100 [100-100]	193.8 [190.6-194.8]
	Unipolar pixel	50	85	3	3	70	25	96.5 [94.1-97.3]	98.3 [98.1-98.7]	195.1 [191.3-195.6]
		max{50, 1/[1.95 min{DF_{UNI},DF_{ANS},DF_{BIP}}]}	80	3	3	70	25	90.7 [86.2-92.7]	94.9 [94.3-96.4]	186.1 [179.5-187.3]
	Unipolar electrode	50	85	3	2	70	25	98.0 [96.2-98.9]	97.4 [97.3-98.1]	195.7 [193.1-196.2]
		max{50, 1/[1.95 min{DF_{UNI},DF_{ANS},DF_{BIP}}]}	75	3	3	70	25	94.3 [93.3-95.3]	90.8 [90.0-92.5]	186.1 [181.2-186.6]

Data are expressed as Median [P25th-P75th], N= 9 computer simulations of PersAF (see text for details)
iAM: instantaneous Amplitude Modulation, iFM: instantaneous Frequency Modulation
* Radius of a conventional ablation catheter
‡ Diameter of a conventional ablation catheter
µ Min iFM percentile without increasing iFM together with iAM ≥ iAM threshold to consider a quasi-stationary / meandering rotor footprint
AP: action potential
APS: absolute positive slope
ANS: absolute negative slope
BIP: bipolar
DF: dominant frequency
RP: refractory period
UNI: unipolar
Unipolar electrode of 1 mm^2

Color legend: Median ≥ 90% ; 70% ≤ Median < 90% ; Median < 70%

Online Table VI. Performance of the optimal parameters for quasi-stationary rotor detection in computer simulations applied to experimental optical mapping data

Tolerance	RP_{min} (ms)	iAM threshold (%)	Min. number of increasing iFM cycles	Min. number of increasing iAM cycles	Min. iFM percentile$^{\mu}$	Min. iAM excursion (%)	Combination optimal for	Used with	Sensitivity (%)	Specificity (%)	Sum (%)
1.25 mm*	50	75	3	2	70	0	Simulated action potentials	Experimental optical action potentials	99.9 [99.6-100]	38.3 [24.0-52.9]	138.3 [123.1-152.2]
	max{50, 1/[1.95 min{DF_{AP},DF_{APS}}]}	75	3	2	70	0			99.2 [98.1-99.7]	73.9 [69.3-80.2]	172.4 [167.2-178.3]
2.50 mm‡	50	75	3	2	70	0	Simulated action potentials	Experimental optical action potentials	100 [100-100]	46.5 [29.1-62.3]	146.5 [128.9-161.5]
	max{50, 1/[1.95 min{DF_{AP},DF_{APS}}]}	75	3	2	70	0			99.9 [99.6-100]	79.8 [74.8-88.5]	179.0 [172.7-185.7]

Data are expressed as Median [P25th-P75th], N=117 optical mapping movies, N= 9 computer simulations of PersAF (see text for details)
iAM: instantaneous Amplitude Modulation, iFM: instantaneous Frequency Modulation
* Radius of a conventional ablation catheter
‡ Diameter of a conventional ablation catheter
µ Min iFM percentile without increasing iFM together with iAM ≥ iAM threshold to consider a quasi-stationary / meandering rotor footprint
AP: action potential
APS: absolute positive slope
DF: dominant frequency
RP: refractory period

Color legend: Median ≥ 90% ; 70% ≤ Median < 90% ; Median < 70%

Online Table VII. Optimal parameter combinations for single-signal detection of the rotational footprints produced by quasi-stationary rotors in computer simulations of PersAF in the presence of additive white gaussian noise

SNR (dB)	Tolerance	Signal Type	RP_{min} (ms)	iAM threshold (%)	Min. number of increasing iFM cycles	Min. number of increasing iAM cycles	Min. iFM percentile[µ]	Min. iAM excursion (%)	Sensitivity (%)	Specificity (%)	Sum (%)
30	1.25 mm*	Transmembrane	50	80	3	2	70	25	98.0 [94.9-99.3]	100 [99.4-100]	196.7 [192.7-198.6]
			$\max\{50, 1/[1.95 \min\{DF_{AP},DF_{APS}\}]\}$	75	3	2	70	25	83.0 [81.5-84.5]	100 [100-100]	183.0 [181.5-184.5]
		Unipolar pixel	50	85	3	2	70	25	92.1 [86.2-93.6]	97.2 [96.7-97.9]	189.6 [181.9-191.0]
			$\max\{50, 1/[1.95 \min\{DF_{UNI},DF_{ANS},DF_{BIP}\}]\}$	80	3	2	70	25	80.3 [77.1-83.8]	90.8 [90.3-93.3]	169.3 [167.3-174.5]
		Unipolar electrode	50	85	3	2	70	25	90.6 [86.2-92.2]	98.1 [97.9-98.6]	189.0 [183.4-190.4]
			$\max\{50, 1/[1.95 \min\{DF_{UNI},DF_{ANS},DF_{BIP}\}]\}$	75	3	3	70	0	80.7 [78.8-81.1]	91.2 [90.3-93.1]	171.2 [167.8-174.1]
	2.5 mm‡	Transmembrane	50	75	3	2	70	25	99.9 [98.7-100]	100 [99.5-100]	199.0 [197.6-199.8]
			$\max\{50, 1/[1.95 \min\{DF_{AP},DF_{APS}\}]\}$	75	3	2	70	25	94.0 [93.4-96.0]	100 [100-100]	194.0 [193.4-195.9]
		Unipolar pixel	50	85	3	2	70	25	97.6 [96.8-99.0]	97.1 [96.5-97.8]	195.2 [194.0-195.8]
			$\max\{50, 1/[1.95 \min\{DF_{UNI},DF_{ANS},DF_{BIP}\}]\}$	75	4	3	70	25	92.1 [84.6-94.9]	91.1 [89.4-93.4]	184.0 [176.7-185.0]
		Unipolar electrode	50	85	3	2	70	25	97.2 [96.3-98.3]	97.9 [97.8-98.6]	195.5 [194.4-196.7]
			$\max\{50, 1/[1.95 \min\{DF_{UNI},DF_{ANS},DF_{BIP}\}]\}$	75	3	3	70	25	94.4 [91.3-95.2]	90.7 [89.8-92.8]	183.7 [180.9-185.9]
20	1.25 mm*	Transmembrane	50	85	4	3	70	25	100 [100-100]	2.6 [0.7-37.4]	102.6 [100.7-137.1]
			$\max\{50, 1/[1.95 \min\{DF_{AP},DF_{APS}\}]\}$	75	4	3	70	25	99.2 [96.9-99.5]	95.0 [94.3-97.4]	193.4 [191.8-194.3]
		Unipolar pixel	50	85	4	2	70	25	94.4 [87.4-95.5]	96.8 [96.3-97.4]	190.4 [184.3-192.0]
			$\max\{50, 1/[1.95 \min\{DF_{UNI},DF_{ANS},DF_{BIP}\}]\}$	80	3	2	70	25	80.9 [79.0-84.3]	90.0 [89.4-91.7]	170.7 [168.8-173.7]
		Unipolar electrode	50	85	3	2	70	25	94.3 [88.6-95.2]	97.1 [96.2-97.5]	190.5 [184.8-192.8]
			$\max\{50, 1/[1.95 \min\{DF_{UNI},DF_{ANS},DF_{BIP}\}]\}$	75	3	3	70	25	81.6 [80.2-83.4]	91.3 [90.4-92.8]	172.8 [170.9-173.7]
	2.5 mm‡	Transmembrane	50	85	4	3	70	25	100 [100-100]	2.7 [0.7-37.8]	102.7 [100.7-137.8]
			$\max\{50, 1/[1.95 \min\{DF_{AP},DF_{APS}\}]\}$	75	4	3	70	25	100.0 [99.7-100]	96.6 [96.2-98.3]	196.5 [196.2-197.9]
		Unipolar pixel	50	85	4	3	70	25	95.6 [92.3-98.0]	98.4 [98.2-98.7]	194.0 [190.7-196.4]
			$\max\{50, 1/[1.95 \min\{DF_{UNI},DF_{ANS},DF_{BIP}\}]\}$	75	4	3	70	25	94.2 [84.6-94.9]	91.2 [89.6-93.3]	183.6 [176.6-185.3]
		Unipolar electrode	50	85	4	2	70	25	98.1 [95.0-98.9]	98.2 [97.9-98.6]	196.3 [193.8-197.4]
			$\max\{50, 1/[1.95 \min\{DF_{UNI},DF_{ANS},DF_{BIP}\}]\}$	80	4	2	70	25	91.3 [90.4-93.9]	92.6 [92.0-93.8]	184.6 [182.9-185.3]
10	1.25 mm*	Transmembrane	50	85	4	3	70	25	100 [100-100]	1.4 [0.5-10.4]	101.4 [100.5-110.4]
			$\max\{50, 1/[1.95 \min\{DF_{AP},DF_{APS}\}]\}$	80	4	3	70	25	99.6 [99.2-99.8]	95.6 [94.9-96.5]	195.3 [194.6-195.8]
		Unipolar pixel	50	85	4	3	70	25	100 [100-100]	1.2 [0.4-36.7]	101.2 [100.4-136.7]
			$\max\{50, 1/[1.95 \min\{DF_{UNI},DF_{ANS},DF_{BIP}\}]\}$	80	3	2	70	25	83.6 [82.5-88.9]	90.2 [89.65-92.6]	174.9 [172.5-178.4]
		Unipolar electrode	50	85	4	3	70	25	100 [100-100]	3.9 [3.0-39.2]	103.9 [103.0-139.2]
			$\max\{50, 1/[1.95 \min\{DF_{UNI},DF_{ANS},DF_{BIP}\}]\}$	75	3	3	70	25	83.6 [81.5-85.8]	90.8 [90.4-92.6]	173.4 [171.5-176.1]
	2.5 mm‡	Transmembrane	50	85	4	3	70	25	100 [100-100]	1.4 [0.5-10.5]	101.4 [100.5-110.5]
			$\max\{50, 1/[1.95 \min\{DF_{AP},DF_{APS}\}]\}$	85	4	3	70	25	100 [100-100]	96.7 [95.8-97.0]	196.7 [195.8-196.9]
		Unipolar pixel	50	85	4	3	70	25	100 [100-100]	1.2 [0.4-36.5]	101.2 [100.4-136.5]
			$\max\{50, 1/[1.95 \min\{DF_{UNI},DF_{ANS},DF_{BIP}\}]\}$	80	3	3	70	25	93.5 [92.3-96.5]	94.1 [93.8-95.3]	187.6 [186.3-189.3]
		Unipolar electrode	50	85	4	3	70	25	100 [100-100]	4.1 [3.2-39.1]	104.1 [103.2-139.1]
			$\max\{50, 1/[1.95 \min\{DF_{UNI},DF_{ANS},DF_{BIP}\}]\}$	80	4	2	70	25	91.7 [87.2-92.8]	95.3 [94.3-96.6]	185.9 [183.1-187.5]
0	1.25 mm*	Transmembrane	50	85	4	3	70	25	100 [100-100]	1.1 [0.3-9.8]	101.1 [100.3-109.8]
			$\max\{50, 1/[1.95 \min\{DF_{AP},DF_{APS}\}]\}$	85	4	3	70	25	94.8 [88.6-98.0]	84.9 [81.0-88.6]	178.1 [171.1-179.9]
		Unipolar pixel	50	85	4	3	70	25	100 [100-100]	1.4 [0.7-2.3]	101.4 [100.7-102.3]
			$\max\{50, 1/[1.95 \min\{DF_{UNI},DF_{ANS},DF_{BIP}\}]\}$	85	4	3	70	25	100 [99.8-100]	56.0 [53.3-59.8]	155.8 [153.3-159.7]
		Unipolar electrode	50	85	4	3	70	25	100 [100-100]	3.7 [3.0-27.1]	103.7 [103.0-127.1]
			$\max\{50, 1/[1.95 \min\{DF_{UNI},DF_{ANS},DF_{BIP}\}]\}$	85	4	3	70	25	100 [99.9-100]	57.7 [55.0-62.6]	157.7 [155.0-162.2]
	2.5 mm‡	Transmembrane	50	85	4	3	70	25	100 [100-100]	1.1 [0.3-9.9]	101.1 [100.3-109.9]
			$\max\{50, 1/[1.95 \min\{DF_{AP},DF_{APS}\}]\}$	85	4	3	70	25	100 [99.5-100]	84.7 [80.8-88.6]	184.7 [180.8-188.0]
		Unipolar pixel	50	85	4	3	70	25	100 [100-100]	1.4 [0.7-2.4]	101.4 [100.7-102.4]
			$\max\{50, 1/[1.95 \min\{DF_{UNI},DF_{ANS},DF_{BIP}\}]\}$	85	4	3	70	25	100 [100-100]	54.4 [52.3-59.2]	154.4 [152.3-159.2]
		Unipolar electrode	50	85	4	3	70	25	100 [100-100]	3.7 [3.2-41.1]	103.7 [103.2-141.1]
			$\max\{50, 1/[1.95 \min\{DF_{UNI},DF_{ANS},DF_{BIP}\}]\}$	85	4	3	70	25	100 [100-100]	56.3 [53.8-61.9]	156.3 [153.8-162.0]

Data are expressed as Median [P25th-P75th], N= 9 computer simulations of PersAF (see text for details)

iAM: instantaneous Amplitude Modulation, iFM: instantaneous Frequency Modulation

Color legend: Median ≥ 90% / 70% ≤ Median < 90% / Median < 70%

* Radius of a conventional ablation catheter

‡ Diameter of a conventional ablation catheter

µ Min iFM percentile without increasing iFM together with iAM ≥ iAM Threshold to consider a quasi-stationary / meandering rotor footprint

AP: action potential

APS: absolute positive slope

ANS: absolute negative slope

BIP: bipolar

DF: dominant frequency

RP: refractory period

UNI: unipolar

Unipolar electrode of 1 mm^2

SNR = 30 dB means that signal power = 1000 * noise power

SNR = 20 dB means that signal power = 100 * noise power

SNR = 10 dB means that signal power = 10 * noise power

SNR = 0 dB means that signal power = 1 * noise power

Online Table VIII. Accuracy in the measurement of iFM_{median} in computer simulations of PersAF in the presence of additive white gaussian noise

SNR (dB)	Signal Type	RP_{min} (ms)	iFM_{median} error (Hz) PS negative signals (n=136 549)	PS positive signals (n=13 451)	All signals (n=150 000)
∞	Transmembrane	50	0 [0, 0]	0.03 [0, 0.14]	**0 [0, 0]**
		max{50, 1/[1.95 min{DF_{AP},DF_{APS}}]}	0 [0, 0]	0 [0, 0]	**0 [0, 0]**
	Unipolar pixel	50	0 [0, 0]	0.02 [0, 0.09]	**0 [0, 0]**
		max{50, 1/[1.95 min{DF_{UNI},DF_{ANS},DF_{BIP}}]}	0 [0, 0]	0 [-0.01, 0.04]	**0 [0, 0]**
	Unipolar electrode	50	0 [-0.01, 0]	0.03 [0, 0.09]	**0 [0, 0]**
		max{50, 1/[1.95 min{DF_{UNI},DF_{ANS},DF_{BIP}}]}	0 [-0.01, 0]	0 [-0.02, 0.05]	**0 [-0.01, 0]**
30	Transmembrane	50	0 [0, 0.02]	0.05 [0, 0.23]	**0 [0, 0.03]**
		max{50, 1/[1.95 min{DF_{AP},DF_{APS}}]}	0 [0, 0.02]	0 [-0.04, 0.05]	**0 [0, 0.02]**
	Unipolar pixel	50	0 [-0.02, 0.02]	0.02 [-0.02, 0.12]	**0 [-0.02, 0.03]**
		max{50, 1/[1.95 min{DF_{UNI},DF_{ANS},DF_{BIP}}]}	0 [-0.02, 0.02]	0 [-0.04, 0.07]	**0 [-0.02, 0.03]**
	Unipolar electrode	50	0 [0, 0.02]	0.02 [-0.02, 0.12]	**0 [-0.01, 0.03]**
		max{50, 1/[1.95 min{DF_{UNI},DF_{ANS},DF_{BIP}}]}	0 [-0.01, 0.02]	0 [-0.04, 0.07]	**0 [-0.02, 0.03]**
20	Transmembrane	50	0.31 [0.07-3.3]	4.63 [3.54-5.72]	**0.41 [0.09-3.62]**
		max{50, 1/[1.95 min{DF_{AP},DF_{APS}}]}	0 [0, 0.04]	0.05 [0, 0.13]	**0 [0, 0.05]**
	Unipolar pixel	50	0 [0, 0.02]	0.05 [0, 0.13]	**0 [0, 0.03]**
		max{50, 1/[1.95 min{DF_{UNI},DF_{ANS},DF_{BIP}}]}	0 [-0.01, 0.02]	0 [-0.04, 0.07]	**0 [-0.02, 0.03]**
	Unipolar electrode	50	0 [0, 0.02]	0.05 [-0.02, 0.13]	**0 [-0.01, 0.03]**
		max{50, 1/[1.95 min{DF_{UNI},DF_{ANS},DF_{BIP}}]}	0 [-0.01, 0.02]	0 [-0.05, 0.07]	**0 [-0.02, 0.03]**
10	Transmembrane	50	7.02 [0.45, 7.75]	8.17 [6.70, 8.75]	**7.07 [0.96, 7.93]**
		max{50, 1/[1.95 min{DF_{AP},DF_{APS}}]}	0 [0, 0.08]	0.21 [0.10, 0.85]	**0.03 [0, 0.08]**
	Unipolar pixel	50	0.12 [0, 3.93]	4.33 [3.20, 5.04]	**0.17 [0, 4.1]**
		max{50, 1/[1.95 min{DF_{UNI},DF_{ANS},DF_{BIP}}]}	0 [0, 0.07]	0.04 [0, 0.12]	**0 [0, 0.08]**
	Unipolar electrode	50	0.09 [0, 3.79]	4.30 [3.17, 5.01]	**0.12 [0, 3.95]**
		max{50, 1/[1.95 min{DF_{UNI},DF_{ANS},DF_{BIP}}]}	0 [0, 0.07]	0.04 [-0.02, 0.13]	**0 [0, 0.08]**
0	Transmembrane	50	6.6 [4.9, 8.0]	8.3 [6.9, 8.9]	**6.8 [5.2, 8.2]**
		max{50, 1/[1.95 min{DF_{AP},DF_{APS}}]}	1.0 [0.5, 2.1]	1.9 [1.3, 3.1]	**1.14 [0.5, 2.1]**
	Unipolar pixel	50	6.95 [0.36, 7.71]	8.04 [6.6, 8.64]	**7.00 [0.99, 7.86]**
		max{50, 1/[1.95 min{DF_{UNI},DF_{ANS},DF_{BIP}}]}	0.07 [0, 0.21]	0.23 [0.12, 0.93]	**0.09 [0, 0.26]**
	Unipolar electrode	50	6.81 [0.36, 7.69]	8.05 [6.59, 8.63]	**6.91 [0.89, 7.86]**
		max{50, 1/[1.95 min{DF_{UNI},DF_{ANS},DF_{BIP}}]}	0.08 [0, 0.24]	0.23 [0.12, 0.87]	**0.09 [0, 0.26]**

Data are expressed as Median [P25th, P75th], N= 9 computer simulations of PersAF (see text for details)
iAM: instantaneous Amplitude Modulation, iFM: instantaneous Frequency Modulation
AP: action potential
APS: absolute positive slope
ANS: absolute negative slope
BIP: bipolar
DF: dominant frequency
RP: refractory period
UNI: unipolar
Unipolar electrode of 1 mm^2
SNR: Signal to Noise Ratio
SNR = ∞ means that there is not any noise
SNR = 30 dB means that signal power = 1000 * noise power
SNR = 20 dB means that signal power = 100 * noise power
SNR = 10 dB means that signal power = 10 * noise power
SNR = 0 dB means that signal power = 1 * noise power

Color legend:
- Low error
- Intermediate error
- Large error

Online Table IX. Characteristics at the time of pacemaker implantation and termination procedures in pigs

			**Pacemaker implantation**		**Termination procedure**	
**Group**	**Pig #**	**Sex**	Weight (kg)	Age (months)	Weight (kg)	Age (months)
Sham-operated	1	Female	33.0	4.5	85.0	14.0
	2	Male	44.5	6.7	100.0	15.8
	3	Male	45.0	6.0	94.0	15.8
Tool development	1	Male	38.0	4.8	127.0	18.6
	2	Female	36.5	4.5	92.0	16.4
	3	Female	45.0	6.1	87.0	18.5
	4	Female	49.0	5.9	96.0	14.8
Ablation	1	Male	46.0	6.1	81.5	11.9
	2	Male	38.5	4.5	103.0	23.2
	3	Male	41.0	3.9	70.0	9.7
	4	Male	44.0	5.8	110.0	21.5
	5	Male	41.0	6.6	68.0	13.8
	6	Male	43.0	4.4	101.0	14.9
	7	Male	45.5	5.1	102.5	14.9
	8	Male	49.0	7.9	140.0	23.1
	9	Male	40.0	5.2	80.0	12.0
	10	Male	41.5	5.2	86.0	13.0
	11	Male	44.5	5.1	96.5	14.5
	12	Female	54.0	6.8	114.0	16.4

All males were castrated

Online Table X. Baseline characteristics and mapping/ablation results in patients

	OBSERVATIONAL GROUP		INTERVENTIONAL GROUP		
	Patient #1	Patient #2	Patient #1	Patient #2	Patient #3
Age	69	69	36	54	50
Sex	Female	Male	Male	Male	Male
Weight (kg)	65	95	100	80	74
Height (m)	1.65	1.76	1.78	1.75	1.77
Body Mass Index	23.9	30.7	31.6	26.1	23.6
Comorbidities	Hypertension	CHD*<br>Hypertension<br>Diabetes	None	None	None
AF history (months)	41	156	76	21	27
PersAF history (months)	40	4	17	21	27
Duration of the latest PersAF episode (months)	4	3	12	5	9
Number of previous electrical cardioversions	2	0	1	3	4
Number of previous PVI / CTIA procedures	0	0	2 / 1	1 / 0	1 / 0
Number of failed AAD before the procedure	2	3	1	2	3
LA diameter (mm)	42	45	41	48	37
Mapping and ablation					
Number of reconnected pulmonary veins	N/A	N/A	0	0	1
Mapping time (min)	72	78	68	63	91
Driver regions	LSPV-LAA rigde,<br>LA roof	LSPV ostium, septum,<br>PLA, LA roof, LA floor,<br>LAA, RAA	RAA/RAFW,<br>LAA,<br>PLA, CS	postero-lateral LA	PLA, LAA,<br>region between<br>right PVs
Drivers/Atria surface ratio (%)	4.4	17.1	31.4	2.1	5.7
Max iFM $_{median}$ (Hz)	6.2	7.2	8.0	6.9	7.0
Leading driver ablation performed	No	No	Yes**	Yes	Yes
Acute AF termination during driver ablation	N/A	N/A	No	Yes	No
RF time until AF termination (min)	N/A	N/A	N/A	9.2	N/A
Total RF time for driver ablation (min)	N/A	N/A	11.5	10.2	23.1
Ablation aborted before reaching endpoints	N/A	N/A	Yes**	No	No
Atrial DF on ECG decreased >10% after ablation	N/A	N/A	No	N/A	Yes
Complications	No	No	No	No	No
Follow-up (16 months)					
AAD during the first 6 months	Flecainide	Propafenone	Flecainide	Amiodarone	Flecainide
AAD after 6 months of follow-up	None	Propafenone	N/A***	None	None
Complications	No	No	No	No	No
AF recurrence	No	Yes	Yes	No	No

AAD: antiarrhythmic drugs; AF: atrial fibrillation; CS: coronary sinus; CHD: Coronary heart disease; CTIA: cavotricuspid isthmus ablation; N/A: not applicable; LA: left atrium; LAA: LA appendage, LSPV: left superior pulmonary vein; PLA: posterior LA; PersAF: persistent atrial fibrillation; PV: pulmonary vein; PVI: pulmonary vein isolation; RA: right atrium; RAA: RA appendage; RAFW: RA free wall; RF: radiofrequency.

* Stenting of 1 coronary vessel because of chronic stable angina. Normal left ventricular ejection fraction.

** Regions of higher than surrounding iFM_{median} were considered too large to be safely targeted with catheter ablation.

*** Rate control strategy

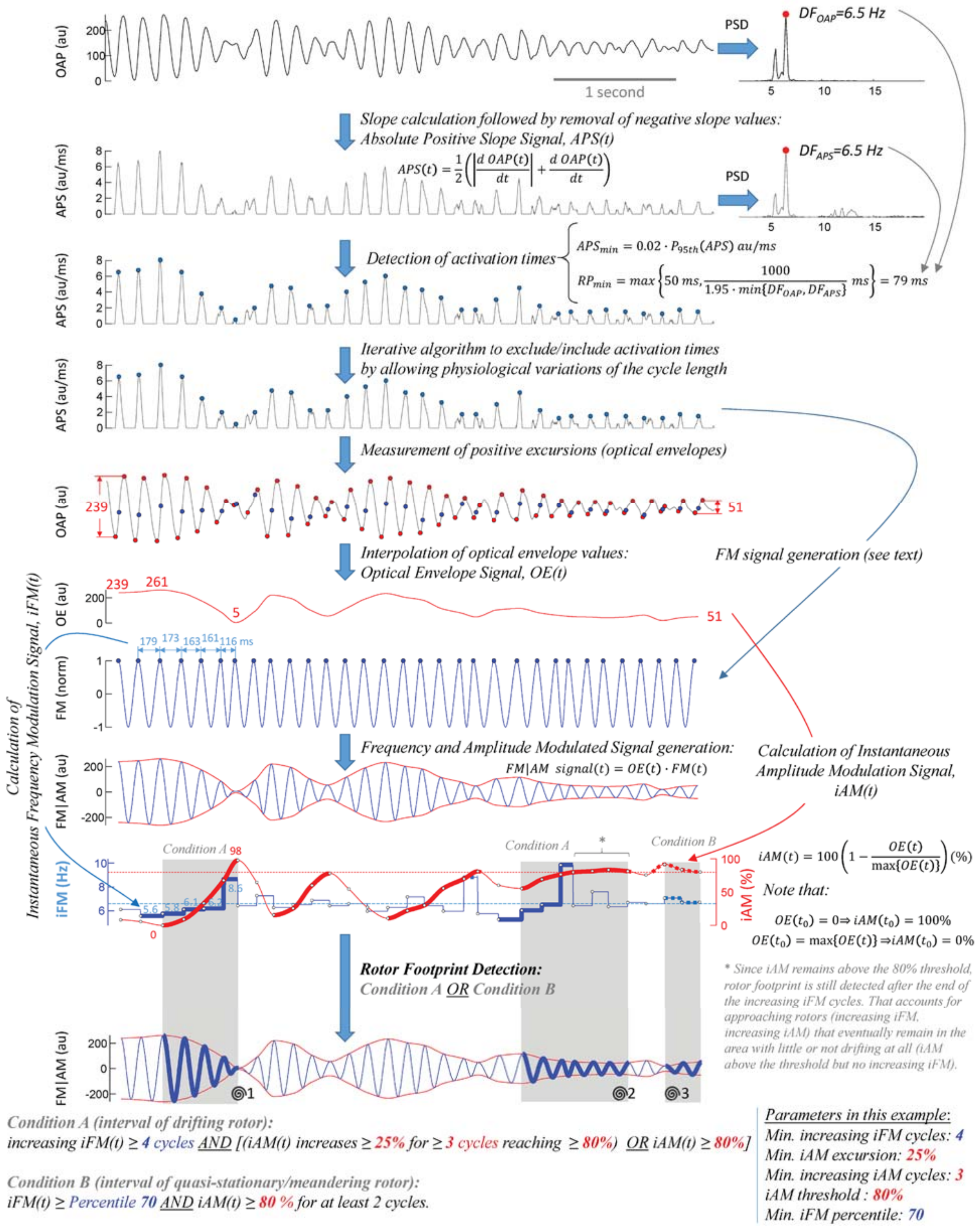

Online Figure I. Single-signal algorithm to detect rotational footprints based on the instantaneous amplitude and frequency modulations (iAM / iFM) contained in optical signals during AF. In this version of the algorithm, the minimum refractory period used to calculate activations in the time-domain is calculated from the dominant frequencies of the OAP and APS signals. The rest of the figure is self-explanatory. More details are provided in the text. **APS**: absolute positive slope signal, **FM|AM**: Frequency and amplitude modulated signal, **FM**: frequency modulated signal, **iAM:** Instantaneous amplitude modulation signal, i**FM**: Instantaneous frequency modulation signal, **OAP**: optical action potentials, **OE**: optical envelope signal, **PSD:** power spectral density.

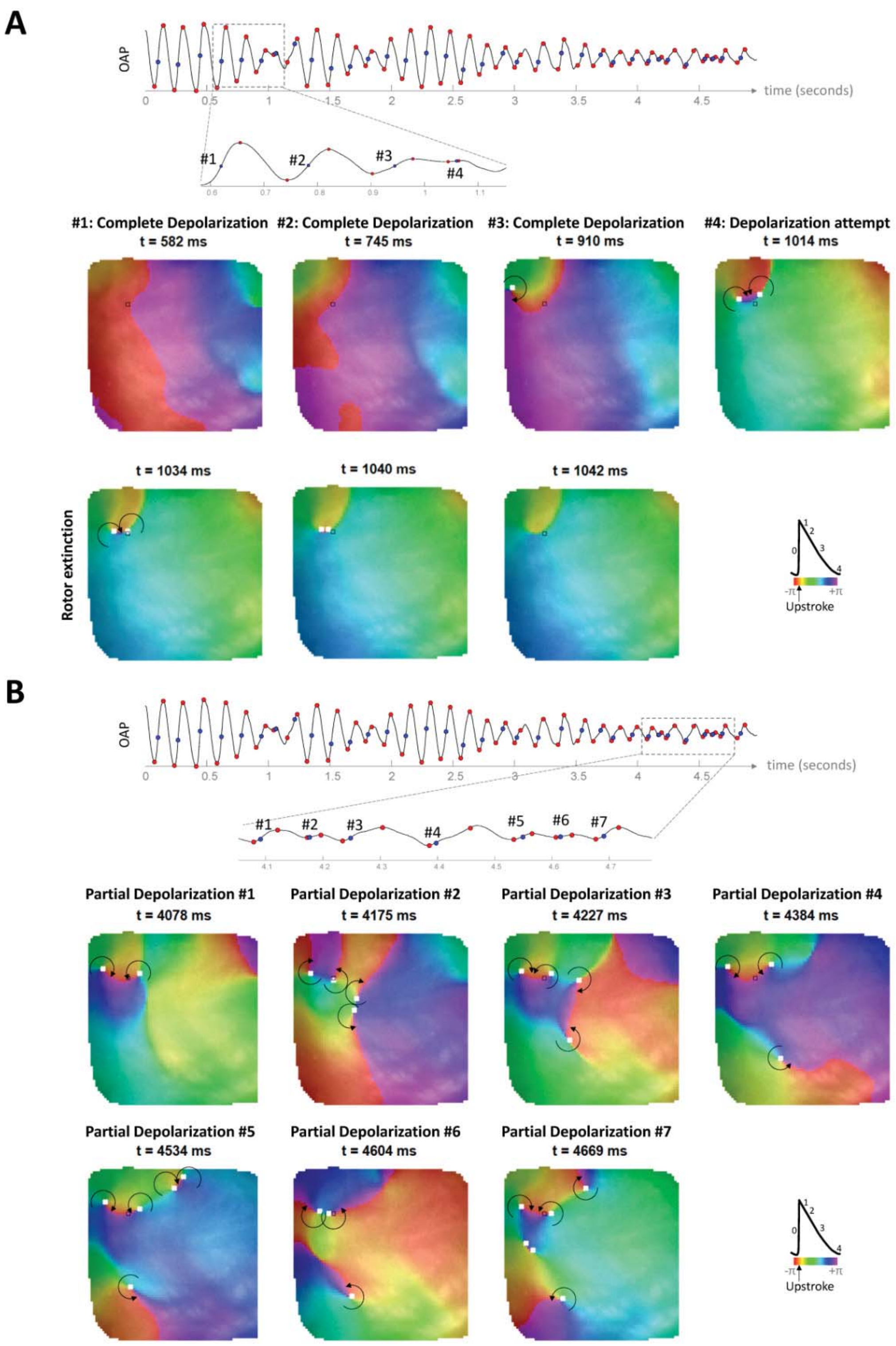

Online Figure II. Multiple depolarization attempts/partial depolarizations are present in an optical signal during the intervals in which phase singularities (white circles) meander through the pixel surroundings. They are the result of the rotational wavefront crossing the pixel in a seemingly very high, non-physiological rate. Note that although the pixel is framed with a black line, it could be necessary to zoom in the figure to visualize it due to its tiny size. **A.** Three complete activations followed by a depolarization attempt in a short period of time before the figure-of-eight reentry terminated. **B.** Seven depolarization attempts / partial depolarizations in less than 600 ms, which could be considered as a non-physiological activation rate. The phase movie during this time interval is shown in **Online Movie II**.

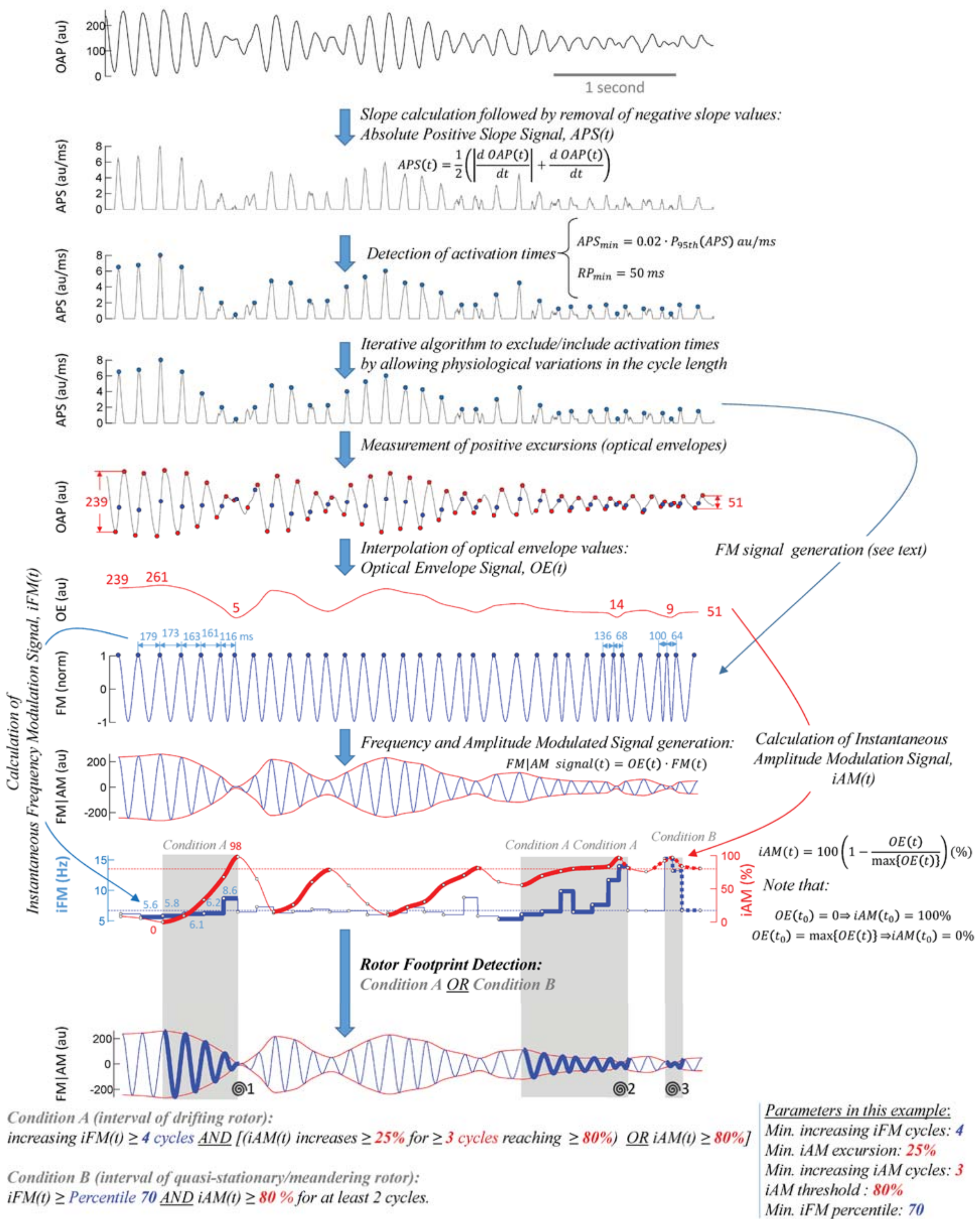

Online Figure III. Single-signal algorithm to detect rotational footprints based on the instantaneous amplitude and frequency modulations (iAM/iFM) contained in optical signals during AF. In this version of the algorithm, the minimum refractory period is fixed to 50 ms regardless of the dominant frequencies of the OAP and APS signals. Thus, the algorithm is capable to account for high-rate seemingly non-physiological partial depolarizations like the ones shown in **Online Figure**/**Movie II**. This makes the detection of activations very sensitive, but potentially unspecific. The rest of the figure is self-explanatory. More details are provided in the text. Acronyms as in **Online Figure I**.

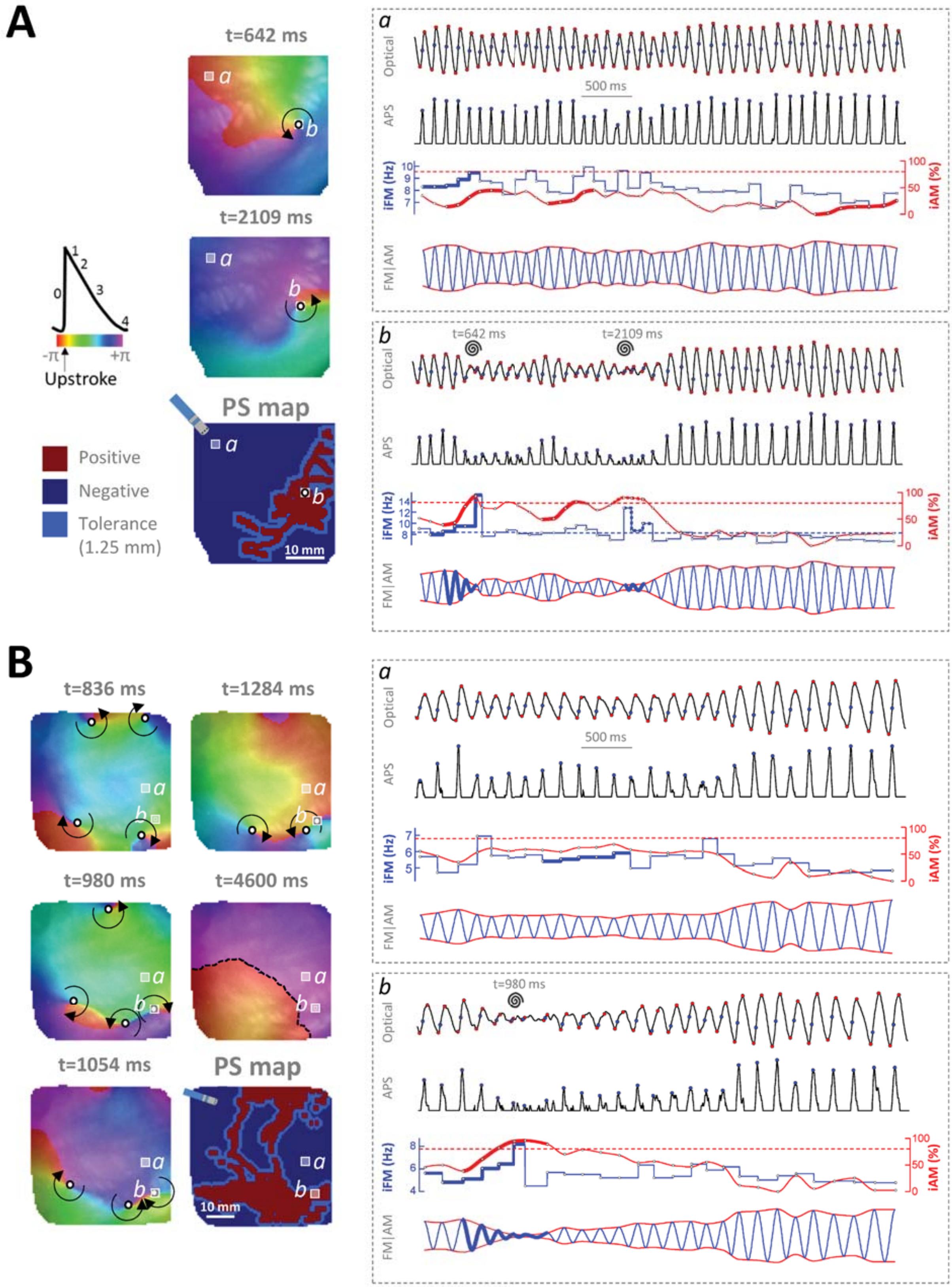

Online Figure IV. Additional examples of the processing applied to single optical signals to detect the footprint of rotational activity. A. Phase movie snapshots, phase singularity map and sample signals of a phase movie from sheep #1 (**Online Movie V**). **B.** Phase movie snapshots, phase singularity map and sample signals of a phase movie from sheep #2 (**Online Movie VI**). Sample pixels are displayed as grey squares (enlarged to ease visualization). Symbols, red points, blue points, lines and acronyms as in **Figure 2** of the main manuscript.

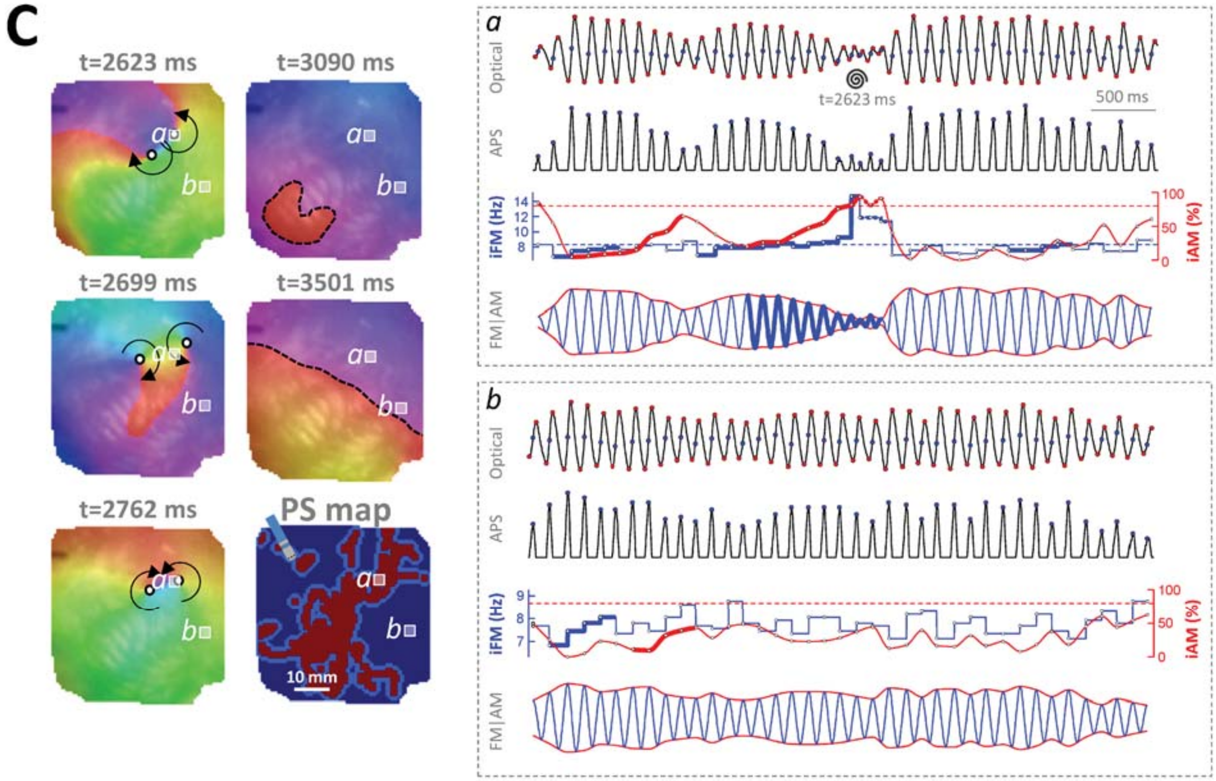

Online Figure IV (continued). Additional examples of the processing applied to single optical signals to detect the footprint of rotational activity. C. Phase movie snapshots, phase singularity map and sample signals of a phase movie from sheep #4 (**Online Movie VIII**). Sample pixels are displayed as grey squares (enlarged to ease visualization). Symbols, red points, blue points, lines and acronyms as in **Figure 2** of the main manuscript.

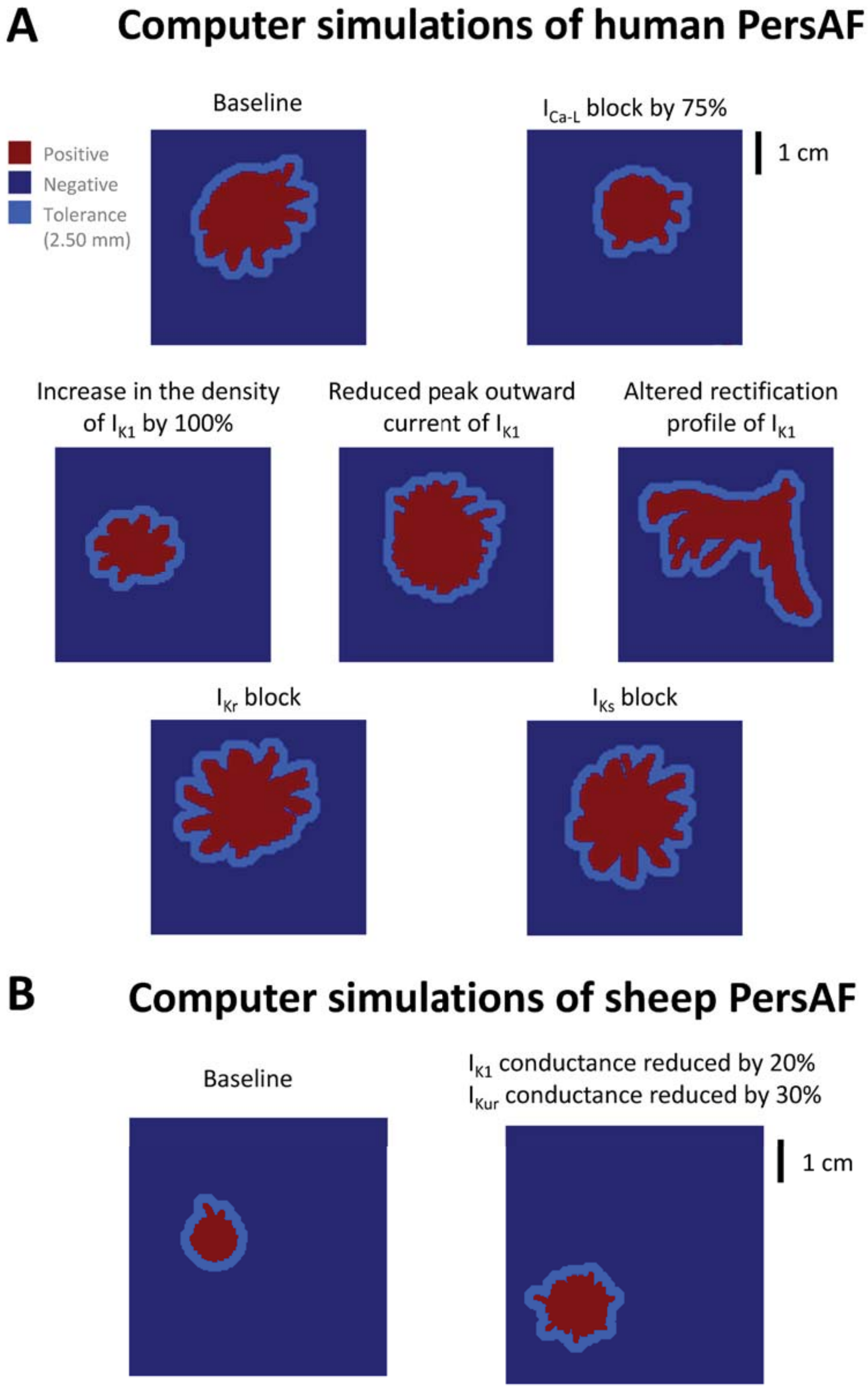

Online Figure V. Phase singularity maps and ionic features of computer simulations of rotors using ionic models of PersAF. Notably, all but one displayed a quasi-stationary, non-drifting behavior. The latter was likely due to the homogeneous and isotropic nature of the simulated 2D sheet. These simulations were used to perform the analyses presented in **Online Tables IV-VIII** and **Online Figures XVIII-XIX**. **A.** 2D simulations using a human model of atrial cells during PersAF. More specific details can be found elsewhere.[21] **B.** 2D simulations using a sheep model of atrial cells during PersAF. More specific details can be found elsewhere.[22]

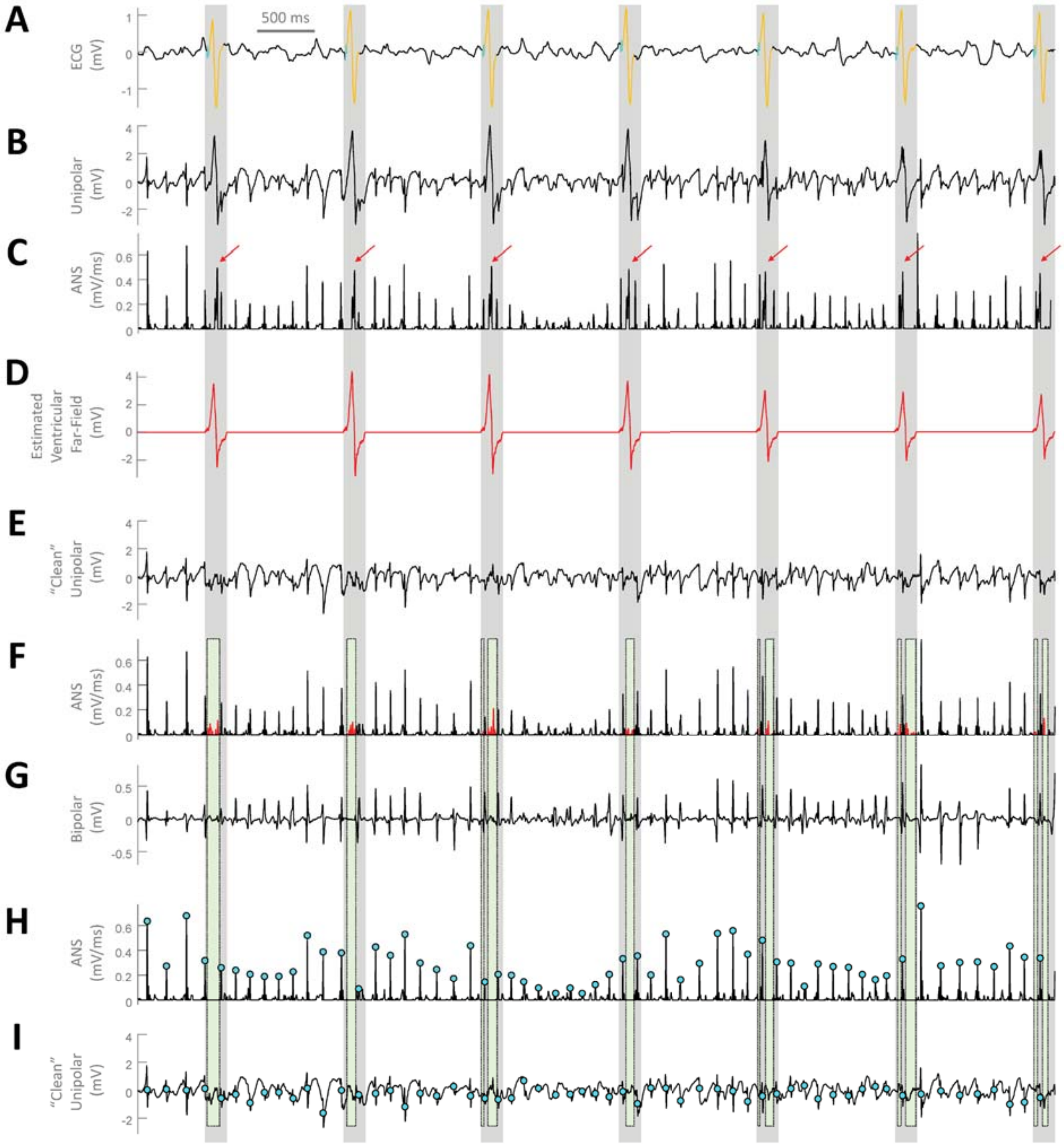

Online Figure VI. Ventricular far-field minimization and rejection algorithm. **A.** Surface ECG signal. QRS complexes are highlighted in orange and stimulation artifacts in cyan. The grey intervals are the ones in which the minimization and rejection algorithm will be applied. **B.** Sample raw unipolar signal from the coronary sinus with overt ventricular far-field. **C.** Absolute Negative Slope (ANS) signal that will be later used to detect activation times. The red arrows indicate the spurious peaks generated by the negative deflections of the ventricular far-field. Some of them may actually be simultaneous with true atrial activations. **D.** A ventricular far-field signal is estimated using Principal Component Analysis (PCA). See details in the text. **E.** Unipolar signal after subtraction of the estimated ventricular far-field in D. **F.** The ANS is calculated again from the unipolar signal shown in E after rejection/minimization of ventricular far-field. Note that some residual negative slope ventricular activity is still present (in red). **G.** Then, the corresponding bipolar signal is analyzed during the gray intervals and the negative slope activity in F between atrial activations in the bipolar signal (time intervals with green background) is removed, because very likely corresponds to residual ventricular activity. **H.** Negative slope activity during green intervals is removed from ANS prior to detecting activations as the times with the maximum negative slopes (see **Online Figure IX** for more details about detecting activations). **I.** 'Clean' unipolar signal with cyan dots marking the activation times detected in the ANS shown in H.

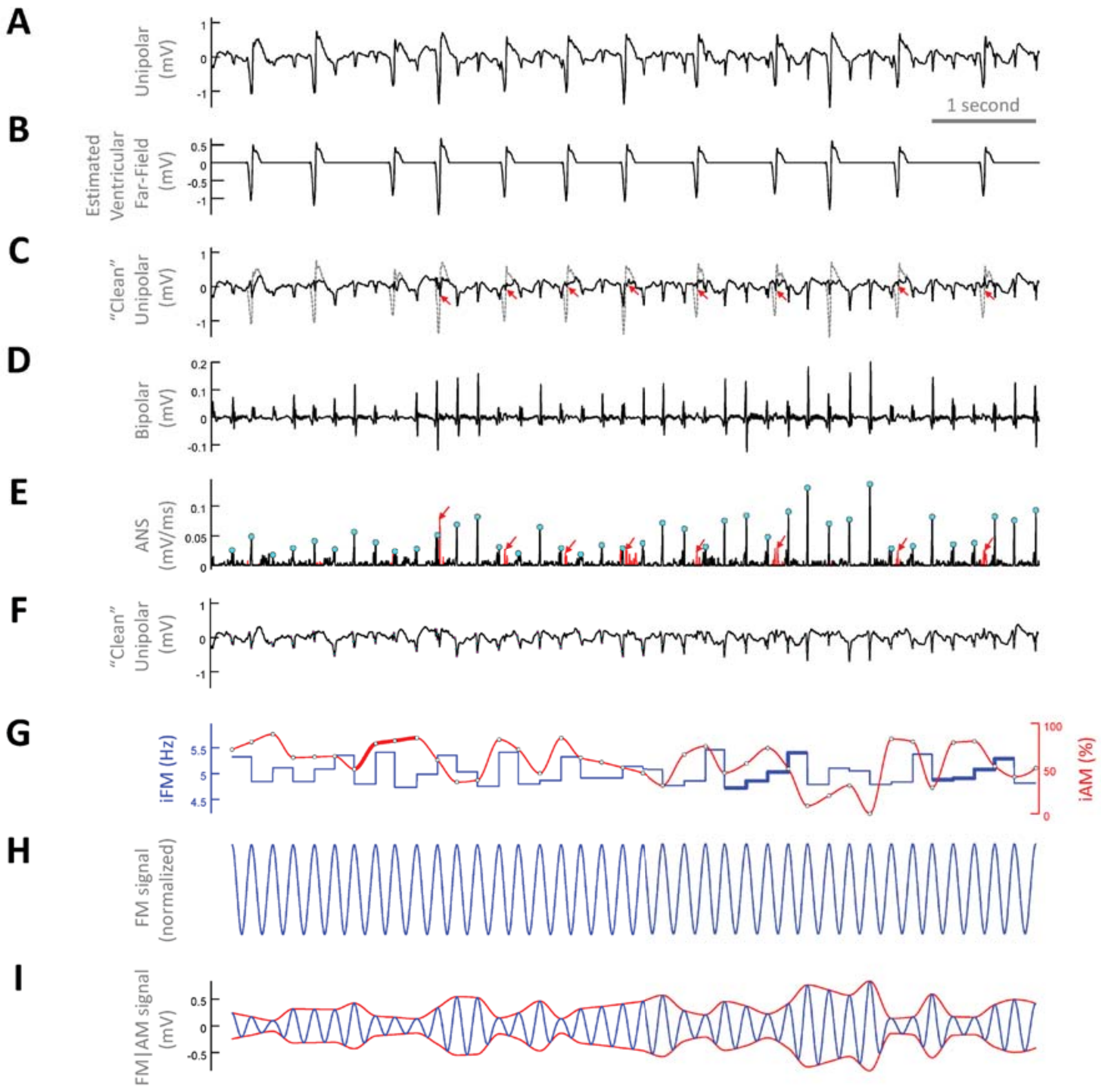

Online Figure VII. Example of ventricular far-field minimization and rejection algorithm performance in a PersAF patient with ventricular activity of variable cycle length. **A.** Sample raw unipolar signal with overt ventricular far-field. **B.** A ventricular far-field signal is estimated using Principal Component Analysis (PCA). **C.** Unipolar signal after subtraction of the estimated ventricular far-field in B. The original unipolar signal is displayed with a dashed gray trace. Note that some residual ventricular activity is still present (red arrows). **D.** Bipolar signal. **E.** Absolute Negative Slope (ANS) signal calculated from the unipolar signal shown in C after rejection/minimization of ventricular far-field. This signal is used to detect activation times. The red intervals and arrows indicate the spurious peaks generated by the residual negative deflections of the ventricular far-field that remained after the first PCA algorithm. Those intervals are blanked thanks to the second algorithm. Specifically, the corresponding bipolar signal in D is analyzed and the activity in E between atrial activations in the bipolar signal is removed (red intervals), because very likely corresponds to residual ventricular activity. Therefore, no atrial activations are detected in such red intervals. **F.** 'Clean' unipolar signal with cyan dots marking the activation times detected in the ANS shown in E. Magenta dots mark the start and the end of negative deflections that correspond to activations (zooming in is needed to visualize cyan and magenta points). **G.** iFM and iAM signals calculated from activation times and amplitudes of the corresponding negative deflections, respectively. **H.** FM signal obtained by adjusting a sinusoidal wave to reach its maxima at the activation times. **I.** FM|AM signal that incorporates the amplitude modulation present in the negative deflections of the unipolar signal into the FM signal in H.

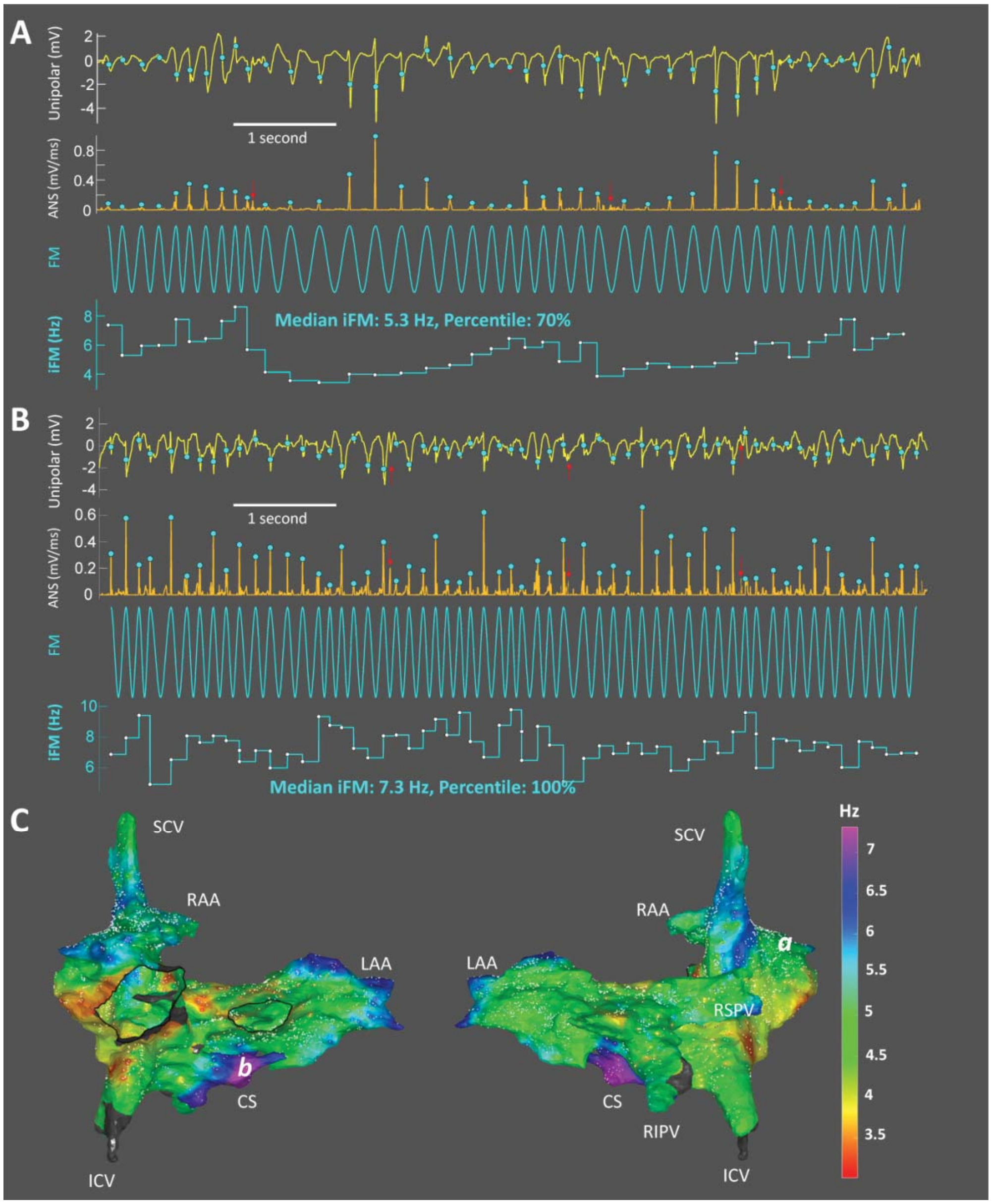

Online Figure VIII. Generation of median instantaneous frequency modulation (iFM) maps from *in vivo* electroanatomical data in a sample pig case with persistent AF (PersAF). This process enabled to detect high-hierarchy leading-driver regions suitable for ablation. **A.** Top row: unipolar signal from the RA (‘*a*’ in panel C) after ventricular far-field minimization. Red arrows mark the residual negative slope activity (see **Online Figures VI-VII** for more details about the ventricular far-field minimization/rejection algorithm). Local activation times are detected on the Absolute Negative Slope (ANS) Signal (orange tracing) using the algorithm described in **Online Figure IX**. This activation times and their separations are used to create a sinusoidal frequency modulated signal (FM, arbitrary units) and the iFM signal (measured in Hz). The median value of the latter signal is used as a measure of the hierarchy level of such specific spatial location during fibrillation (5.3 Hz, 70th percentile). **B.** Top row: unipolar signal from the CS (‘b’ point in panel C) after ventricular far-field minimization. Red arrows mark the residual negative slope activity. Again, the median value of the iFM signal is used as a measure of the hierarchy level of that specific spatial location (7.3 Hz, maximum value) during fibrillation. **C.** Leading-driver map obtained by interpolating the median iFM values at the 3204 points used to generate it (small white dots). The ‘islands’ of higher than surrounding iFM median values are considered leading-drivers. Left: anterior view. Right: posterior view. CS: coronary sinus, ICV: inferior cava vein, LAA: left atrial appendage, RAA: right atrial appendage, RSPV: right superior pulmonary vein, SCV: superior cava vein. **Online Figures XXVII-XXVIII** show the similarities and differences between the presented iFM approach and previous attempts to quantify hierarchy during atrial fibrillation by means of Dominant Frequency.[37, 41]

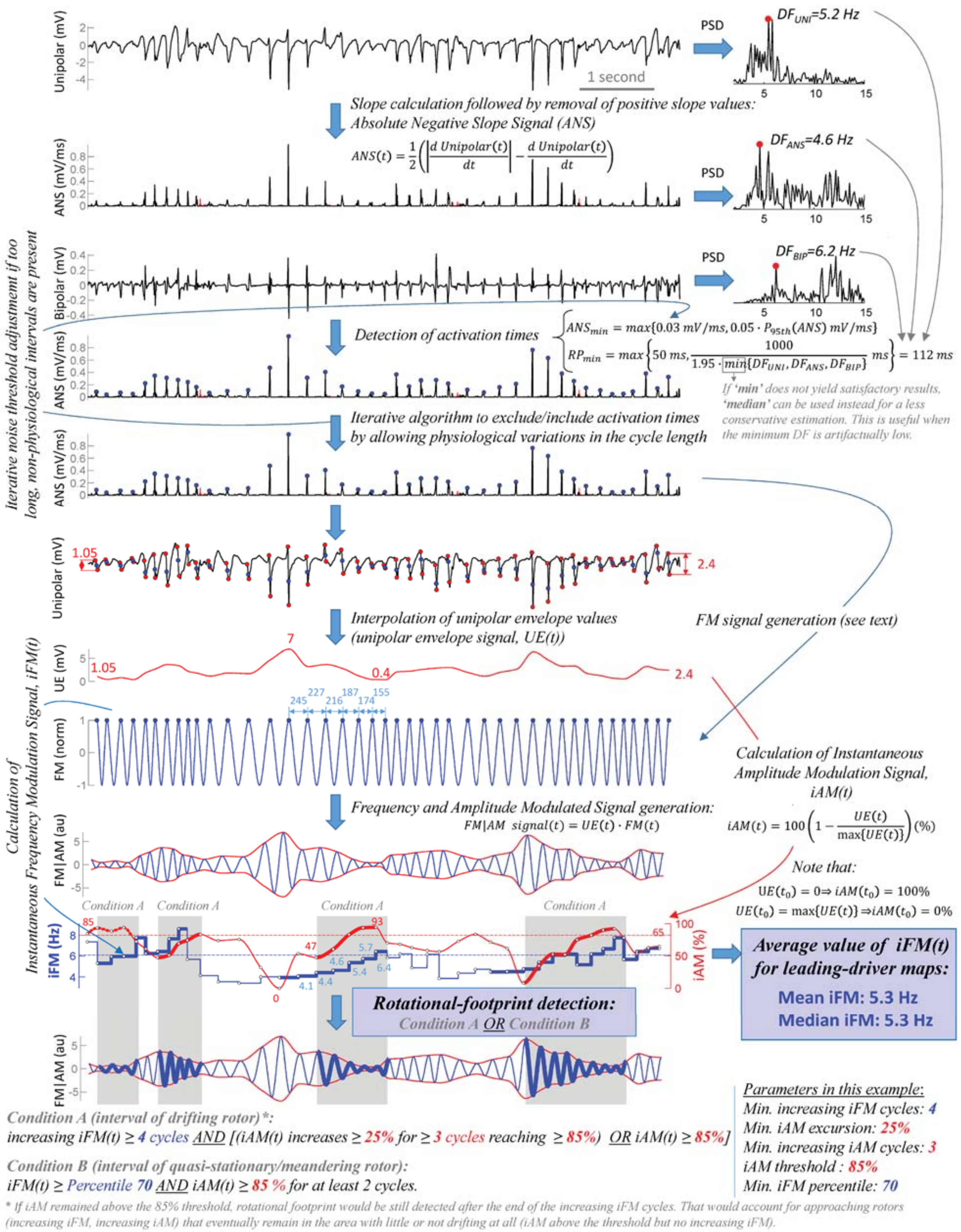

Online Figure IX. Single-signal algorithm to detect rotational-footprints based on the instantaneous amplitude and frequency modulations (iAM / iFM) contained in unipolar electrical signals during *in vivo* persistent AF. More details are provided in the text. **ANS**: absolute negative slope, **FM|AM**: Frequency and amplitude modulated signal, **FM**: frequency modulated signal, **iAM:** Instantaneous amplitude modulation signal, i**FM**: Instantaneous frequency modulation signal, **PSD:** power spectral density, **UE**: unipolar envelope signal.

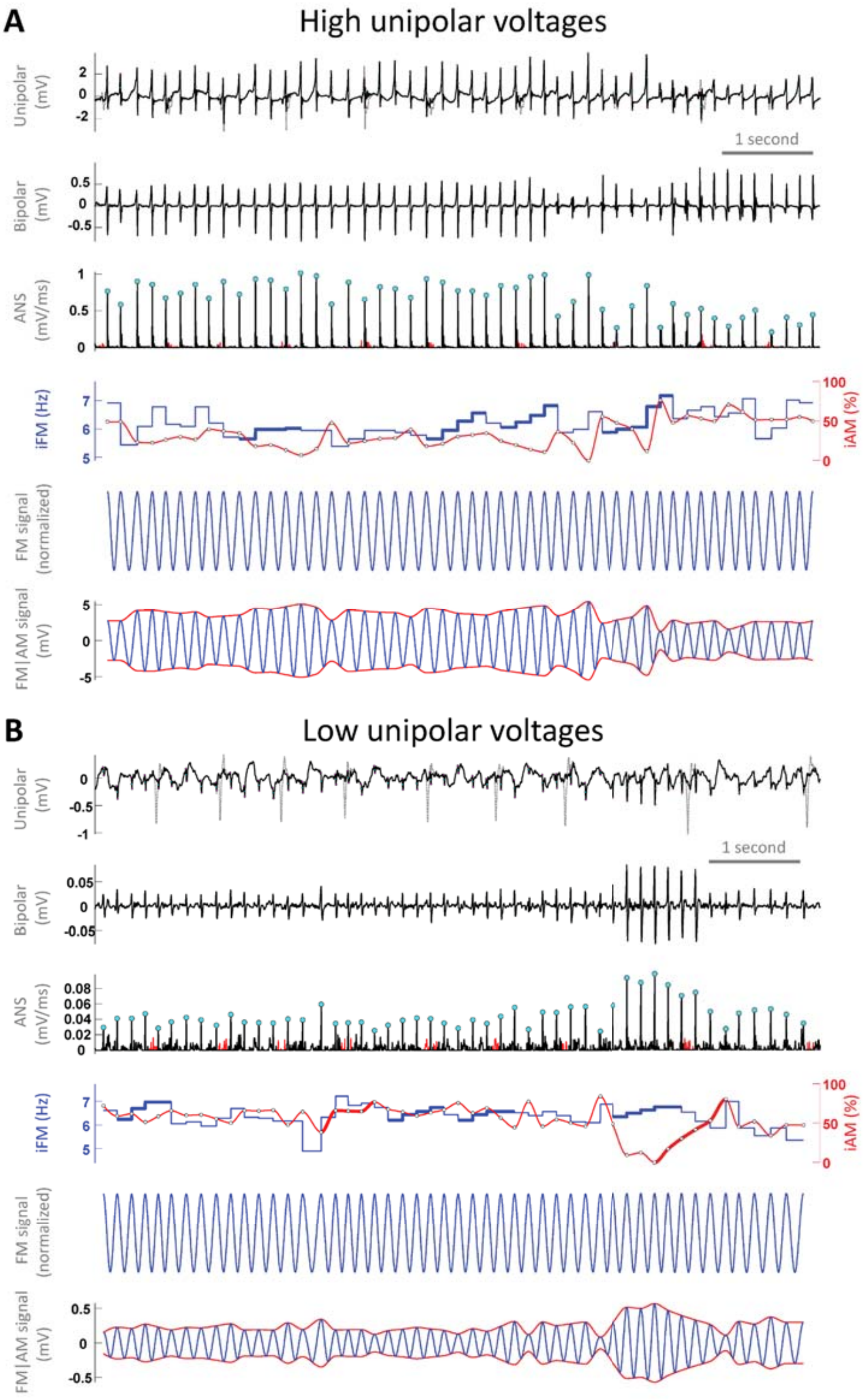

Online Figure X. iAM tracks the percent changes in unipolar negative deflection amplitudes regardless of the fact that such amplitudes are high **(A)** or low **(B)**. Note the completely different orders of magnitude in the vertical axes of unipolar, bipolar and ANS signals in A and B, obtained from one of our patients. Nevertheless, iFM, iAM, FM and FM|AM signals can be accurately calculated in both cases. Activation times and the start/end of their corresponding negative deflections are marked in the unipolar signals with cyan and magenta points, respectively. These points are only visible after zooming in.

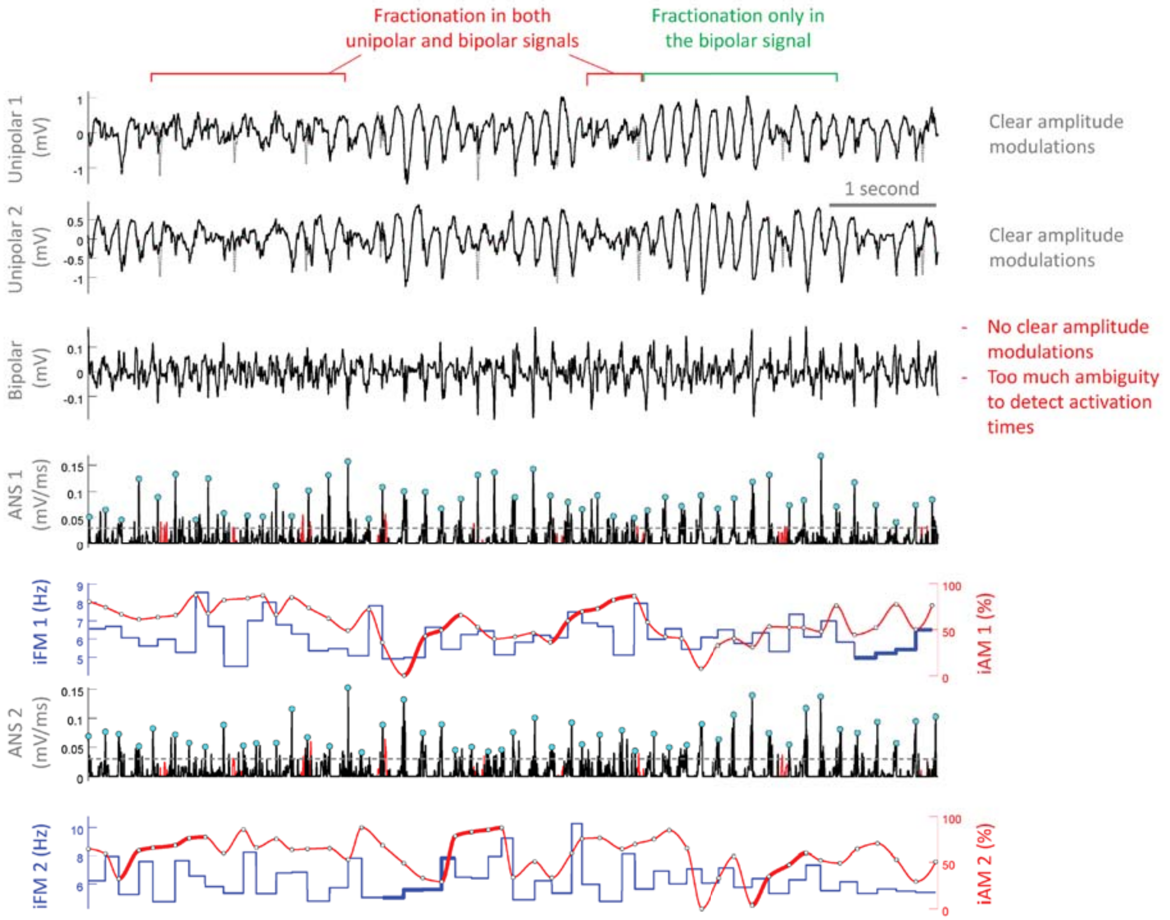

Online Figure XI. Bipolar configurations can result in increased fractionation. Our algorithm mainly relies in unipolar signals acquired with tiny electrodes, which reduces the degree of signal fractionation. Also, amplitude modulations observed in unipolar signals are often not detectable in bipolar signals. The signals shown here were obtained from one of our patients. Note that despite some fractionation is present in the unipolar signals, activations can be detected with not too much ambiguity thanks to the associated ANS signals derived from the unipolar signals. From such activations and the amplitude of their negative deflections, the iFM and iAM signals can be computed. Importantly, the iAM signal cannot be computed from the bipolar signal since amplitude modulations are not discernible in it. In addition, the bipolar amplitudes depend on wavefront orientation and are not directly proportional to those in the underlying action potentials. Furthermore, in cases like this one, the calculation of iFM from the bipolar signal would be much more challenging. Activation times and the start/end of their corresponding negative deflections are marked in the unipolar signals with cyan and magenta points, respectively. These points are only visible after zooming in.

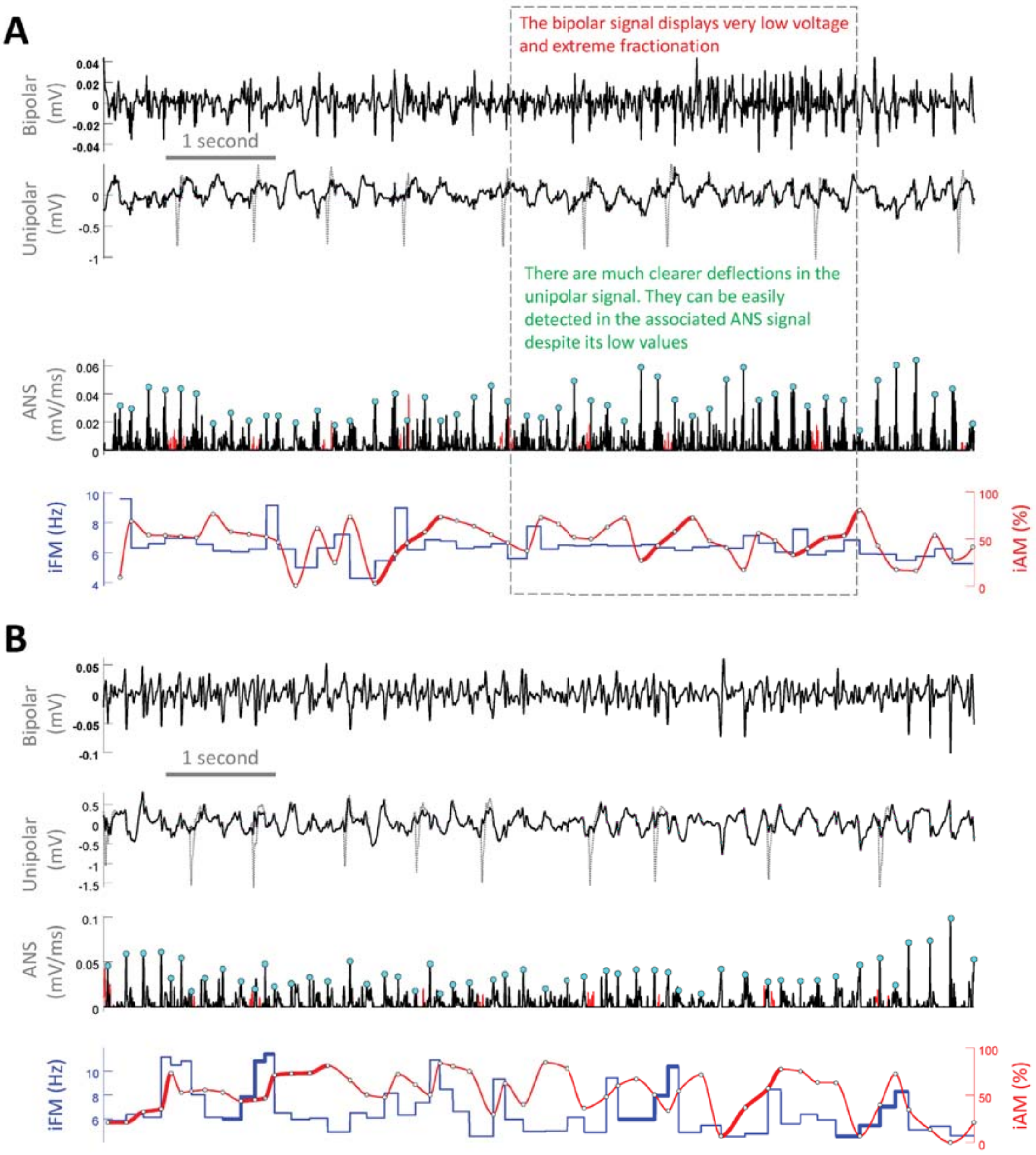

Online Figure XII. Examples of the algorithm performance with highly fractionated signals. These signals were obtained from a patient. Note that despite fractionation (that is substantial in the bipolar signals), the detection of activation times and the calculation of iFM using the ANS signal are accurate. **A.** Sample signal with high fractionation during the interval framed with a dashed line. **B.** Sample signal with high fractionation during the whole 8 seconds of acquisition. Activation times and the start/end of their corresponding negative deflections are marked in the unipolar signals with cyan and magenta points, respectively. These points are only visible after zooming in.

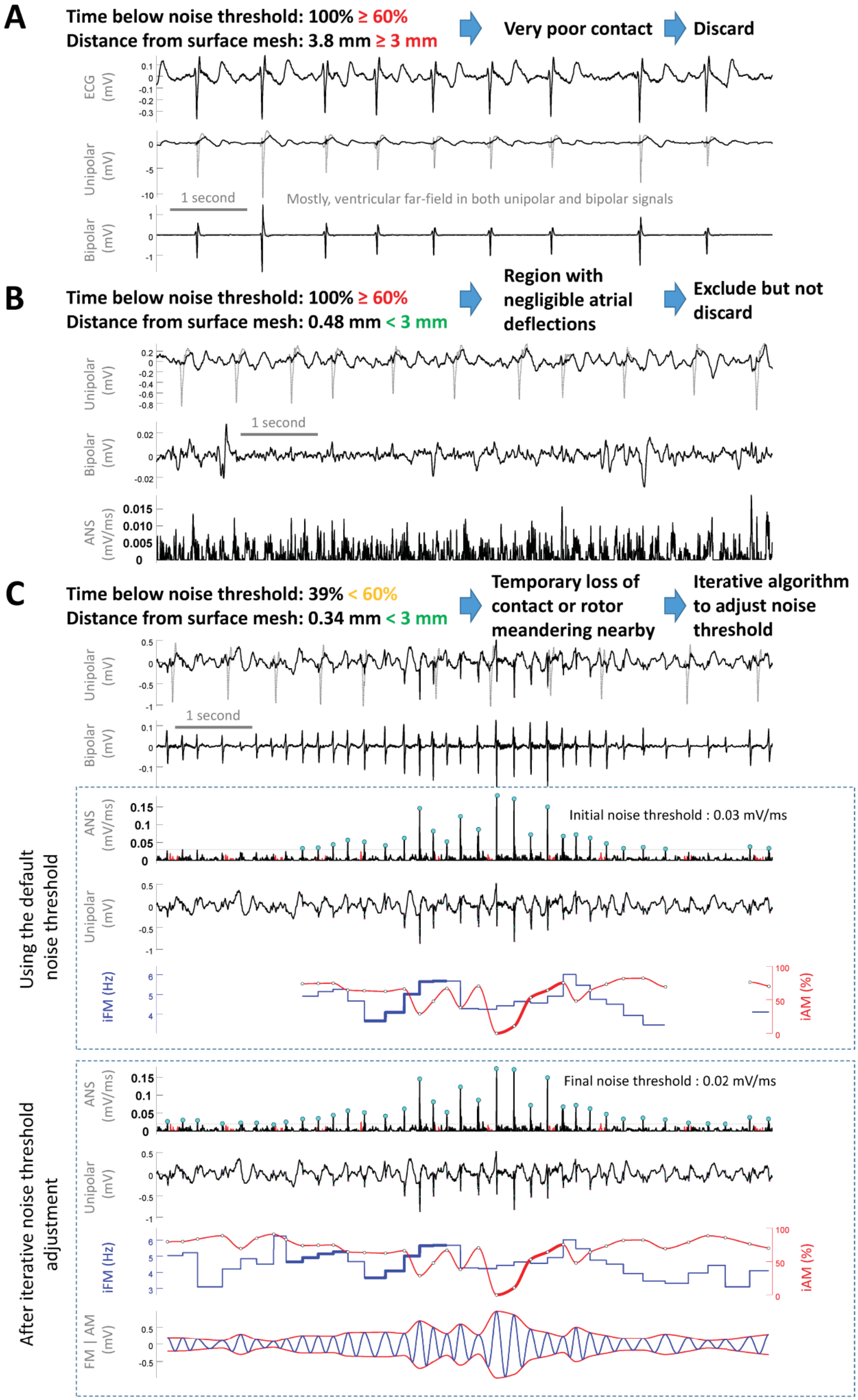
A
Time below noise threshold: 100% ≥ 60%
Distance from surface mesh: 3.8 mm ≥ 3 mm
Very poor contact
Discard
ECG (mV)
Unipolar (mV)
Bipolar (mV)
1 second
Mostly, ventricular far-field in both unipolar and bipolar signals
B
Time below noise threshold: 100% ≥ 60%
Distance from surface mesh: 0.48 mm < 3 mm
Region with negligible atrial deflections
Exclude but not discard
Unipolar (mV)
Bipolar (mV)
ANS (mV/ms)
1 second
C
Time below noise threshold: 39% < 60%
Distance from surface mesh: 0.34 mm < 3 mm
Temporary loss of contact or rotor meandering nearby
Iterative algorithm to adjust noise threshold
Unipolar (mV)
Bipolar (mV)
1 second
Using the default noise threshold
ANS (mV/ms)
Initial noise threshold : 0.03 mV/ms
Unipolar (mV)
iFM (Hz)
iAM (%)
After iterative noise threshold adjustment
ANS (mV/ms)
Final noise threshold : 0.02 mV/ms
Unipolar (mV)
iFM (Hz)
iAM (%)
FM | AM (mV)

Online Figure XIII. Examples of signals discarded (A), excluded from (B) or included in (C) the analysis depending on i) the percentage of time in which the ANS signal is below the noise threshold, and ii) the distance between the location where the unipolar signal was acquired and the surface mesh generated by the electroanatomical mapping system. **A.** Sample signal that mostly consists of ventricular far-field (even in the bipolar configuration) due to scarce or absent electrode-tissue contact. It was acquired farther than 3 mm from the surface mesh and atrial ANS values were below the noise threshold during 100% of the acquisition time. Therefore, the signal was discarded. **B.** Sample signal that was acquired with good contact from a distal portion of a vein. Although it was acquired close to the mesh surface, in that location there was not local atrial near-field. Therefore, the ANS values were below the noise threshold during 100% of the acquisition time. Signals with reasonable contact but a high percentage of time below the noise threshold are not discarded, but the parameters measured from them are not included in the maps. However, they can be selected in the map for visual inspection. Then, they can be tagged, or even included in the maps at the operator discretion. **C.** Sample signal that displayed ANS values below the noise threshold for 39% of the acquired time, even though it was acquired with good contact. In cases like this one, an iterative noise threshold adjustment was performed in order to find physiological deflections within intervals of subthreshold ANS values. Thus, the algorithm lowered iteratively the noise threshold in steps of 0.005 mV until the physiological activations were detected. In the absence of physiological activations, the intervals in which ANS values were below the noise threshold remain blanked so that such intervals did not erroneously lower the calculated iFM.

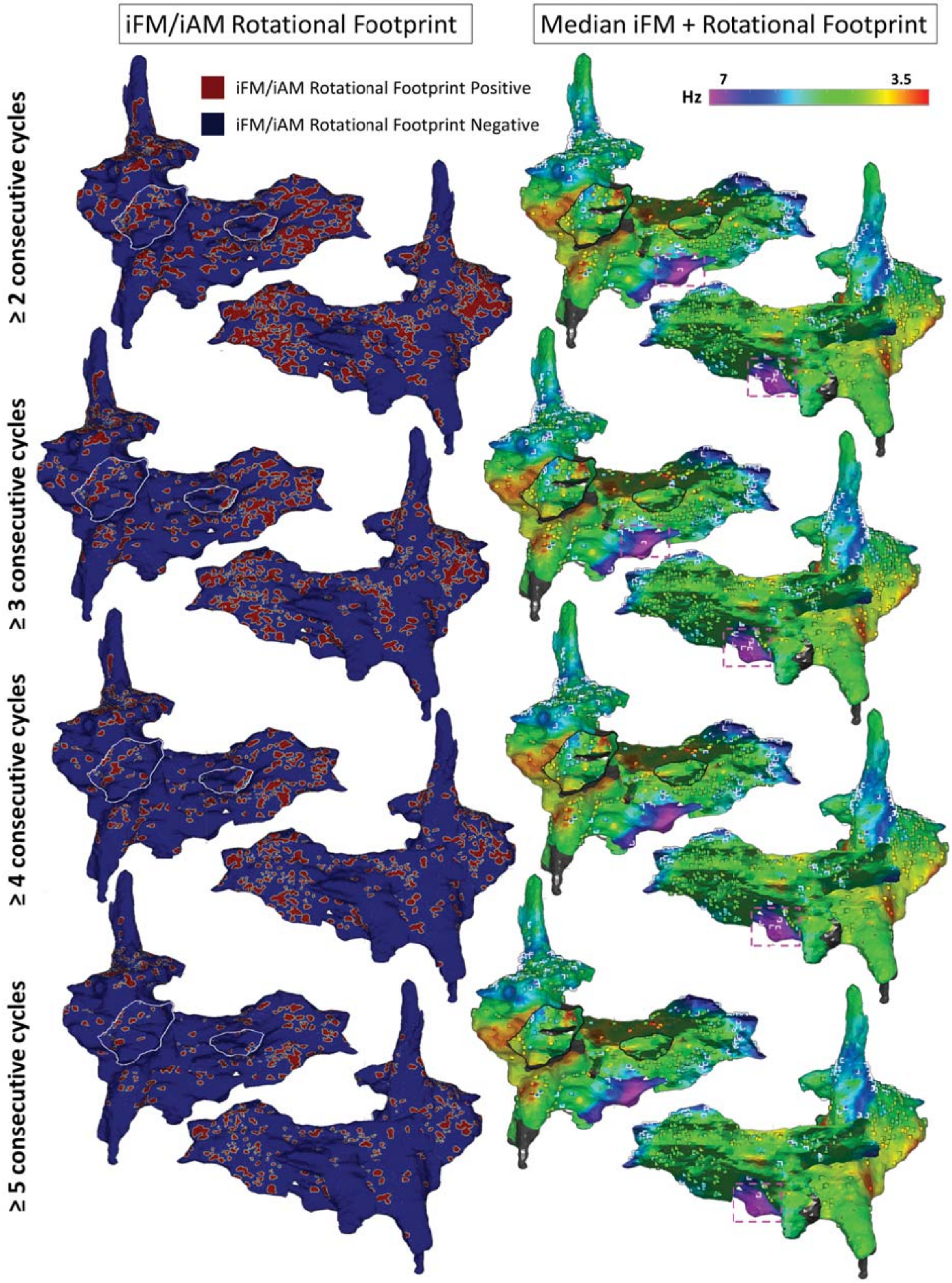

Online Figure XIV. This figure shows how the rotational-footprint and the combined maps (median iFM + rotational-footprint) change, depending on the number of positive consecutive cycles required to consider a positive rotational-footprint detection (from top to bottom, a minimum of 2, 3, 4 and 5 consecutive cycles with positive detection). The higher the number of consecutive cycles required for detecting a rotational-footprint, the patchier the red areas are. However, the anatomical regions with positive rotational footprints are roughly the same.

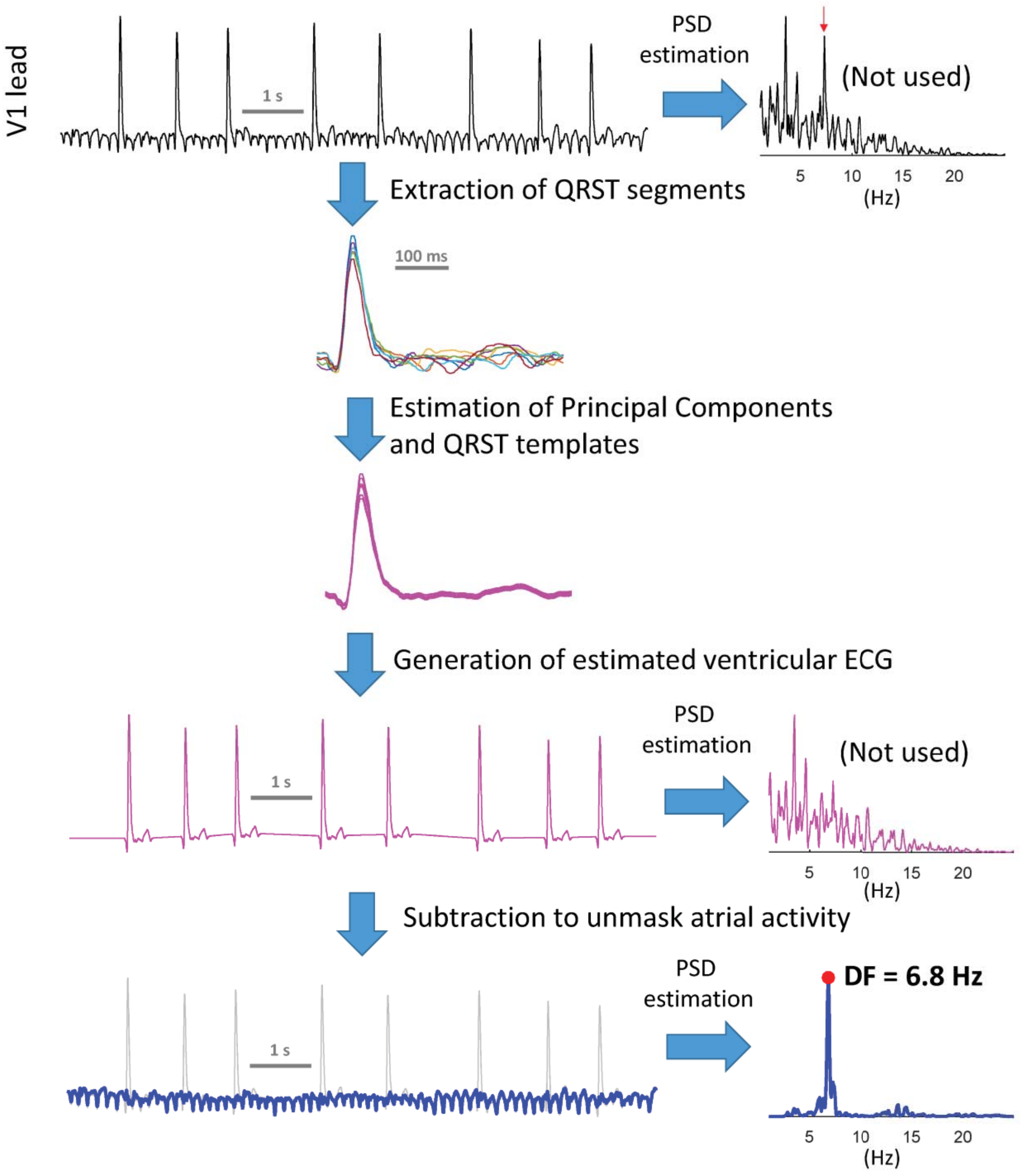

Online Figure XV. QRST subtraction from a sample surface ECG lead in a patient. The estimated ventricular ECG is obtained using PCA, and then subtracted to obtain the atrial activity. In this example, the frequency component that displays atrial activity was clearly visible in the original ECG spectrum (red arrow). However, this is not always the case (see next figure).

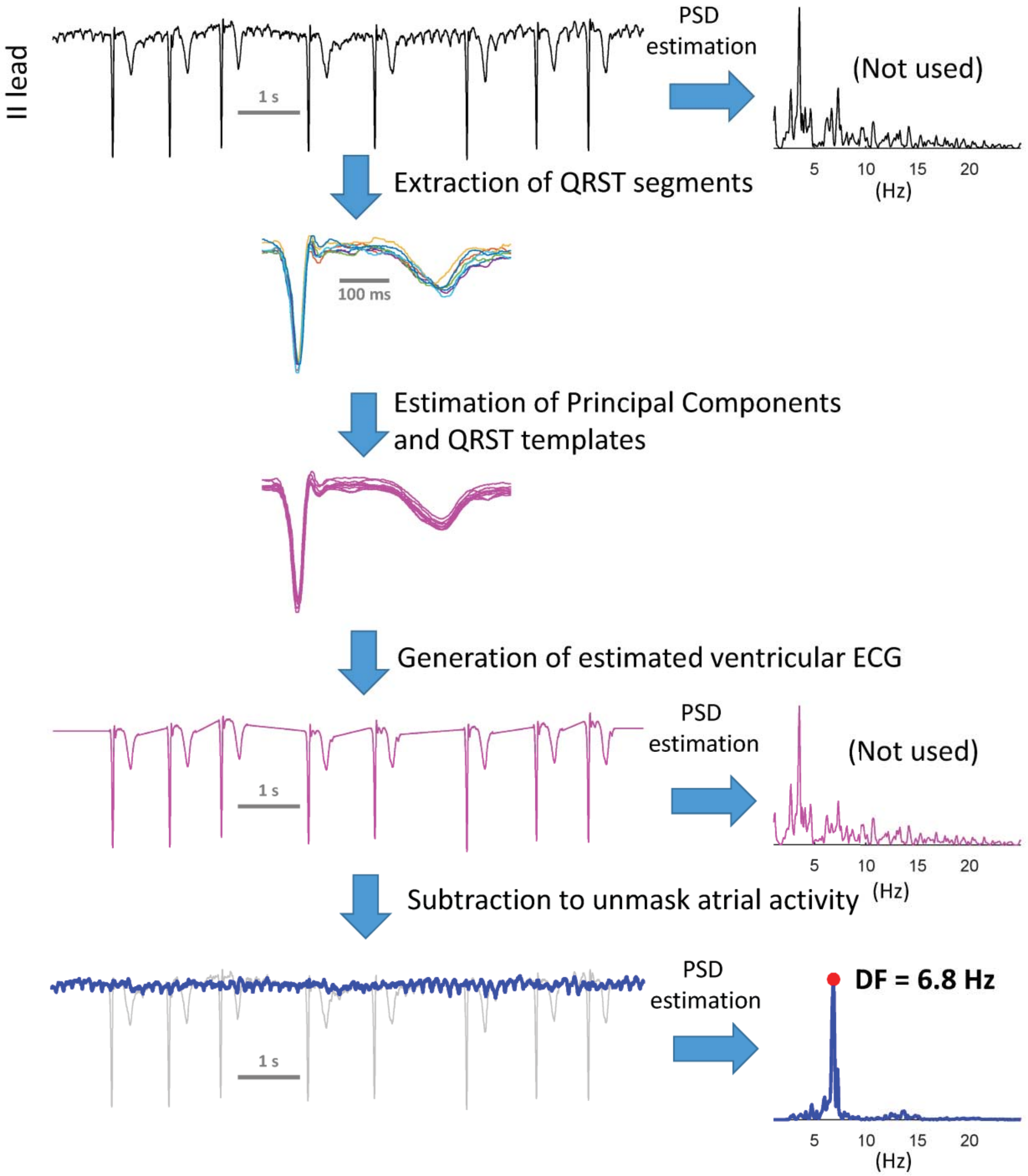

Online Figure XVI. QRST subtraction from a sample surface ECG lead in a patient. The estimated ventricular ECG is obtained using PCA, and then subtracted to obtain the atrial activity. In this example, the frequency component that displays atrial activity was not clearly present in the original ECG spectrum.

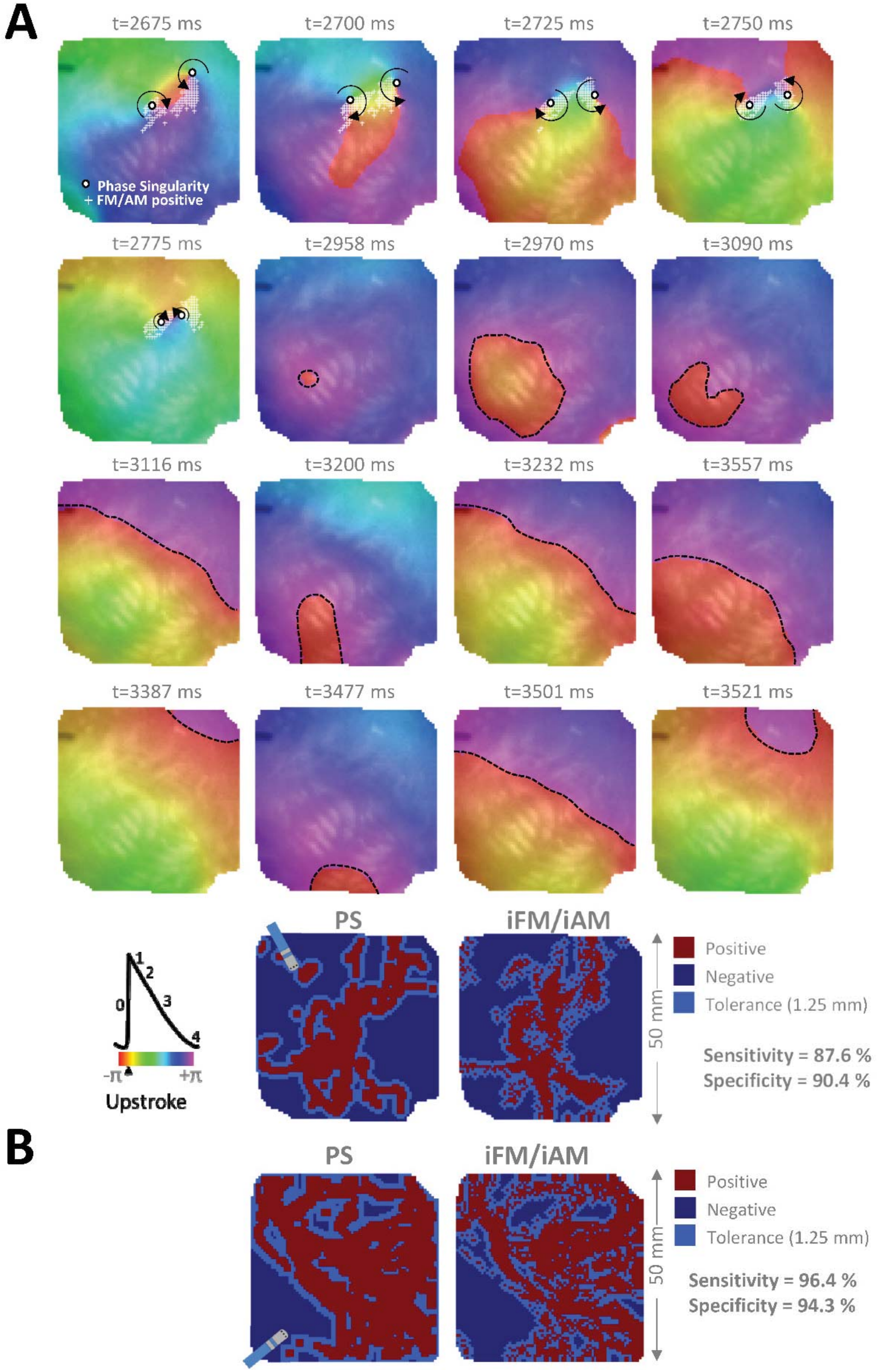

Online Figure XVII. A. Sample snapshots from sheep #4 displaying time intervals with a figure-of-eight reentry, breakthroughs and planar wavefronts in an optical movie (**Online Movie VIII**). Note that the single-signal iFM/iAM algorithm consistently displayed positive rotational-footprints in pixels close to the phase singularities (PS) of the figure-of-eight reentry. Conversely, no positive detections were obtained by the algorithm during time intervals with breakthrough/planar wavefront activations. **B.** SP and iFM/iAM maps of a sample optical mapping movie from sheep #5 (**Online Movie IX**).

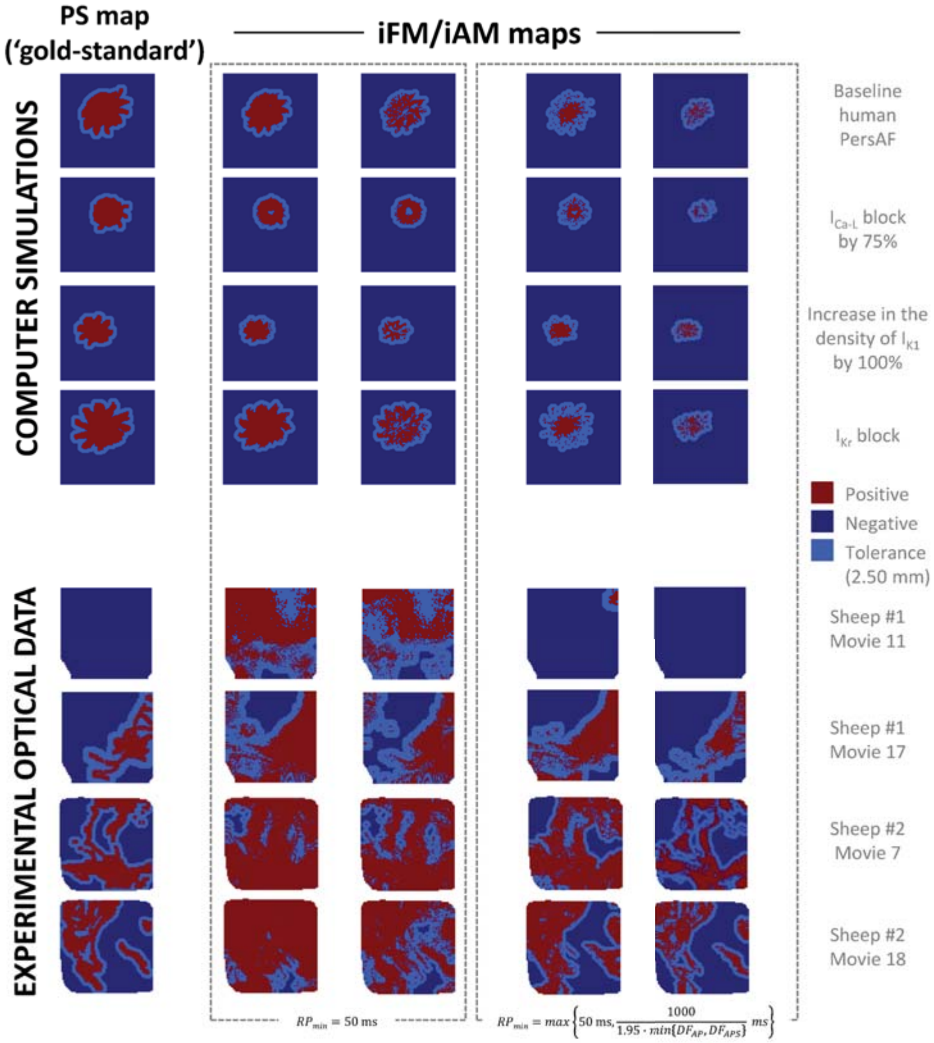

Min. increasing iFM cycles	3	4	3	4
Min. increasing iAM cycles	2	3	2	3
iAM threshold (%)	75	80	75	80
Min. iAM excursion (%)	0	25	0	25
Min. iFM percentile	70	70	70	70
	Optimal for simulations of quasi-stationary rotors			Optimal for optical mapping experimental data

Online Figure XVIII. Examples of the performance of i) the optimal parameters of the iFM/iAM algorithm for the detection of the rotational-footprint of quasi-stationary rotors in computer simulations of PersAF, ii) the optimal parameters of the iFM/iAM algorithm for the detection of the rotational-footprint of varying behavior rotational activity in experimental optical mapping data from PersAF sheep. Both sets of parameters were tested with computer simulations and experimental data, and using either a constant minimum refractory period (RP_{min}) or a signal-specific one. Note that the optimal parameters derived from simulations of quasi-stationary rotors (RP_{min}=50 ms, minimum number of increasing iFM cycles=3, minimum number of increasing iAM cycles=2, iAM threshold=75%, minimum iAM excursion=0%, and minimum iFM percentile=70th) offered very low specificities when applied to experimental data. Conversely, the optimal parameters derived from experimental data (signal-specific RP_{min}, minimum number of increasing iFM cycles=4, minimum number of increasing iAM cycles=3, iAM threshold=80%, minimum iAM excursion=25%, and minimum iFM percentile=70th) offered very high specificity values but lower sensitivity values when applied to simulated quasi-stationary rotors in 2D computational models of PersAF. Nevertheless, although the latter sensitivities were not very high, they were high enough to detect the footprint of the simulated 2D quasi-stationary rotors, while preserving high sensitivities and specificities when used with experimental data.

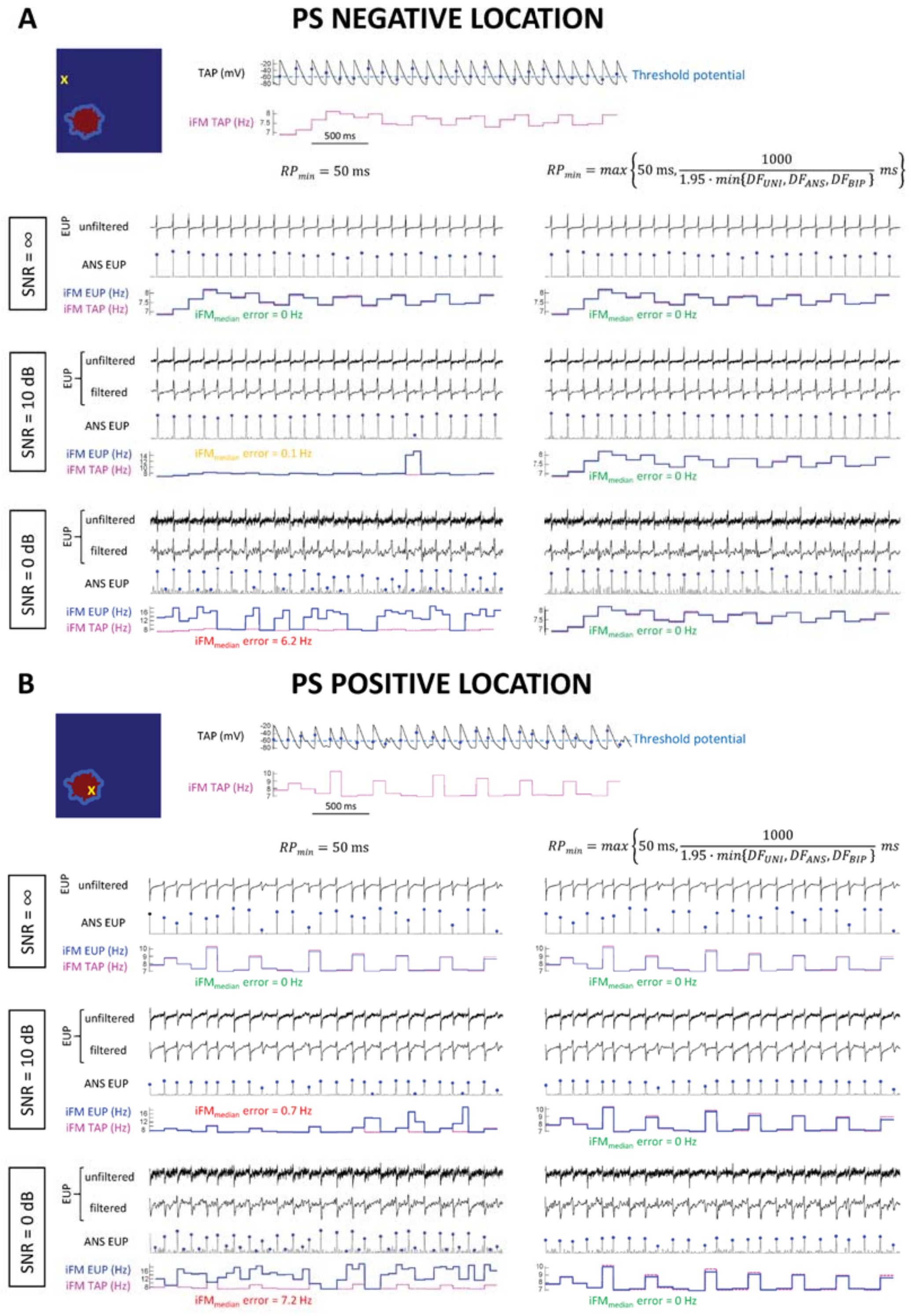

Online Figure XIX. Examples of iFM measurements using 1 mm^2 electrode unipolar potentials (EUP) from a computational sheep model of PersAF in the presence of different levels of additive white gaussian noise. **A.** Signals from a phase singularity (PS) negative location (marked with a yellow 'x'). **B.** Signals from a PS positive location (marked with a yellow 'x'). Note that in the presence of noise, the use of a constant minimum refractory period (RP_{min}) of 50 ms is not optimal since the error in the iFM measurement escalates as noise increases. However, the use of a signal-specific RP_{min} enabled the accurate measurement of iFM in both PS negative and positive locations even in the presence of high levels of noise. See also the "Computer simulations" section in the Expanded Methods for further details about how the iFM reference signal was obtained from the simulated transmembrane action potentials (TAP) devoid of noise.

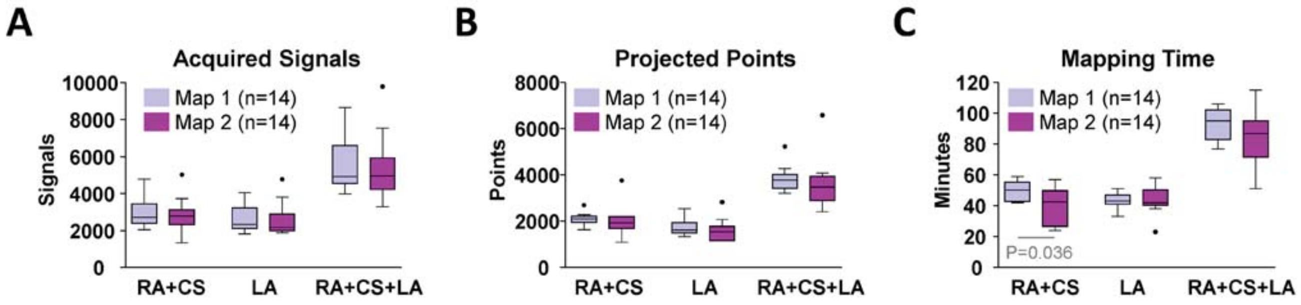

Online Figure XX. Electroanatomical mapping. **A.** Acquired signals. **B.** Number of points projected to the 3D mesh. As expected, the number of projected points is lower than the number of acquired signals. The main reasons for that are: i) signals acquired too far from the surface mesh are not projected, ii) different signals can be acquired at different 3D locations, but these different 3D locations might correspond to the same surface mesh location when projected. In the latter case, the signal that was acquired closest to the mesh was preserved. **C.** Mapping time.

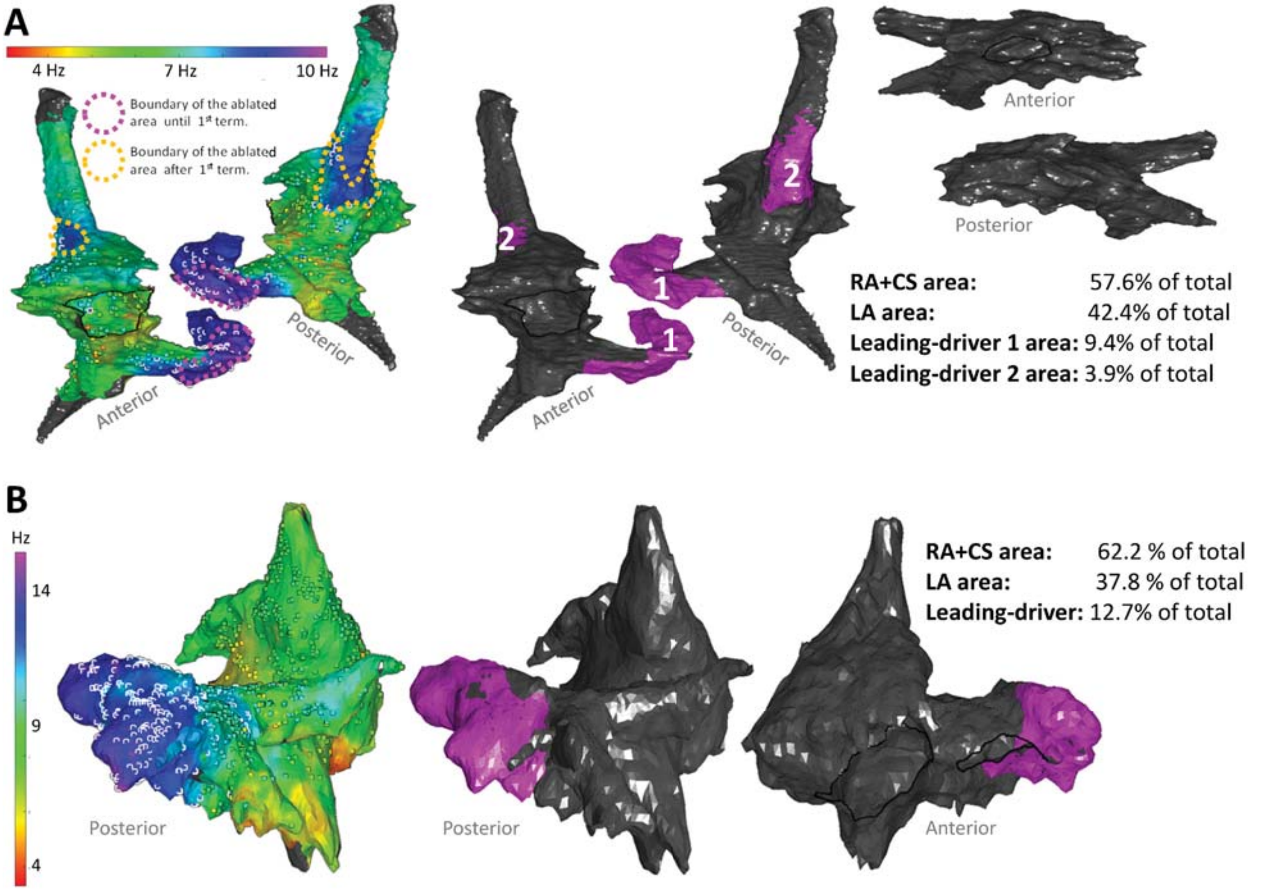

Online Figure XXI. Calculation of the percent of the total atrial surface covered by leading-driver regions. A. Left: iFM_{median} map displayed in **Figure 6A** with two leading-driver regions in the RA and CS. Right: RA+CS constituted the 57.6% of the total atrial area. Leading-driver regions are displayed in fuchsia. The leading-driver region at the CS (label 1) covered the 9.4% of the total atrial surface. The leading-driver region at the insertion of the superior cava vein with the RA (label 2) covered the 3.9% of the total atrial surface. The two leading-driver regions together covered the 13.3% of the total atrial surface. Note that the regions actually targeted for ablation until self-sustained PersAF termination and non-sustainability were smaller than the actual leading-driver regions. B. Left: iFM_{median} map displayed in **Figure 6B** with one leading-driver region in the LA free wall that covered the 12.7% of the total atrial surface.

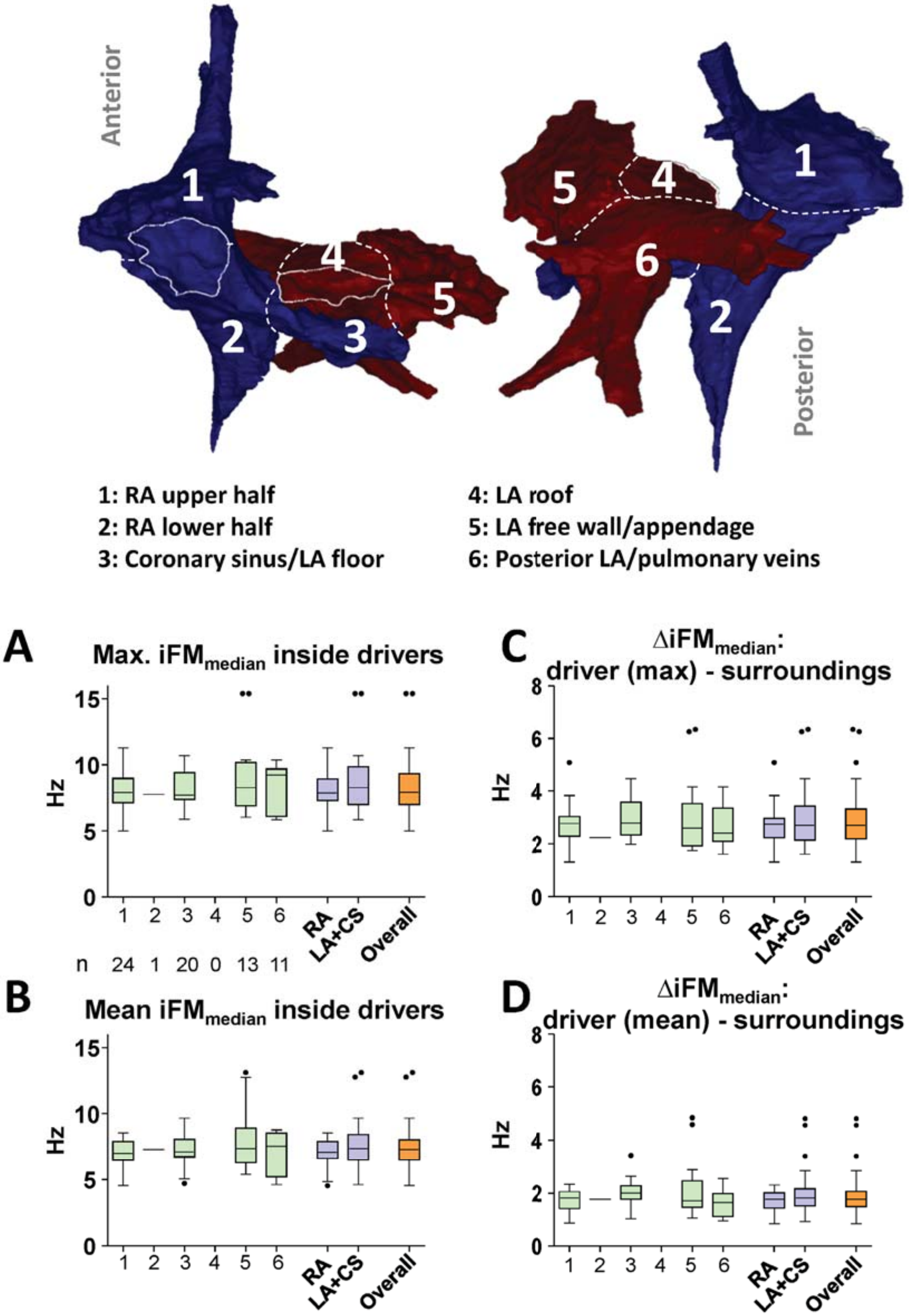

Online Figure XXII. A. Maximum values of iFM_{median} inside leading-driver regions (n=26 biatrial maps, two per mapping + ablation procedure). Note that most outliers correspond to the pig case in **Figure 6B** that shown extremely fast leading-driver regions. **B.** Average values of iFM_{median} inside leading-driver regions. **C.** iFM_{median} gradient between the maximum value inside leading-driver regions and the mean value in the surroundings (non leading-driver regions in the same atrium). **D.** iFM_{median} gradient between the average value inside leading-driver regions and the mean value in the surroundings (non leading-driver regions in the same atrium).

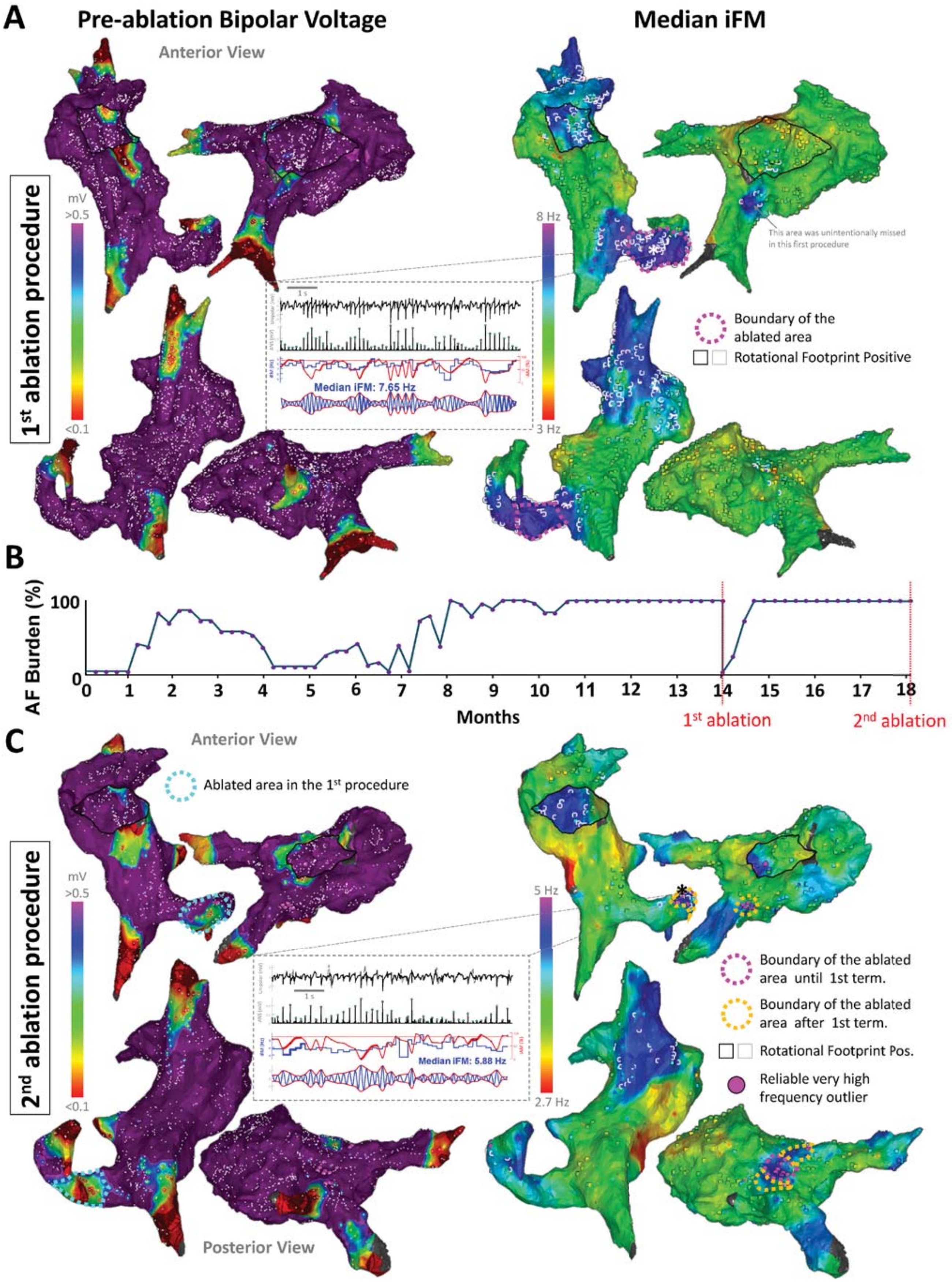
A
Pre-ablation Bipolar Voltage
Median iFM
Anterior View
1st ablation procedure
mV
>0.5
<0.1
8 Hz
3 Hz
Median iFM: 7.65 Hz
This area was unintentionally missed in this first procedure
Boundary of the ablated area
Rotational Footprint Positive
B
AF Burden (%)
Months
1st ablation
2nd ablation
C
Anterior View
Ablated area in the 1st procedure
2nd ablation procedure
5 Hz
2.7 Hz
Median iFM: 5.88 Hz
Boundary of the ablated area until 1st term.
Boundary of the ablated area after 1st term.
Rotational Footprint Pos.
Reliable very high frequency outlier
Posterior View

Online Figure XXIII. Extended version of **panel A** in **Figure 7.** Pre-ablation voltage maps and signals from leading-driver regions are shown additionally. One out of 12 pigs underwent 2 ablation procedures, >4 months apart to demonstrate that acute termination of persistent AF during ablation was the result of a direct modification of the underlying substrate that was maintaining persistent AF. **A.** First ablation procedure. Left panel: pre-ablation bipolar voltage during AF. Both atria mostly consisted of healthy tissue (>0.5 mV). Only venous or peri-annular regions presented bipolar voltages under 0.5 mV. Right panel: persistent AF terminated after ablating a leading driver region in the coronary sinus for ~10 min. Interestingly, a leading region at the ostium of an inferior pulmonary vein and close to the coronary sinus was unintentionally missed. **B.** After the first ablation procedure, the pig was kept alive for 4 more months before a second mapping + ablation procedure. Importantly, after acute termination of persistent AF during the first ablation procedure, AF burden was dramatically reduced despite high rate atrial pacing. It took over 3 weeks to reach 100% AF burden again. These data confirmed that the ablated leading-driver region was contributing to the long-term maintenance of AF. **C.** Second ablation procedure. Left panel: bipolar voltage during AF. In addition to venous and peri-annular regions, the coronary sinus region that was ablated in the first procedure also presented low bipolar voltage values. After mapping, this persistent AF episode showed a leading-driver region at the previously unintentionally missed area. Other leading driver regions were documented at the boundary of the area ablated at the coronary sinus in the previous procedure, and at the posterior left atrium (PLA). Note that, excepting the areas with high iFM_{median} outliers (fuchsia circles), this persistent AF episode was significantly slower than the episode mapped during the first procedure. After radiofrequency delivery at the PLA (framed in fuchsia, 3.4 min), persistent AF acutely terminated. Then, AF was reinduced and lasted >10 minutes. Therefore, the ablation protocol was resumed. After completing radiofrequency delivery at the region on the PLA, the area unintentionally missed in the previous procedure and the outliers at the boundary of the previously ablated tissue in the coronary sinus, AF terminated again (total RF time: 19.8 min). Then, reinduced AF episodes lasted ~8 min. These episodes might have been potentially maintained by the non-ablated region in the RA. However, since these AF episodes lasted <10 min, ablation protocol was not resumed.

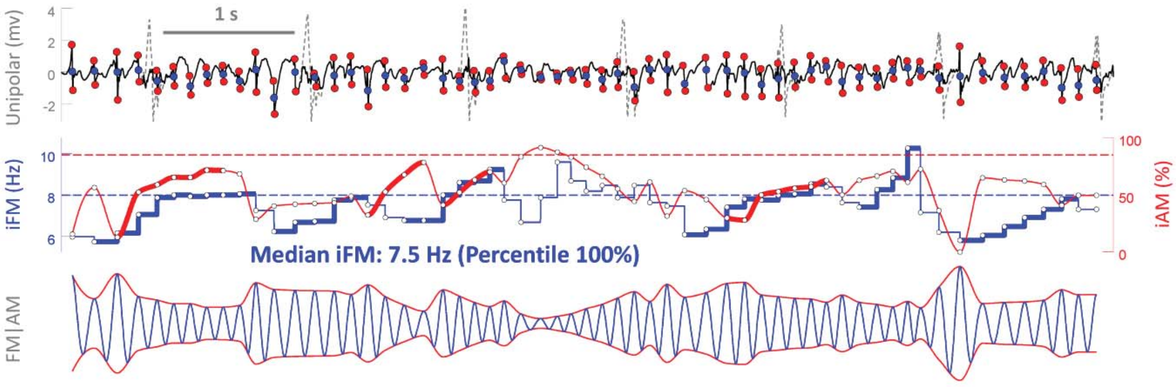

Online Figure XXIV. Sample signal with the maximum iFM_{median} from the map showed in **Figure 7B**. This signal was retrieved from the coronary sinus (marked with a white '*' in **Figure 7B**). Note the presence of 6 intervals of increasing instantaneous frequency modulation for ≥4 cycles (blue thick line). Even though the accompanying iAM did not reach the required 85% threshold to consider that a rotor crossed through the location, these sustained intervals of increasing iFM likely indicate the presence of rotors/scroll waves nearby.

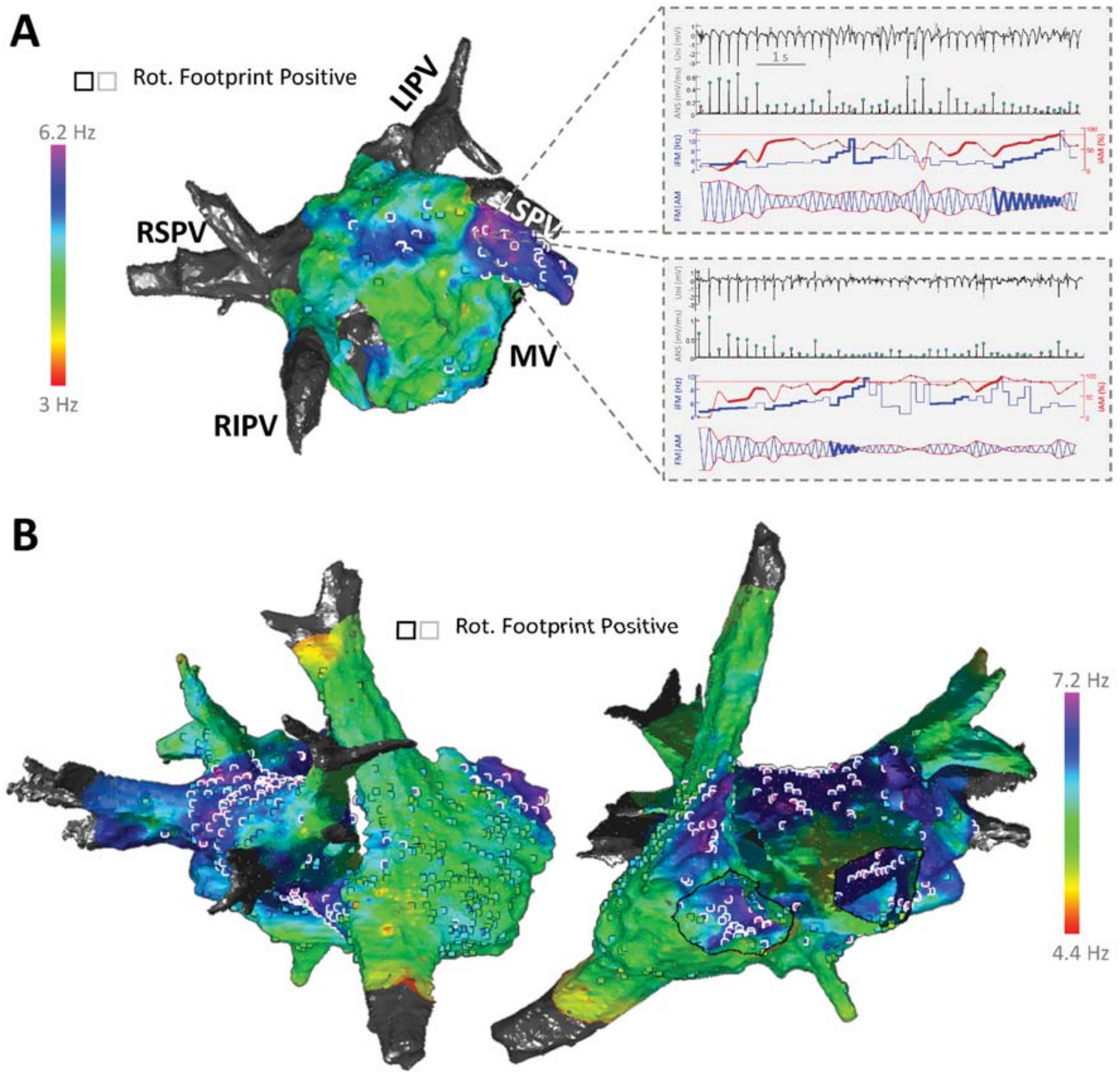

Online Figure XXV. Maps generated using the iFM/iAM approach in 2 PersAF patients without previous pulmonary vein isolation (PVI) procedures (observational group). The maps shown were acquired prior to PVI. Then, the patients were cardioverted and PVI was performed during sinus rhythm. **A.** In this patient, the map displayed a well-delimited leading-driver region whose maximal iFM_{median} values overlapped the anterior region of the left superior pulmonary vein (LSPV) ostium and the proximal part of left atrial appendage (LAA). The signals from 2 high iFM_{median} locations in such a region are also shown. Another islet of higher than surrounding iFM_{median} values was found in the left atrial roof, but it displayed considerably lower values of iFM_{median} than the aforementioned region. The right atrium did not host any leading-driver region (not shown). This patient remains in sinus rhythm after 16 months of follow-up. **B.** In this patient, the map shows high iFM_{median} regions covering a substantial portion of the left atrium, the septum and the right atrial appendage (RAA). Similarly to patient #1 in the interventional group (**Figure 8A**), this map may anticipate the failure of a limited ablation strategy, since large regions of the atria are capable of sustaining high iFM activity. In fact, AF recurrences were observed during the follow-up despite antiarrhythmic drug therapy.

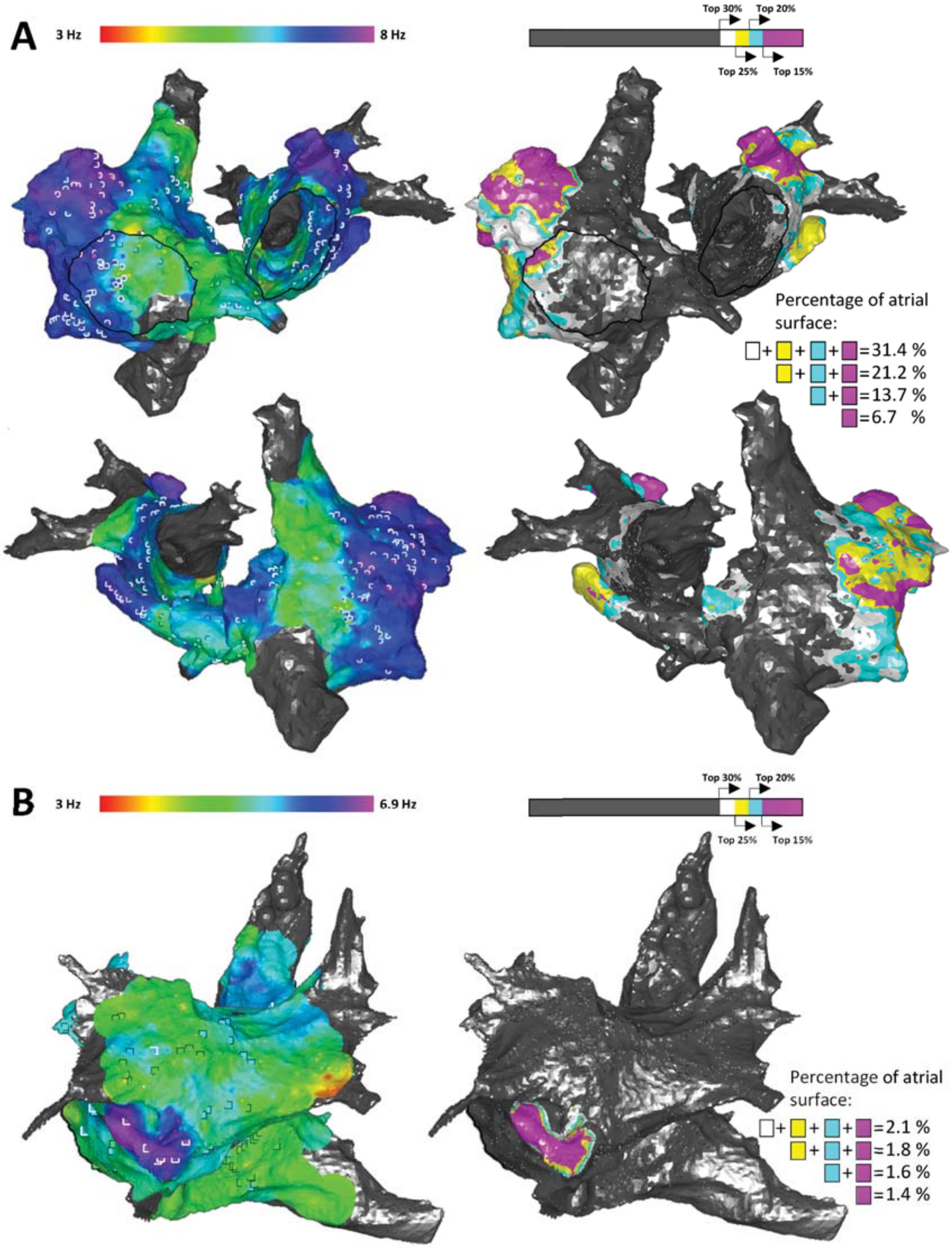

Online Figure XXVI. Boundaries of the leading-driver regions in the maps shown in **Figure 8** using different iFM_{median} thresholds. **A.** Left panel: iFM_{median} + rotational-footprint map shown in **Figure 8A**. Our usual definition of leading-driver region (locations with iFM_{median} values within the top 30% in the color scale) resulted in a large area that covered 31.4% of the total atrial surface. If we had selected the locations hosting the top 15% values in the color scale, the target region would have covered only 6.7% of the total atrial surface. Such a region would have mainly consisted of the LAA, the RAA and a small portion of the RA free wall. We cannot exclude that the clinical outcomes of this patient would have been improved if all the magenta region had been fully ablated. However, such areas are still difficult to ablate using a catheter-based approach. **B.** Left panel: iFM_{median} + rotational-footprint map shown in **Figure 8B**. In this map, changing the iFM_{median} threshold to select the target region has little impact from a practical point of view. We speculate that maps with clearly delimited 'islets' of high iFM_{median} like this one, may predict better outcomes after limited catheter ablation.

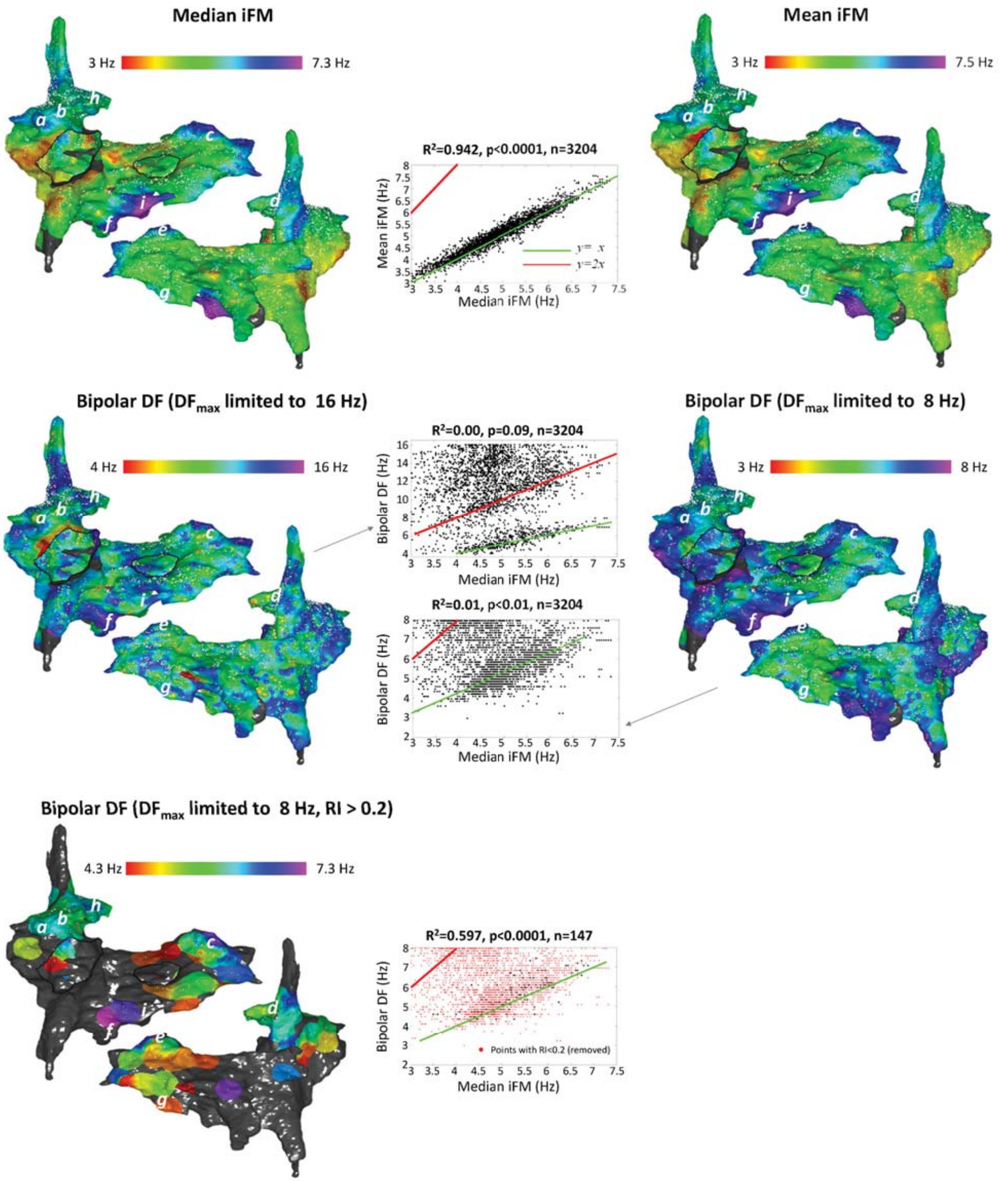

Online Figure XXVII. Comparison of the approaches to detect hierarchically relevant driver regions in persistent AF: 1) Median iFM, 2) Mean iFM, 3) Bipolar DF (upper limit for DF: 16 Hz), 4) Bipolar DF (upper limit for DF: 8 Hz) and 5) Bipolar DF (upper limit for DF: 8 Hz, only points with regularity index RI≥0.2). *a, b, c, d, e, f, g, h, i* display the locations from where the signals shown in **Online Figure XXVIII** were retrieved. See text for details.

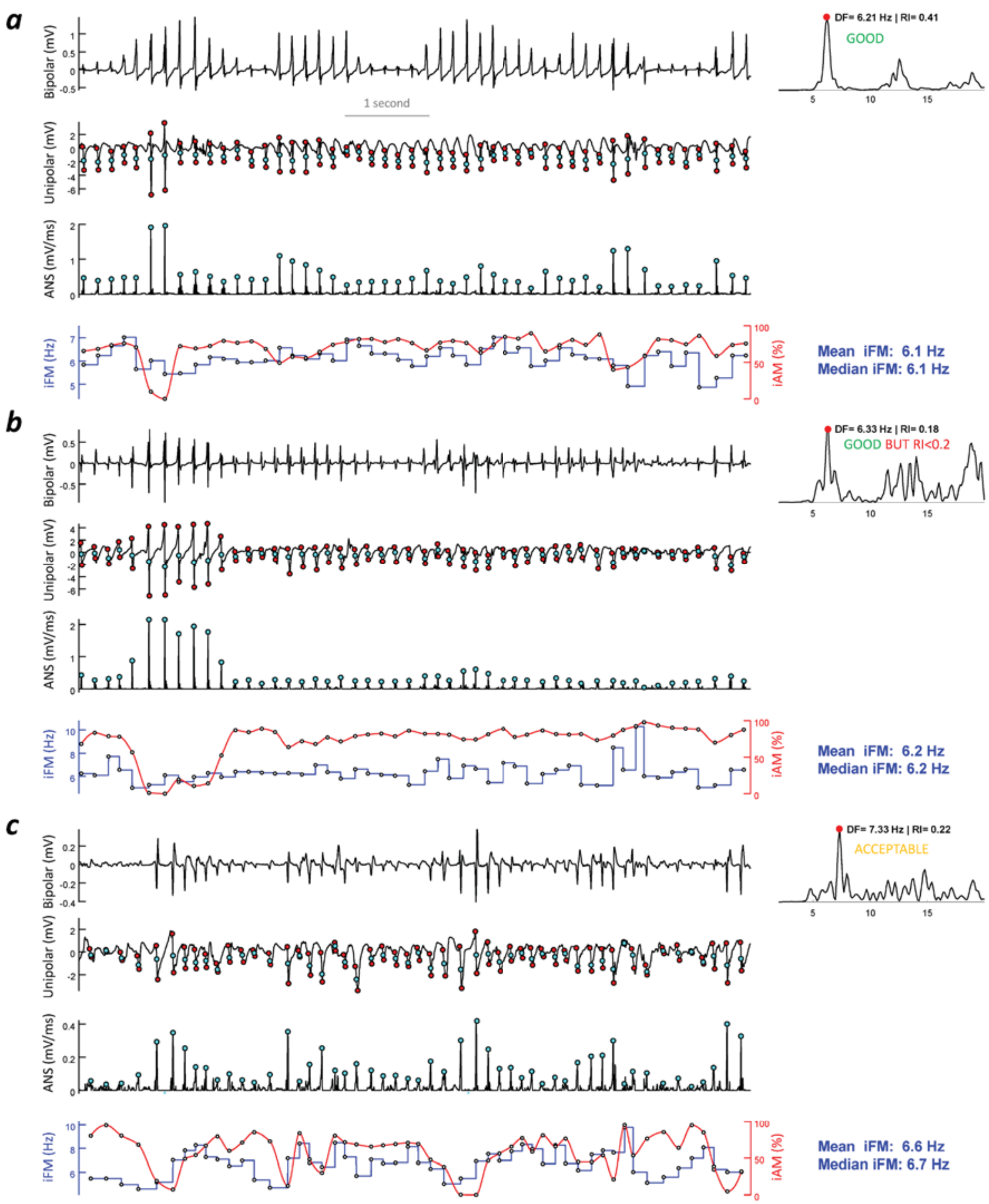

Online Figure XXVIII. Sample signals from different atrial regions that illustrate the limitations of the dominant frequency approach to detect leading-driver regions during persistent AF. The first row in each panel shows the bipolar signal, its power spectral density, dominant frequency (DF) and regularity index (RI). The second row displays the unipolar signal after ventricular far-field minimization. The third row shows the absolute negative slope (ANS) signal after ventricular far-field residue rejection. The cyan circles in the ANS signal show the activations to calculate the instantaneous frequency modulation (iFM) signal shown in the fourth row (in blue). Red circles in the third row display the amplitude values of the unipolar signal used to calculate the instantaneous amplitude modulation (iAM, red trace in the fourth row). Also, the mean and median iFM values are shown to summarize the local average activation rate. **a.** Signal with a good correlation between Bipolar DF and median/mean iFM. **b.** Signal with a good correlation between bipolar DF and median/mean iFM. However, the RI was <0.2 because of the harmonic peaks. The latter would preclude including this signal in the map to guide ablation using the previously reported DF mapping approach. **c.** Signal with an acceptable correlation between Bipolar DF and median/mean iFM. In this case, the RI was >0.2.

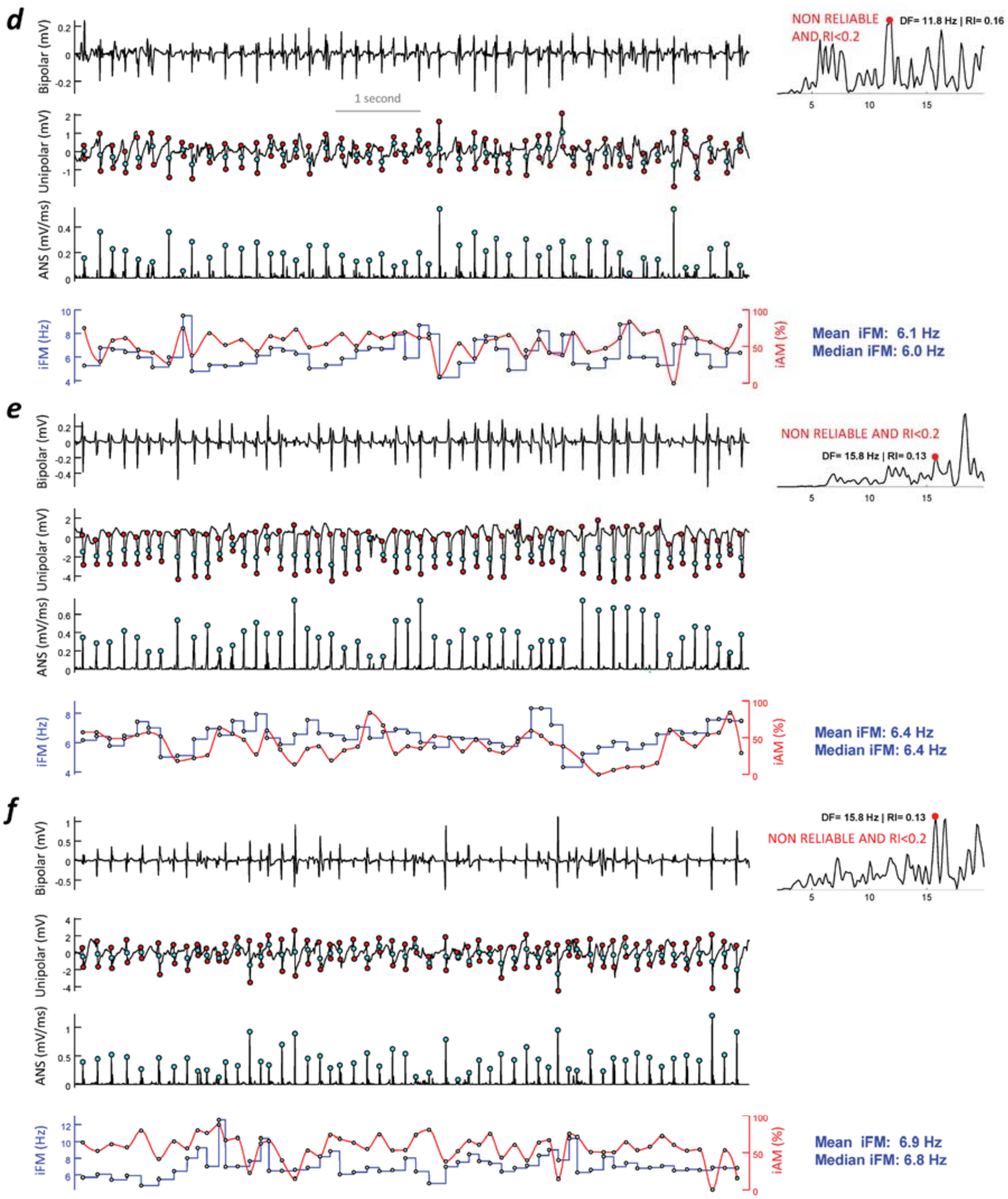

Online Figure XXVIII (continued). d. Signal with a very bad correlation between Bipolar DF and median/mean iFM because the second harmonic was selected as DF. Using 8 Hz as the upper limit for DF, bipolar DF decreased from 11.8 to 5.7 Hz and RI decreased from 0.16 to 0.10. Note that even showing an improved correlation (bipolar DF=5.7 Hz, median iFM=6.0 Hz), a RI<0.2 would preclude including this signal in the map to guide ablation using the previously reported DF mapping approach. **e.** Signal with a very bad correlation between Bipolar DF and median/mean iFM. Using 8 Hz as the upper limit for DF, bipolar DF decreased from 15.8 to 6.8 Hz and RI decreased from 0.13 to 0.06. **f.** Signal with a very bad correlation between Bipolar DF and median/mean iFM. Using 8 Hz as the upper limit for DF, bipolar DF decreased from 15.8 to 7.2 Hz and RI decreased from 0.13 to 0.07.

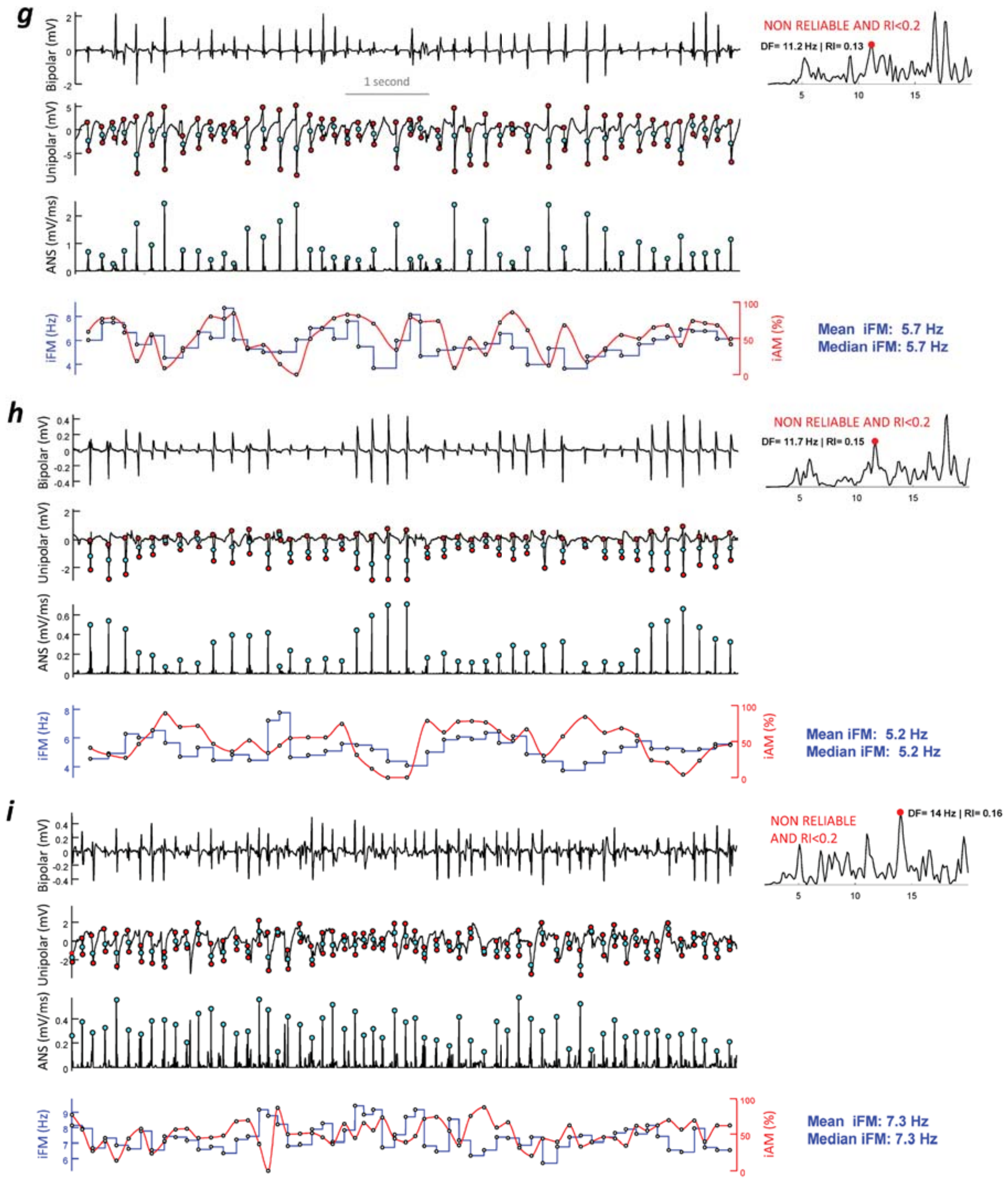

Online Figure XXVIII (continued). g. Signal with a very bad correlation between Bipolar DF and median/mean iFM. Using 8 Hz as the upper limit for DF, bipolar DF decreased from 11.2 to 5.2 Hz and RI decreased from 0.13 to 0.08. **h.** Signal with a very bad correlation between Bipolar DF and median/mean iFM. Using 8 Hz as the upper limit for DF, bipolar DF decreased from 11.7 to 5.8 Hz and RI decreased from 0.15 to 0.10. **i.** Signal with a very bad correlation between Bipolar DF and median/mean iFM. Using 8 Hz as the upper limit for DF, bipolar DF decreased from 14.0 to 5.1 Hz and RI decreased from 0.16 to 0.07. Note that this point was one of those with the highest median iFM (7.3 Hz, maximum value) and was located within the region that was ablated prior to termination of persistent AF. However, bipolar DF was only 5.1 Hz (at best) in that location. Therefore, it would have not been identified as a relevant target using a DF approach.[37, 41]

SUPPLEMENTAL MOVIES

Online Movie I. PersAF phase movie from sheep #2. This movie is associated to **Figure 2** in the main manuscript.

Online Movie II. Fragment from **Online Movie I** associated to **Online Figure II**. Multiple depolarization attempts/partial depolarizations are present in an optical signal during the intervals in which phase singularities meander through the pixel surroundings. They are the result of the rotational wavefront crossing the pixel repeatedly in a seemingly very high non-physiological rate. In this movie, 7 depolarization attempts / partial depolarizations occurred in less than 600 ms, which could be considered as a non-physiological activation rate.

Online Movie III. Phase movie obtained after the interpolation of the electrical data from the 20 poles of a PentaRay catheter fully deployed into the right atrial appendage of a pig with PersAF. The movie is associated to **Figure 3** in the main manuscript. Note the high correlation between the location of rotational-footprint positive electrodes detected by the single-signal iFM/iAM algorithm (highlighted in cyan) and the core of the rotors displayed in the phase movie. Despite rotational activations were repeatedly detected in the right atrium, it was a low-hierarchy region and it did not seem related to long-term maintenance of PersAF. Indeed, PersAF terminated and was rendered non-inducible after ablating only the leading-driver region in the coronary sinus.

Online Movie IV. Phase movie obtained after the interpolation of the electrical data from the 20 poles of a PentaRay catheter fully deployed into the left atrial appendage of the same pig with PersAF displayed in **Online Movie III** and **Figure 3**. Same comments as in **Online Movie III** also apply to **Online Movie IV**.

Online Movie V. PersAF phase movie from sheep #1. The movie is associated to **Figure 4A** in the main manuscript and to **Online Figure IV A** in the Online Supplement.

Online Movie VI. PersAF phase movie from sheep #2. The movie is associated to **Figure 4B** in the main manuscript and to **Online Figure IV B** in the Online Supplement.

Online Movie VII. PersAF phase movie from sheep #3. The movie is associated to **Figure 4C** in the main manuscript.

Online Movie VIII. PersAF phase movie from sheep #4. The movie is associated to **Online Figures IV C** and **XVII A** in the Online Supplement.

Online Movie IX. PersAF phase movie from sheep #5. The movie is associated to **Online Figure XVII B** in the Online Supplement.

In **Online Movies I** and **V-IX**, white dots display phase singularities and white '+' display rotational-footprint positive pixels according to the iFM/iAM algorithm. Observe the high correlation between the location of pixels tagged as rotational-footprint positive by the iFM/iAM algorithm and the phase singularities that eventually appeared in those regions. Note that the iFM/iAM algorithm usually anticipates the presence of phase singularities at any particular spot since the simultaneous increase of iAM and iFM can be detected before the phase singularity actually appears at such spot. Some phase singularities can drift or meander on the filmed surface if the filament of the underlying scroll wave is actually perpendicular to the filmed surface. Others can suddenly appear or disappear because the underlying scroll wave filament is only temporarily perpendicular to the filmed surface. Indeed, white '+' in some areas with seemingly breakthrough activation anticipates their conversion into figure-of-eight reentries. Note that if an intramural scroll-wave filament is not perpendicular to the filmed surface, and therefore a phase singularity is not detected, the translation of this filament can equally result in iAM and iFM simultaneous increases when it approaches the filmed surface.

SUPPLEMENTAL REFERENCES

1. Filgueiras-Rama D, Price NF, Martins RP, Yamazaki M, Avula UM, Kaur K, Kalifa J, Ennis SR, Hwang E, Devabhaktuni V, Jalife J, Berenfeld O. Long-term frequency gradients during persistent atrial fibrillation in sheep are associated with stable sources in the left atrium. *Circ Arrhythm Electrophysiol.* 2012;5:1160-1167.
2. Martins RP, Kaur K, Hwang E*, et al.* Dominant frequency increase rate predicts transition from paroxysmal to long-term persistent atrial fibrillation. *Circulation.* 2014;129:1472-1482.
3. Bauer A, McDonald AD, Donahue JK. Pathophysiological findings in a model of persistent atrial fibrillation and severe congestive heart failure. *Cardiovasc Res.* 2004;61:764-770.
4. Kuklik P, Zeemering S, van Hunnik A*, et al.* Identification of rotors during human atrial fibrillation using contact mapping and phase singularity detection: Technical considerations. *IEEE Trans Biomed Eng.* 2017;64:310-318.
5. Roney CH, Cantwell CD, Bayer JD, Qureshi NA, Lim PB, Tweedy JH, Kanagaratnam P, Peters NS, Vigmond EJ, Ng FS. Spatial resolution requirements for accurate identification of drivers of atrial fibrillation. *Circ Arrhythm Electrophysiol.* 2017;10:e004899.
6. Walters TE, Lee G, Spence S, Kalman JM. The effect of electrode density on the interpretation of atrial activation patterns in epicardial mapping of human persistent atrial fibrillation. *Heart Rhythm.* 2016;13:1215-1220.
7. Berenfeld O. To the editor- concerns in multi-electrode and phase mapping of AF. *Heart Rhythm.* 2018;15:e3-e4.
8. Filgueiras-Rama D, Martins RP, Mironov S, Yamazaki M, Calvo CJ, Ennis SR, Bandaru K, Noujaim SF, Kalifa J, Berenfeld O, Jalife J. Chloroquine terminates stretch-induced atrial fibrillation more effectively than flecainide in the sheep heart. *Circ Arrhythm Electrophysiol.* 2012;5:561-570.
9. Quintanilla JG, Moreno J, Archondo T, Chin A, Pérez-Castellano N, Usandizaga E, García-Torrent MJ, Molina-Morúa R, González P, Rodríguez-Bobada C, Macaya C, Pérez-Villacastín J. K_{ATP} channel opening accelerates and stabilizes rotors in a swine heart model of ventricular fibrillation. *Cardiovasc Res.* 2013;99:576-585.
10. Quintanilla JG, Moreno J, Archondo T, Usandizaga E, Molina-Morúa R, Rodríguez-Bobada C, González P, García-Torrent MJ, Filgueiras-Rama D, Pérez-Castellano N, Macaya C, Pérez-Villacastín J. Increased intraventricular pressures are as harmful as the electrophysiological substrate of heart failure in favoring sustained reentry in the swine heart. *Heart Rhythm.* 2015;12:2172-2183.
11. Quintanilla JG, Moreno J, Archondo T*, et al.* QRS duration reflects underlying changes in conduction velocity during increased intraventricular pressure and heart failure. *Prog Biophys Mol Biol.* 2017;130:394-403.
12. Lee P, Quintanilla JG, Alfonso-Almazán JM, Galán-Arriola C, Yan P, Sánchez-González J, Pérez-Castellano N, Pérez-Villacastín J, Ibáñez B, Loew LM, Filgueiras-Rama D. In-vivo ratiometric optical mapping enables high-resolution cardiac electrophysiology in pig models. *Cardiovasc Res.* 2019
13. Bray MA, Wikswo JP. Considerations in phase plane analysis for nonstationary reentrant cardiac behavior. *Phys Rev E Stat Nonlin Soft Matter Phys.* 2002;65:051902.
14. Gray RA, Pertsov AM, Jalife J. Spatial and temporal organization during cardiac fibrillation. *Nature.* 1998;392:75-78.
15. Iyer AN, Gray RA. An experimentalist's approach to accurate localization of phase singularities during reentry. *Ann Biomed Eng.* 2001;29:47-59.
16. Ng J, Sehgal V, Ng JK, Gordon D, Goldberger JJ. Iterative method to detect atrial activations and measure cycle length from electrograms during atrial fibrillation. *IEEE Trans Biomed Eng.* 2014;61:273-278.
17. Allessie MA, de Groot NM, Houben RP, Schotten U, Boersma E, Smeets JL, Crijns HJ. Electropathological substrate of long-standing persistent atrial fibrillation in patients with structural heart disease: Longitudinal dissociation. *Circ Arrhythm Electrophysiol.* 2010;3:606-615.

18. de Groot NM, Houben RP, Smeets JL, Boersma E, Schotten U, Schalij MJ, Crijns H, Allessie MA. Electropathological substrate of longstanding persistent atrial fibrillation in patients with structural heart disease: Epicardial breakthrough. *Circulation.* 2010;122:1674-1682.
19. Moreno J, Quintanilla JG, Molina-Morúa R*, et al.* Morphological and thermodynamic comparison of the lesions created by 4 open-irrigated catheters in 2 experimental models. *J Cardiovasc Electrophysiol.* 2014;25:1391-1399.
20. Alfonso-Almazán JM, Quintanilla JG, García-Torrent MJ*, et al.* Lesion index titration using contact-force technology enables safe and effective radiofrequency lesion creation at the root of the aorta and pulmonary artery. *Circ Arrhythm Electrophysiol.* 2019;12:e007080.
21. Pandit SV, Berenfeld O, Anumonwo JM, Zaritski RM, Kneller J, Nattel S, Jalife J. Ionic determinants of functional reentry in a 2-d model of human atrial cells during simulated chronic atrial fibrillation. *Biophys J.* 2005;88:3806-3821.
22. Takemoto Y, Ramirez RJ, Yokokawa M*, et al.* Galectin-3 regulates atrial fibrillation remodeling and predicts catheter ablation outcomes. *JACC Basic Transl Sci.* 2016;1:143-154.
23. Courtemanche M, Ramirez RJ, Nattel S. Ionic mechanisms underlying human atrial action potential properties: Insights from a mathematical model. *Am J Physiol.* 1998;275:H301-321.
24. Grandi E, Pandit SV, Voigt N, Workman AJ, Dobrev D, Jalife J, Bers DM. Human atrial action potential and Ca^{2+} model: Sinus rhythm and chronic atrial fibrillation. *Circ Res.* 2011;109:1055-1066.
25. Aizer A, Holmes DS, Garlitski AC, Bernstein NE, Smyth-Melsky JM, Ferrick AM, Chinitz LA. Standardization and validation of an automated algorithm to identify fractionation as a guide for atrial fibrillation ablation. *Heart Rhythm.* 2008;5:1134-1141.
26. Lee G, Kumar S, Teh A*, et al.* Epicardial wave mapping in human long-lasting persistent atrial fibrillation: Transient rotational circuits, complex wavefronts, and disorganized activity. *Eur Heart J.* 2014;35:86-97.
27. Lee S, Ryu K, Waldo AL, Khrestian CM, Durand DM, Sahadevan J. An algorithm to measure beat-to-beat cycle lengths for assessment of atrial electrogram rate and regularity during atrial fibrillation. *J Cardiovasc Electrophysiol.* 2013;24:199-206.
28. Correa de Sa DD, Thompson N, Stinnett-Donnelly J, Znojkiewicz P, Habel N, Muller JG, Bates JH, Buzas JS, Spector PS. Electrogram fractionation: The relationship between spatiotemporal variation of tissue excitation and electrode spatial resolution. *Circ Arrhythm Electrophysiol.* 2011;4:909-916.
29. Narayan SM, Krummen DE, Shivkumar K, Clopton P, Rappel WJ, Miller JM. Treatment of atrial fibrillation by the ablation of localized sources: CONFIRM trial. *J Am Coll Cardiol.* 2012;60:628-636.
30. Haissaguerre M, Hocini M, Denis A*, et al.* Driver domains in persistent atrial fibrillation. *Circulation.* 2014;130:530-538.
31. Calvo D, Rubín J, Pérez D, Morís C. Ablation of rotor domains effectively modulates dynamics of human long-standing persistent atrial fibrillation. *Circ Arrhythm Electrophysiol.* 2017;10: e005740
32. Rodrigo M, Climent AM, Liberos A, Fernández-Avilés F, Berenfeld O, Atienza F, Guillem MS. Technical considerations on phase mapping for identification of atrial reentrant activity in direct- and inverse-computed electrograms. *Circ Arrhythm Electrophysiol.* 2017;10
33. Kuklik P, Zeemering S, Maesen B, Maessen J, Crijns HJ, Verheule S, Ganesan AN, Schotten U. Reconstruction of instantaneous phase of unipolar atrial contact electrogram using a concept of sinusoidal recomposition and hilbert transform. *IEEE Trans Biomed Eng.* 2015;62:296-302.
34. Pathik B, Kalman JM, Walters T, Kuklik P, Zhao J, Madry A, Sanders P, Kistler PM, Lee G. Absence of rotational activity detected using 2-dimensional phase mapping in the corresponding 3-dimensional phase maps in human persistent atrial fibrillation. *Heart Rhythm.* 2018;15:182-192.
35. Pathik B, Kalman JM, Walters T, Kuklik P, Zhao J, Madry A, Prabhu S, Nalliah C, Kistler P, Lee G. Transient rotor activity during prolonged 3-dimensional phase mapping in human persistent atrial fibrillation. *JACC Clin Electrophysiol.* 2018;4:72-83.
36. Vidmar D, Rappel WJ. To the editor- on the deformation and interpolation of phase maps. *Heart Rhythm.* 2018;15:e3.

37. Atienza F, Almendral J, Ormaetxe JM*, et al.* Comparison of radiofrequency catheter ablation of drivers and circumferential pulmonary vein isolation in atrial fibrillation: A noninferiority randomized multicenter RADAR-AF trial. *J Am Coll Cardiol.* 2014;64:2455-2467.
38. Ng J, Goldberger JJ. Understanding and interpreting dominant frequency analysis of af electrograms. *J Cardiovasc Electrophysiol.* 2007;18:680-685.
39. Ng J, Kadish AH, Goldberger JJ. Effect of electrogram characteristics on the relationship of dominant frequency to atrial activation rate in atrial fibrillation. *Heart Rhythm.* 2006;3:1295-1305.
40. Ng J, Kadish AH, Goldberger JJ. Technical considerations for dominant frequency analysis. *J Cardiovasc Electrophysiol.* 2007;18:757-764.
41. Atienza F, Almendral J, Jalife J, Zlochiver S, Ploutz-Snyder R, Torrecilla EG, Arenal A, Kalifa J, Fernández-Avilés F, Berenfeld O. Real-time dominant frequency mapping and ablation of dominant frequency sites in atrial fibrillation with left-to-right frequency gradients predicts long-term maintenance of sinus rhythm. *Heart Rhythm.* 2009;6:33-40.
42. Fischer G, Hintringer F. Letter regarding article by sanders et al, "spectral analysis identifies sites of high-frequency activity maintaining atrial fibrillation in humans". *Circulation.* 2006;113:e44, author reply e44-45.
43. Seitz J, Bars C, Theodore G*, et al.* AF ablation guided by spatiotemporal electrogram dispersion without pulmonary vein isolation: A wholly patient-tailored approach. *J Am Coll Cardiol.* 2017;69:303-321.
44. Everett TH 4th, Kok LC, Vaughn RH, Moorman JR, Haines DE. Frequency domain algorithm for quantifying atrial fibrillation organization to increase defibrillation efficacy. *IEEE Trans Biomed Eng.* 2001;48:969-978.
